# Deep learning algorithm performance in contouring head and neck organs at risk: a systematic review and single-arm meta-analysis

**DOI:** 10.1186/s12938-023-01159-y

**Published:** 2023-11-01

**Authors:** Peiru Liu, Ying Sun, Xinzhuo Zhao, Ying Yan

**Affiliations:** 1 General Hospital of Northern Theater Command, Department of Radiation Oncology, Shenyang, China; 2 Beifang Hospital of China Medical University, Shenyang, China; 3 Shenyang University of Technology, School of Electrical Engineering,, Shenyang, China

**Keywords:** Deep learning, Organs at risk, Head and neck cancer, Contouring, Systematic review, Meta-analysis

## Abstract

**Purpose:**

The contouring of organs at risk (OARs) in head and neck cancer radiation treatment planning is a crucial, yet repetitive and time-consuming process. Recent studies have applied deep learning (DL) algorithms to automatically contour head and neck OARs. This study aims to conduct a systematic review and meta-analysis to summarize and analyze the performance of DL algorithms in contouring head and neck OARs. The objective is to assess the advantages and limitations of DL algorithms in contour planning of head and neck OARs.

**Methods:**

This study conducted a literature search of Pubmed, Embase and Cochrane Library databases, to include studies related to DL contouring head and neck OARs, and the dice similarity coefficient (DSC) of four categories of OARs from the results of each study are selected as effect sizes for meta-analysis. Furthermore, this study conducted a subgroup analysis of OARs characterized by image modality and image type.

**Results:**

149 articles were retrieved, and 22 studies were included in the meta-analysis after excluding duplicate literature, primary screening, and re-screening. The combined effect sizes of DSC for brainstem, spinal cord, mandible, left eye, right eye, left optic nerve, right optic nerve, optic chiasm, left parotid, right parotid, left submandibular, and right submandibular are 0.87, 0.83, 0.92, 0.90, 0.90, 0.71, 0.74, 0.62, 0.85, 0.85, 0.82, and 0.82, respectively. For subgroup analysis, the combined effect sizes for segmentation of the brainstem, mandible, left optic nerve, and left parotid gland using CT and MRI images are 0.86/0.92, 0.92/0.90, 0.71/0.73, and 0.84/0.87, respectively. Pooled effect sizes using 2D and 3D images of the brainstem, mandible, left optic nerve, and left parotid gland for contouring are 0.88/0.87, 0.92/0.92, 0.75/0.71 and 0.87/0.85.

**Conclusions:**

The use of automated contouring technology based on DL algorithms is an essential tool for contouring head and neck OARs, achieving high accuracy, reducing the workload of clinical radiation oncologists, and providing individualized, standardized, and refined treatment plans for implementing "precision radiotherapy". Improving DL performance requires the construction of high-quality data sets and enhancing algorithm optimization and innovation.

**Supplementary Information:**

The online version contains supplementary material available at 10.1186/s12938-023-01159-y.

## Introduction

Head and neck cancer is a highly malignant cancer with significant morbidity and mortality rates globally [1]. It comprises various types, such as nasopharyngeal, oropharyngeal, hypopharyngeal, and laryngeal cancers, all of which differ significantly in terms of clinical features, treatment, and prognosis [1]. The epidemiology of head and neck cancer differs based on ethnicity, nationality, gender, and age groups [2–4]. Tobacco and alcohol consumption, along with HPV infection, represent the primary risk factors for head and neck cancer. Specifically, HPV-16 seropositivity is associated with a nearly 30-fold higher risk of pharyngeal cancer [5–7]. Radiotherapy is a critical component of comprehensive treatment for head and neck cancer. Techniques such as 3D conformal radiotherapy, stereotactic radiotherapy, and intensity-modulated radiotherapy are commonly used for treating head and neck cancer. However, radiotherapy can also result in adverse effects, including xerostomia [8, 9], dysphagia [10, 11] and radiation osteonecrosis [12]. Accurate OAR contouring in the head and neck region can significantly reduce the incidence of adverse effects of radiotherapy, which will directly impact tumor control and long-term prognosis.

**Fig. 1:**
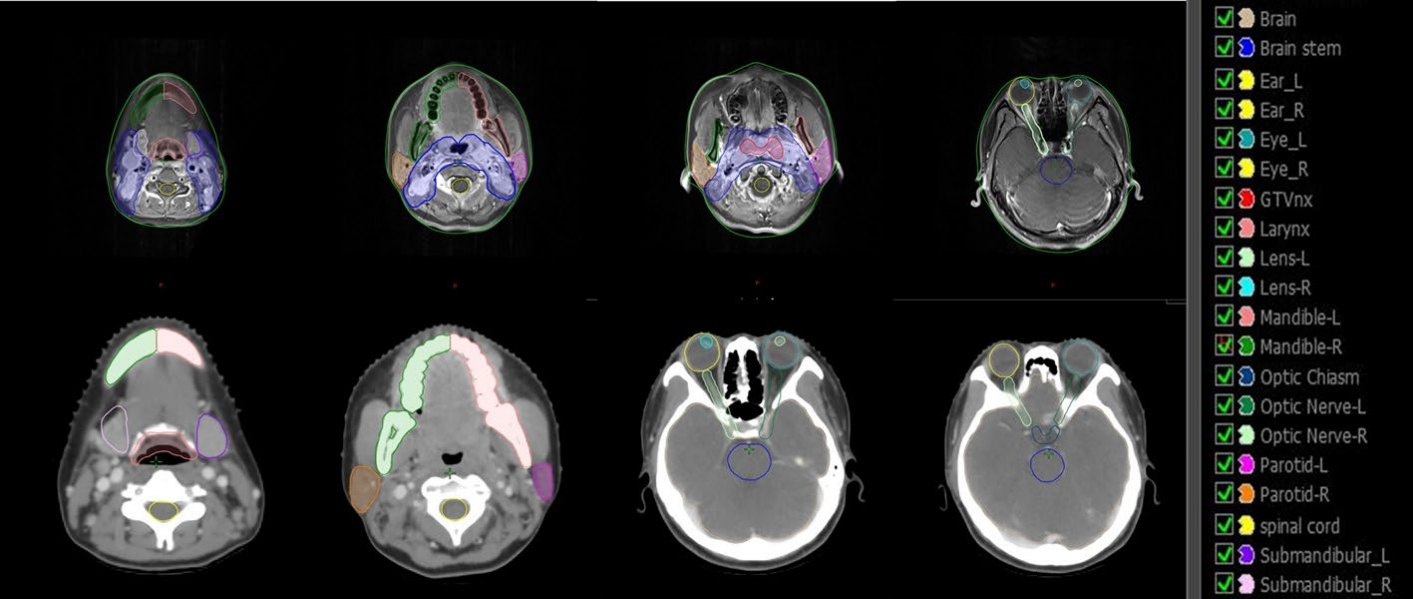
Sample CT/MRI image slices with OARs contours

**Fig. 2:**
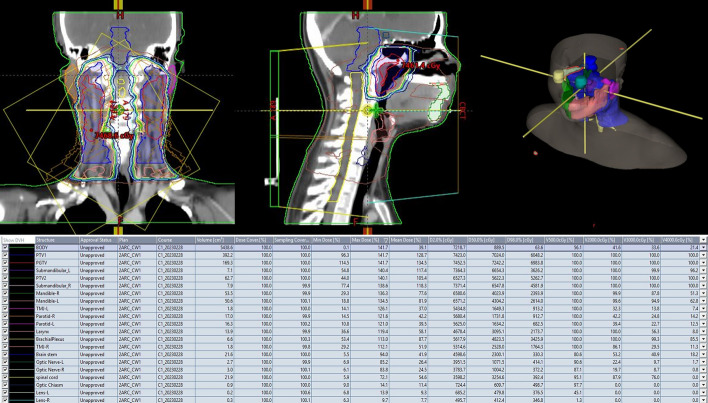
Radiotherapy plans for head and neck cancer and OARs

Accurate contouring of head and neck OARs has become a challenge for clinicians with the advent of precision radiotherapy. Presently, manual contouring of OARs is burdened with two challenges: reduced accuracy and increased time cost. Meanwhile, it has been demonstrated [13–16] that contouring of the target area varies among clinicians with different levels of experience, even for the same case. This could be due to the low pixel contrast on CT or MRI images and the clinicians' comprehension of the target area. Over-segmentation of OARs will make it difficult to optimize radiotherapy dose, while under-segmentation will subject OARs to an excessively high radiation dose, leading to irreversible side-effects on the patient's body (Table 1). The effectiveness of radiotherapy for cancer patients is seriously dependent on how accurately the OARs are contoured. The contouring of OARs is a labor-intensive task, and clinicians need to contour the cancerous foci and OARs layer by layer based on CT or MRI images (Figs. 1, 2), which will consume a lot of time [17–19]. With the development of deep learning technology, doctors have made great progress in contouring the target area, reducing radiological damage to patients, and even evaluating and improving patient prognosis [20–22].

**Table 1:** Dose limits and complication probability of head and neck radiotherapy OARs

OARs	Max dose	Possibility of toxicity	Most serious complication
Brain stem	54 Gy	< 5%	Neuropathy or necrosis
Spinal cord	50 Gy	0.2%	Radiological spinal cord injury
Mandible	60–65 Gy	< 5%	Radioactive osteonecrosis
Optic nerve/optic chiasm	< 55 Gy	< 3%	Nerve damage
Eyes (single)	< 35 Gy	–	Blindness
Parotid gland (single)	< 20 Gy	< 20%	Parotid function chronically less than 25%
Submandibular gland	< 35 Gy	–	Xerostomia

Despite the fact that there is a relatively large body of research literature focusing on this subject, there is still a lack of comprehensive review and meta-analysis of this area. The purpose of this meta-analysis is to review, summaries and analyses the performance of the DL technique for segmenting OARs in the head and neck region. The good image recognition and segmentation performance shown by the DL algorithm is promising. This study will focus on the following issues: the current status of DL algorithm segmentation in head and neck OARs, the influence of image modality and image type on the segmentation performance of the DL algorithm, and a systematic review of the key issues affecting the DL algorithm performance in the contouring of head and neck OARs and future development directions.

## Methods

### Search strategy

This single-arm meta-analysis is conducted based on the guidelines of the Preferred Reporting Items for Systematic Reviews and Meta-Analyses (PRISMA) [23]. We searched the literature in Pubmed, Embase, and Cochrane Library up to November 14, 2022, using the form of MeSH Terms + Entry Terms to search relevant literature. The search strategy is (Deep Learning OR Neural Networks) AND (Segmentation) AND (Head and Neck Neoplasms) AND (Organs at Risk). The detailed search strategy can be found in Additional file 1: Table S1.

### Selection criteria and data extraction

Studies with detailed OAR segmentation data, or studies able to calculate DSCs and their 95% confidence intervals (CIs) from other available data, are eligible. Studies with the following characteristics should be excluded: 1. studies on non-human species; 2. non-algorithmic studies, or contouring using mature segmentation software; 3. conference abstracts, reviews, book chapters, meta-analyses, editorials, duplicate literature; 4. non-English language studies; 5. lack of data; 6. unavailable literature; and 7. irrelevant studies.

Before data extraction, this study designed a data extraction form in conjunction with existing studies that will focus on the following data: 1. first author and year of publication; 2. country of first author attribution; 3. single-center or multicenter study; 4. prospective or retrospective study; 5. algorithm name; 6. image modality; 7. image type; 8. total number of patients; 9. test set sample size; and 10. head and neck OARs and corresponding DSC values and CI or standard deviations (SD).

### Quality assessment and risk of bias

Accurately described detail of the development and validation of clinical prediction models is necessary to adequately assess the generalizability of specific studies. Therefore, the *Checklist for Artificial Intelligence in Medical Imaging* (CLAIM) for DL [24] is chosen as the standard for assessing the quality of the literature, see Additional file 1: Table S2 for materials related to the CLAIM criteria.

For risk of bias, the *Prediction Model Risk of Bias Assessment Tool* (PROBAST), which focuses on methodological evaluation, is selected [25], and PROBAST is a risk of bias assessment tool for predictive model studies published by the Cochrane Assist Group in 2019. Moreover, it has been revised to be more appropriate for DL studies and its related fields with reference to Frizzell et al. [26], see Additional file 1: Table S3 for materials related to the PROBAST criteria.

Quality assessment of the literature and risk of bias assessment is carried out by a single person, and in case of uncertainty about the results, the decision is discussed with a second person.

## Statistical analysis

DSC is a quantitative analysis metric for evaluating graphic similarity in the field of computer vision. To calculate DSC, the computer first discrete the pixel points on the image and set the weight of each pixel point to 1. AT ∪ GT represents the sum of the weights of artificial intelligence target (AT) and ground truth (GT), AT ∩ GT represents the weight sum of the overlapping parts in AT and GT. The DSC takes values between [0, 1]. The closer the DSC is to 1, the better the fit between the AT and the GT area. In general, a DSC greater than 0.80 is considered to be a high similarity, a DSC greater than 0.70 is considered to be a moderate similarity, and a DSC less than 0.70 is considered to be a similarity that needs to be improved:

$$DSC=\frac{2\left(AT\cap GT\right)}{AT\cup GT}$$

The pooled effect size calculations, funnel plots and Egger's test for publication bias in this study were all done using Stata17. The calculation of the pooled effect size is based on the mean (mean) and 95% CI. For studies that did not report 95% CI data, reference is made to the methods in the *Cochrane handbook for systematic reviews of intervention*, using the test set sample size (*n*), the DSC mean (mean), the DSC SD were used to transform the data.

Higgins I^2^ is used to test for heterogeneity between studies, with *I*^2^ < 25% considered to have no heterogeneity, 25% ≤ *I*^2^ < 50% considered to have low heterogeneity, 50% ≤ *I*^2^ < 75% considered to have moderate heterogeneity, and *I*^2^ ≥ 75% considered to have high heterogeneity. The study selected either a fixed-effects model or a random-effects model based on the value of heterogeneity in the included literature.

Data analysis for this study were all performed using GraphPad Prism 9, and Student *t* test is used for comparison between groups. *p* < 0.05 (*) is considered a statistically significant difference, and vice versa (ns).

## Results

### Study selection and characteristics

**Fig. 3:**
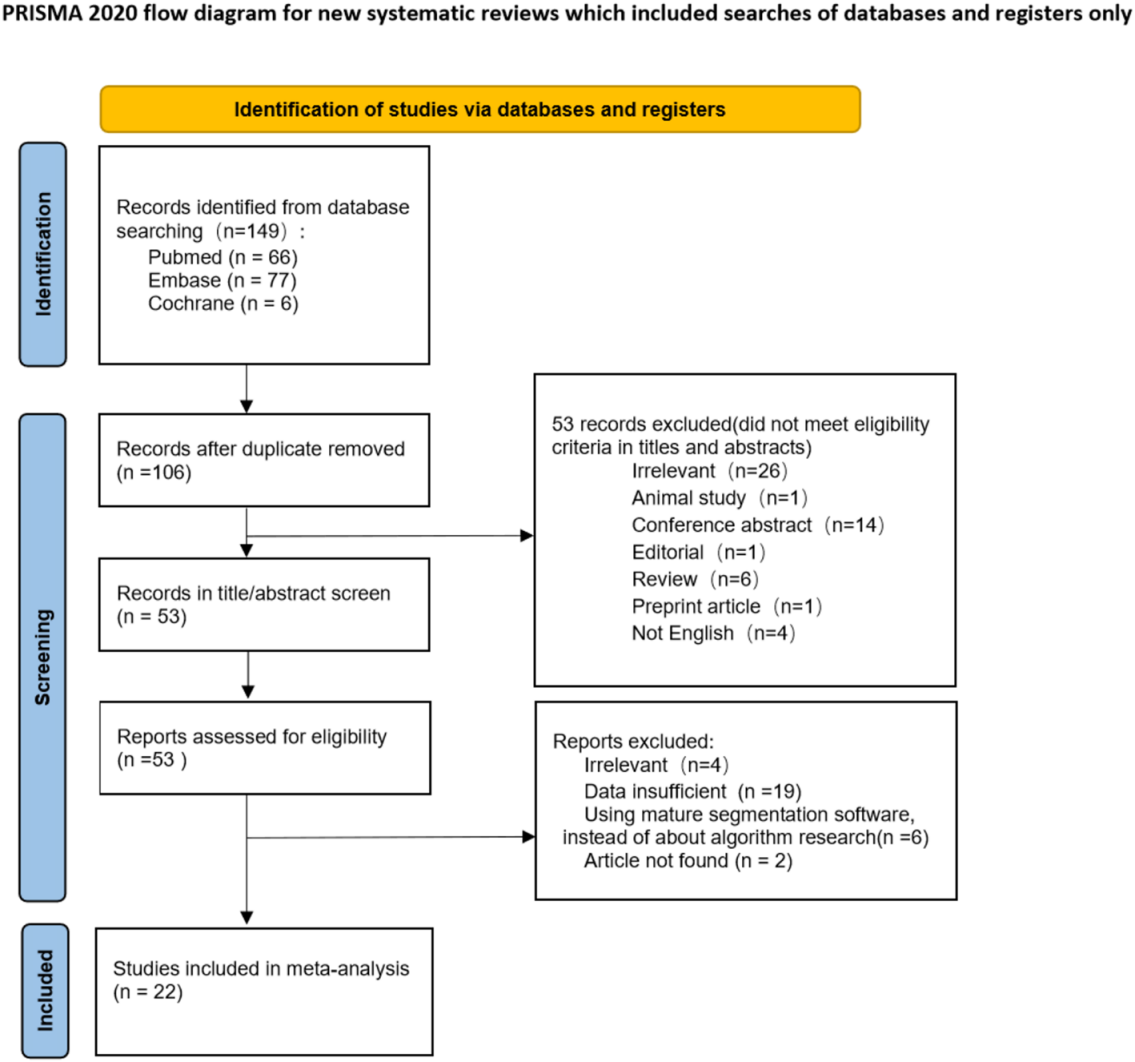
PRISMA flowchart of eligible studies selection process

With reference to the search strategy, a literature search is conducted in Pubmed, Embase and Cochrane Library for this study. 149 articles were retrieved and 106 articles were identified after excluding duplicates. After screening and detailed review and evaluation, a total of 22 articles were included in the meta-analysis (Fig. 3), involving 6,099 patients.

Among the 22 articles, 10 studies (45.45%) are from China, 5 studies (22.73%) are from the USA, 2 studies (9.09%) are from the Netherlands, 1 study (4.55%) is from Australia, 1 study (4.55%) is from the UK, 1 study (4.55%) is from Korea, 1 study (4.55%) is from Austria, and 1 study (4.55%) is from Austria. 2 studies (9.09%) are multicenter studies and 20 (90.91%) are single center studies. 18 studies (81.82%) perform contouring on CT images, 3 studies (13.64%) perform contouring on MRI images and 1 study (4.55%) on CT and MRI, respectively, and two DL models are trained. 5 studies (21.74%) use 2D images for contouring, 15 studies (65.22%) use 3D images for contouring, 1 study (4.55%) use 2.5D images for contouring and 2 studies (9.09%) do not specify the image type. 22 studies (100%) use internal validation sets to validate the algorithm performance and 8 studies (36.36%) use external validation sets. The detailed characteristics of the included literature can be found in Table 1 and the original data tables of the included literature can be found in Table 2.

**Table 2:** Characteristics of the included studies

Study ID	Country	Single center/Multicenter	Retrospective/Prospective research	Algorithm architecture name	Imaging modality	Imaging type	Internal validation	External validation	Amount of patient		
									Total	Dev	Test
Dai et al. (2021) [27]	America	Single center	Retrospective	MS–RCNN	MRI	3D	√	√	118	95	23
Tao et al. (2019) [28]	China	Single center	Retrospective	Boosting ResNets	CT	2D	√	–	140	120	20
Korte et al. (2021) [29]	Australia	Single center	Retrospective	–	MRI	–	√	–	41	31	10
Oktay et al. (2020) [17]	UK	Multicenter	Retrospective	Modified 3D U-Net	CT	3D	√	√	186	166	20
Ye et al. (2022) [18]	China	Multicenter	Retrospective	UaNet	CT	3D	√	√	502	176	326
Chan et al. (2019) [30]	America	Single center	Retrospective	LL-CNN	CT	3D	√	–	200	180	20
Chen et al. (2021) [23]	China	Single center	Retrospective	WBNet	CT	3D	√	√	180	150	30
Liang et al. (2019) [31]	China	Single center	Retrospective	ODS net	CT	2D	√	–	185	139	46
Kim et al. (2021) [32]	Korea	Single center	Retrospective	Modified FC-DenseNet	CT	2D	√	–	100	80	20
Gao et al. (2021) [33]	China	Single center	Retrospective	FocusNetv2	CT	3D	√	√	1164	1044	120
Nuo et al. (2018) [34]	China	Single center	Retrospective	FCNN + SRM	CT	3D	√	–	32	22	10
Fang et al. (2021) [35]	China	Single center	Retrospective	U-net	CT	2.5D	√	–	1000	800	200
V van Dijk et al. (2020) [36]	Netherlands	Single center	Retrospective	DLC	CT	3D	√	–	693	589	104
Dai et al. (2022) [37]	America	Single center	Retrospective	R-CNN	MRI	–	√	–	60	48	12
Tappeiner et al. (2019) [38]	Austria	Single center	Retrospective	HighRes3DNets	CT	3D	√	–	39	25	14
Gou et al. (2020) [39]	China	Single center	Retrospective	SCSA-Net	CT	3D	√	–	48	38	10
Nuo et al. (2019) [40]	China	Single center	Retrospective	SC-GAN-DenseNet	CT/MRI	3D	√	√	57	37	20
Zhang et al. (2021) [41]	China	Single center	Retrospective	–	CT	3D	√	–	170	150	20
Zhao et al. (2021) [42]	America	Single center	Retrospective	Weaving attention U-net	CT	3D	√	√	115	90	25
Liang et al. (2020) [43]	Not mentioned	Single center	Retrospective	–	CT	2D	√	√	48	33	15
Koo et al. (2022) [44]	America	Single center	Retrospective	–	CT	2D	√	–	864	–	–
Rooij et al. (2019) [20]	Netherlands	Single center	Retrospective	3D U-Net	CT	3D	√	–	157	142	15

### Results of the meta-analysis

There are many OARs in head and neck region, and DSC is selected as an effect size for meta-analysis of the results of four categories (12 in total) of organs at risk from each study. Central nervous system (CNS): brainstem, spinal cord. Bony structures: mandible. Visual organs: right and left optic nerve, right and left eye, optic chiasm. Glandular structures: right and left parotid glands, right and left submandibular glands. Pooled effect sizes, 95% CI can be found in Table 3. Forest plots for pooled effect size calculations can be found in Additional file 1: Fig. S1 (A–L).

**Table 3:** Raw data of the included studies

Study ID	Brain stem (± SD)	Spinal cord (± SD)	Mandible (± SD)	Optic nerve (Left) (± SD)	Optic nerve (Right) (± SD)	Eyes (Left) (± SD)	Eyes (Right) (± SD)	Optic chiasm (± SD)	Parotid gland (Left) (± SD)	Parotid gland (Right) (± SD)	Submandibular gland (Left) (± SD)	Submandibular gland (Right) (± SD)
Dai et al. (2021) [27]	0.93 ± 0.02	–	0.97 ± 0.01	0.8 ± 0.07	0.8 ± 0.08	–	–	0.73 ± 0.15	0.89 ± 0.05	0.88 ± 0.06	0.88 ± 0.05	0.85 ± 0.11
Tao et al. (2019) [28]	–	–	–	0.8994 ± 0.0431	0.8994 ± 0.0431	–	–	–	0.9188 ± 0.0351	0.9188 ± 0.0351	–	–
Korte et al. (2021) [29]	–	–	0.957 ± 0.023	–	–	–	–	–	0.86 ± 0.067	0.857 ± 0.063	0.83 ± 0.032	0.785 ± 0.123
Oktay et al. (2020) [17]	0.85 ± 0.037	0.84 ± 0.038	–	–	–	0.929 ± 0.016	0.931 ± 0.015	–	0.879 ± 0.038	0.878 ± 0.043	0.878 ± 0.043	0.875 ± 0.023
Ye et al. (2022) [18]	0.816 ± 0.053	0.815 ± 0.098	0.855 ± 0.135	0.676 ± 0.086	0.67 ± 0.097	0.0851 ± 0.132	0.862 ± 0.098	0.598 ± 0.158	0.832 ± 0.058	0.827 ± 0.062	0.792 ± 0.089	0.777 ± 0.092
Chan et al. (2019) [30]	0.89 ± 0.03	0.87 ± 0.03	0.91 ± 0.09	–	–	–	–	–	0.85 ± 0.03	0.86 ± 0.05	0.84 ± 0.01	0.85 ± 0.1
Chen et al. (2021) [23]	0.87 ± 0.03	–	0.94 ± 0.01	0.76 ± 0.06	0.75 ± 0.06	0.92 ± 0.03	0.93 ± 0.02	0.64 ± 0.15	0.85 ± 0.04	0.85 ± 0.04	0.82 ± 0.07	0.82 ± 0.08
Liang et al. (2019) [31]	0.896 ± 0.03	–	0.913 ± 0.035	0.661 ± 0.1	0.717 ± 0.1	0.932 ± 0.04	0.936 ± 0.03	–	0.852 ± 0.05	0.85 ± 0.05	–	–
Kim et al. (2021) [32]	0.87 ± 0.02	0.82 ± 0.04	0.95 ± 0.01	0.7 ± 0.07	0.72 ± 0.07	0.91 ± 0.02	0.91 ± 0.02	0.53 ± 0.16	0.84 ± 0.04	0.85 ± 0.04	0.83 ± 0.06	0.81 ± 0.1
Gao et al. (2021) [33]	0.8926 ± 0.0317	0.826 ± 0.0532	0.9211 ± 0.0155	0.6876 ± 0.0998	0.7332 ± 0.0884	0.8928 ± 0.0195	0.8895 ± 0.0234	0.6115 ± 0.1244	0.8505 ± 0.0547	0.8687 ± 0.0382	–	–
Nuo et al. (2018) [34]	0.8697 ± 0.0295	–	0.936 ± 0.0121	0.6531 ± 0.0575	0.6889 ± 0.0471	–	–	0.5835 ± 0.1028	0.08387 ± 0.0287	0.8346 ± 0.0234	0.767 ± 0.0731	0.8131 ± 0.0645
Fang et al. (2021) [35]	0.82 ± 0.062	0.796 ± 0.089	–	0.63 ± 0.145	0.638 ± 0.14	0.863 ± 0.076	0.86 ± 0.082	–	0.782 ± 0.093	0.785 ± 0.092	–	–
V van Dijk et al. (2020) [36]	0.84 ± 0.04	0.87 ± 0.06	0.94 ± 0.01	–	–	–	–	–	0.84 ± 0.04	0.83 ± 0.05	0.77 ± 0.12	0.78 ± 0.1
Dai et al. (2022) [37]	0.89 ± 0.06	0.77 ± 0.15	0.82 ± 0.1	0.67 ± 0.11	0.68 ± 0.11	0.89 ± 0.07	0.89 ± 0.05	0.61 ± 0.14	0.85 ± 0.06	0.86 ± 0.05	–	–
Tappeiner et al. (2019) [38]	0.82 ± 0.04	–	0.91 ± 0.02	0.64 ± 0.08	0.63 ± 0.06	–	–	0.42 ± 0.17	0.8 ± 0.1	0.81 ± 0.08	–	–
Gou et al. (2020) [39]	0.88 ± 0.02	–	0.94 ± 0.01	0.72 ± 0.05	0.71 ± 0.05	–	–	0.61 ± 0.06	0.87 ± 0.03	0.86 ± 0.05	0.78 ± 0.06	0.81 ± 0.07
Nuo et al. (2019) (model 1) [40]	0.8672 ± 0.029	–	0.9391 ± 0.013	0.6638 ± 0.048	0.6991 ± 0.043	–	–	0.5916 ± 0.097	0.8549 ± 0.017	0.8577 ± 0.024	0.8065 ± 0.05	0.8186 ± 0.049
Nuo et al. (2019) (model 2) [40]	0.9157 ± 0.0285	–	0.8164 ± 0.0444	0.7165 ± 0.0446	0.9631 ± 0.0658	–	–	0.5892 ± 0.0722	0.8648 ± 0.0501	0.8248 ± 0.0534	–	–
Zhang et al. (2021) [41]	0.87 ± 0.04	–	0.89 ± 0.02	0.73 ± 0.09	0.72 ± 0.09	0.89 ± 0.03	0.88 ± 0.03		0.71 ± 0.12	0.77 ± 0.07	–	–
Zhao et al. (2021) [42]	0.92 ± 0.03	0.89 ± 0.04	0.95 ± 0.04	0.76 ± 0.07	0.78 ± 0.09	–	–	0.74 ± 0.18	0.86 ± 0.06	0.88 ± 0.05	0.87 ± 0.07	0.85 ± 0.09
Liang et al. (2020) [43]	0.923 ± 0.1	–	0.941 ± 0.07	0.738 ± 0.046	0.734 ± 0.051	–	–	0.713 ± 0.083	0.882 ± 0.013	0.87 ± 0.015	0.815 ± 0.029	0.8 ± 0.034
Koo et al. (2022) [44]	0.88 ± 0.02	0.81 ± 0.07	0.87 ± 0.04	–	–	–	–	–	0.83 ± 0.11	0.83 ± 0.07	0.81 ± 0.1	0.83 ± 0.06
Rooij et al. (2019) [20]	0.64 ± 0.16	–	–	–	–	–	–	–	0.83 ± 0.02	0.82 ± 0.02	0.82 ± 0.07	0.81 ± 0.13

### CNS organs

Brainstem: A total of 21 models from 20 studies presented results for the brainstem, with a pooled DSC effect size of 0.87 (95% CI 0.85–0.89) and a Higgins *I*^2^ = 98.1%.

Spinal cord: A total of 10 models from 10 studies presented results for the spinal cord, with a pooled DSC effect size of 0.83 (95% CI 0.81–0.85) and a Higgins *I*^2^ = 95.2%.

### Bony structures

Mandible: A total of 19 models from 18 studies presented results for the spinal cord, with a pooled DSC effect size of 0.92 (95% CI 0.91–0.93) and a Higgins *I*^2^ = 98.5%.

### Visual organs

Eye: A total of 9 models from 9 studies presented results for the left and right eyes, with pooled DSC effect sizes of 0.90 (95% CI 0.88–0.91) and Higgins *I*^2^ = 96.4% for the left eye and 0.90 (95% CI 0.88–0.92) and Higgins *I*^2^ = 97.7% for the right eye, respectively.

Optic nerve: A total of 17 models from 16 studies presented results for the left and right optic nerve, with pooled DSC effect sizes of 0.71 (95% CI 0.68–0.75), Higgins *I*^2^ = 97.4% for the left optic nerve and 0.74 (95% CI 0.70–0.78), Higgins *I*^2^ = 97.8% for the right optic nerve, respectively.

Optic chiasm: A total of 13 models from 12 studies presented results for optic chiasm, with a pooled DSC effect size of 0.62 (95% CI 0.59–0.65) and Higgins *I*^2^ = 84.7%.

### Glandular structures

Parotid glands: A total of 23 models from 22 studies presented results for the left and right parotid glands, with pooled DSC effect sizes of 0.85 (95% CI 0.84–0.86) and Higgins *I*^2^ = 94.5% for the left parotid gland and 0.85 (95% CI 0.83–0.86) and Higgins *I*^2^ = 94.4% for the right parotid gland, respectively.

Submandibular glands: A total of 15 models from 15 studies presented results for the left and right submandibular glands with combined DSC effect sizes of 0.82 (95% CI 0.81–0.84), Higgins *I*^2^ = 92.4% for the left submandibular gland and 0.82 (95% CI 0.80–0.94) for the right submandibular gland with Higgins *I*^2^ = 93.5%.

### Publication bias

Publication bias is evaluated qualitatively using funnel plots and quantitatively using the Egger test. The funnel plot for the bias analysis can be found in Additional file 1: Fig. S2 (A–L). No publication bias is detected in the Egger test for the four categories of organs (*p* > 0.05), see Table 3 for the results.

### Subgroup analysis: comparison of contours on CT and MRI images

**Fig. 4:**
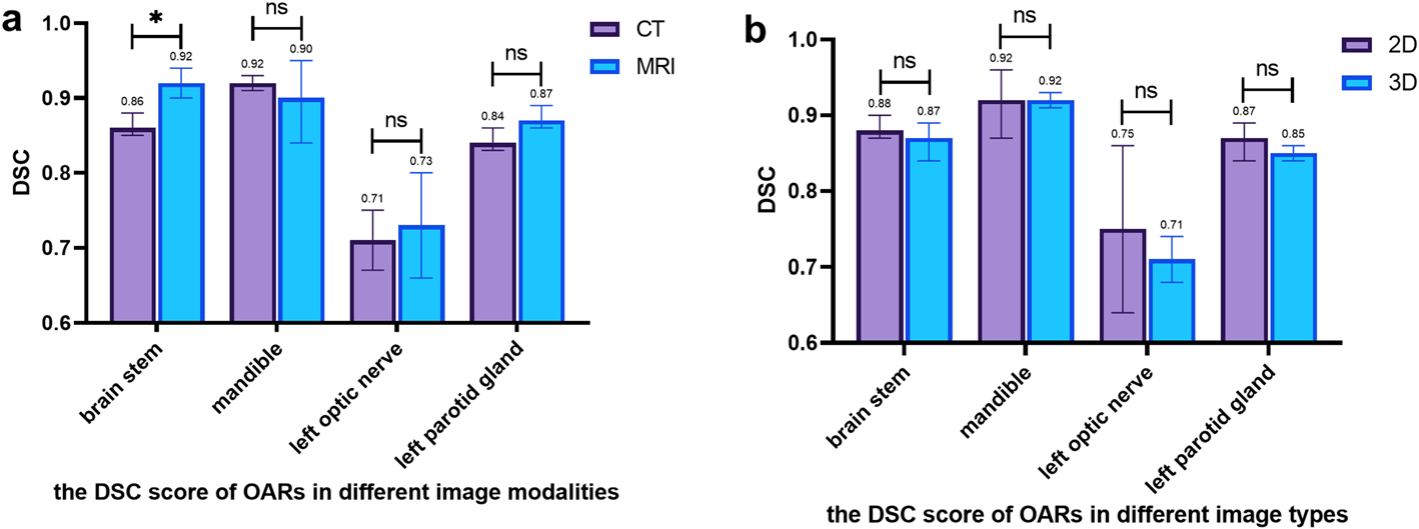
Bar chart of OARs DSC score in head and neck cancer patients of different image modalities and different image types

In this study, four representative organs (brainstem, mandible, left optic nerve, left parotid gland) were selected among the four types of OARs for study [Additional file 1: Fig. S3 (A–H)]. For the DL segmentation performance of DL on CT and MRI, the pooled effect sizes for the four types of organs in the studies using CT images for segmentation were 0.86 (95% CI 0.85–0.88), 0.92 (95% CI 0.91–0.93), 0.71 (95% CI 0.67–0.75), 0.84 (95% CI 0.83–0.86). The pooled effect sizes for the four types of organs in studies using MRI images for segmentation were 0.92 (95% CI 0.90–0.94), 0.90 (95% CI 0.84–0.95), 0.73 (95% CI 0.66–0.80), and 0.87 (95% CI 0.86–0.89), respectively. Among the organs’ contours in the two types of image modalities, the difference in brainstem is statistically significant (*p* = 0.0139), suggesting that DL is able to better contour the brainstem on MRI images. The segmentation result of the mandible, left optic nerve and left parotid gland is somewhat different (Fig. 4A) but did not show a statistically significant difference between the two modalities (*p* > 0.05).

### Subgroup analysis: comparison of contours on 2D and 3D modalities

This study also investigated the performance of DL in contouring the four organs mentioned above in different image types [Additional file 1: Fig. S4 (A–H)]. For DL segmentation performance on 2D and 3D modalities, the pooled effect sizes for the four types of organs in the study using 2D modalities for segmentation were 0.88 (95% CI 0.87–0.90), 0.92 (95% CI 0.87–0.96), 0.75 (95% CI 0.64–0.86), 0.87 (95% CI 0.84–0.89). The pooled effect sizes for the four types of organs in studies using 3D modalities for segmentation were: 0.87 (95% CI 0.84–0.89), 0.92 (95% CI 0.91–0.93), 0.71 (95% CI 0.68–0.74), 0.85 (95% CI 0.84–0.86). DL contours the brainstem, left optic nerve and left parotid gland better on 2D images than on 3D images (Fig. 4B). The mandible had the same results on both types of images. All four types of organs did not show a statistical difference (*p* > 0.05) between the two types of images, Table 4.

**Table 4:** Pooled dice similarity coefficient and Egger test of the publication bias of DL segmentation model

	References	Pooled estimate	95% CI	*I*^2^	Egger's bias	Egger's 95% CI	Egger's *P* value
Brain stem	[17, 18, 20, 23, 27, 30–44]	0.87	0.855 to 0.885	98.10%	− 0.363099	− 6.434718 to 5.70852	0.902
Spinal cord	[17, 18, 30, 32, 33, 35–37]	0.83	0.815 to 0.855	95.20%	1.172798	− 9.669338 to 12.01493	0.809
Mandible	[18, 23, 27, 29–34, 36, 37, 39–44]	0.92	0.907 to 0.930	98.50%	− 4.291492	− 10.1736 to 1.590612	0.142
Eye (Left)	[17, 18, 23, 31–33, 35, 37, 41]	0.90	0.882 to 0.914	96.40%	0.3947261	− 7.959603 to 8.749055	0.914
Eye (Right)	[17, 18, 23, 31–33, 35, 37, 41]	0.90	0.881 to 0.917	97.70%	− 1.326596	− 14.50229 to 11.8491	0.819
Optic nerve (Left)	[18, 23, 27, 28, 31–35, 37–43]	0.71	0.677 to 0.748	97.40%	1.666572	− 5.986573 to 9.319716	0.649
Optic nerve (Right)	[18, 23, 27, 28, 31–35, 37–43]	0.74	0.698 to 0.776	97.80%	4.376849	− 4.080193 to 12.83389	0.287
Optic chiasm	[18, 23, 27, 32–34, 37–40, 42, 43]	0.62	0.586 to 0.648	84.70%	0.4959904	− 2.725629 to 3.717609	0.741
Parotid gland (Left)	[17, 18, 20, 23, 27–44]	0.85	0.836 to 0.860	94.50%	− 0.3678269	− 4.289085 to 3.553431	0.847
Parotid gland (Right)	[17, 18, 20, 23, 27–44]	0.85	0.835 to 0.859	94.40%	0.1090494	− 3.900165 to 4.118264	0.955
Submandibular gland (Left)	[17, 18, 20, 23, 27, 29, 30, 32, 34, 36, 39, 40, 42–44]	0.82	0.806 to 0.839	92.40%	− 1.411071	− 4.497547 to 1.675404	0.341
Submandibular gland (Right)	[17, 18, 20, 23, 27, 29, 30, 32, 34, 36, 39, 40, 42–44]	0.82	0.796 to 0.843	93.50%	− 0.2339747	− 4.336639 to 3.868689	0.904

### Quality assessment and risk of bias

**Fig. 5:**
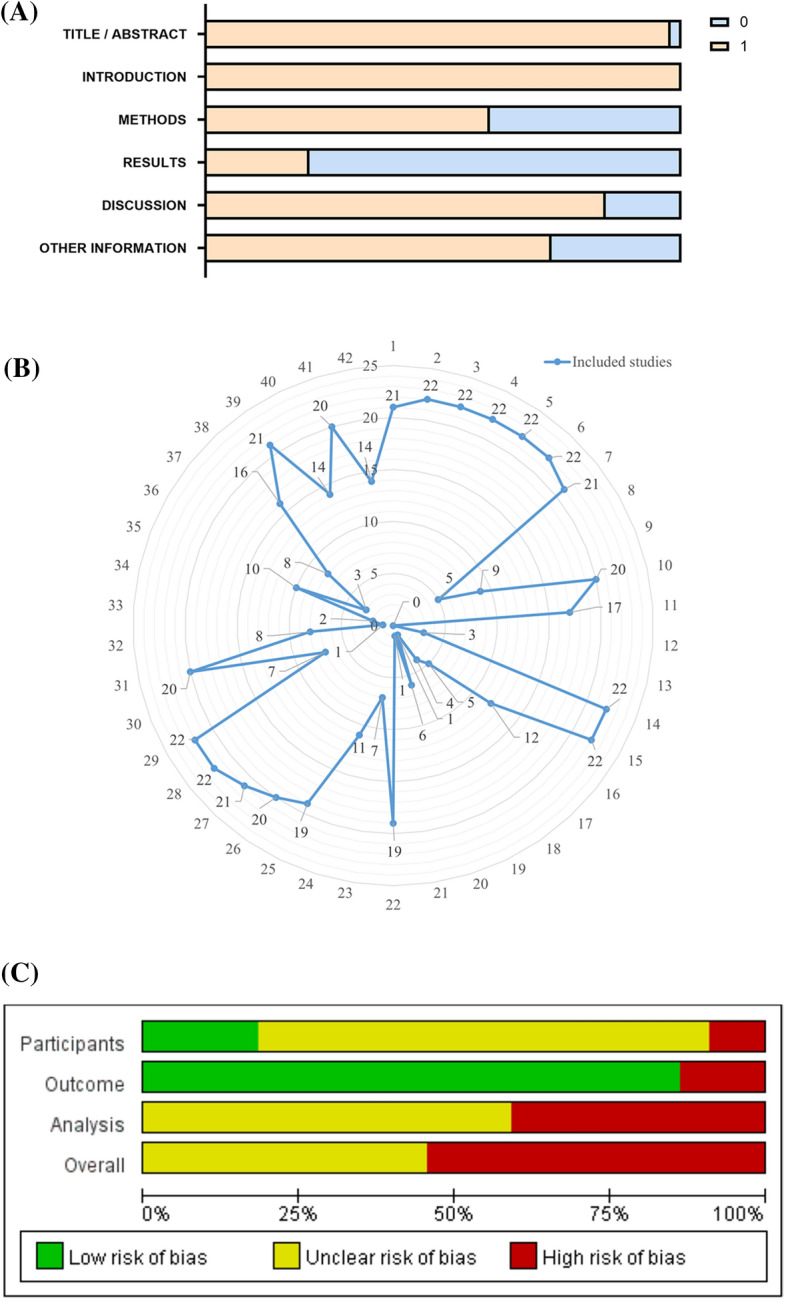

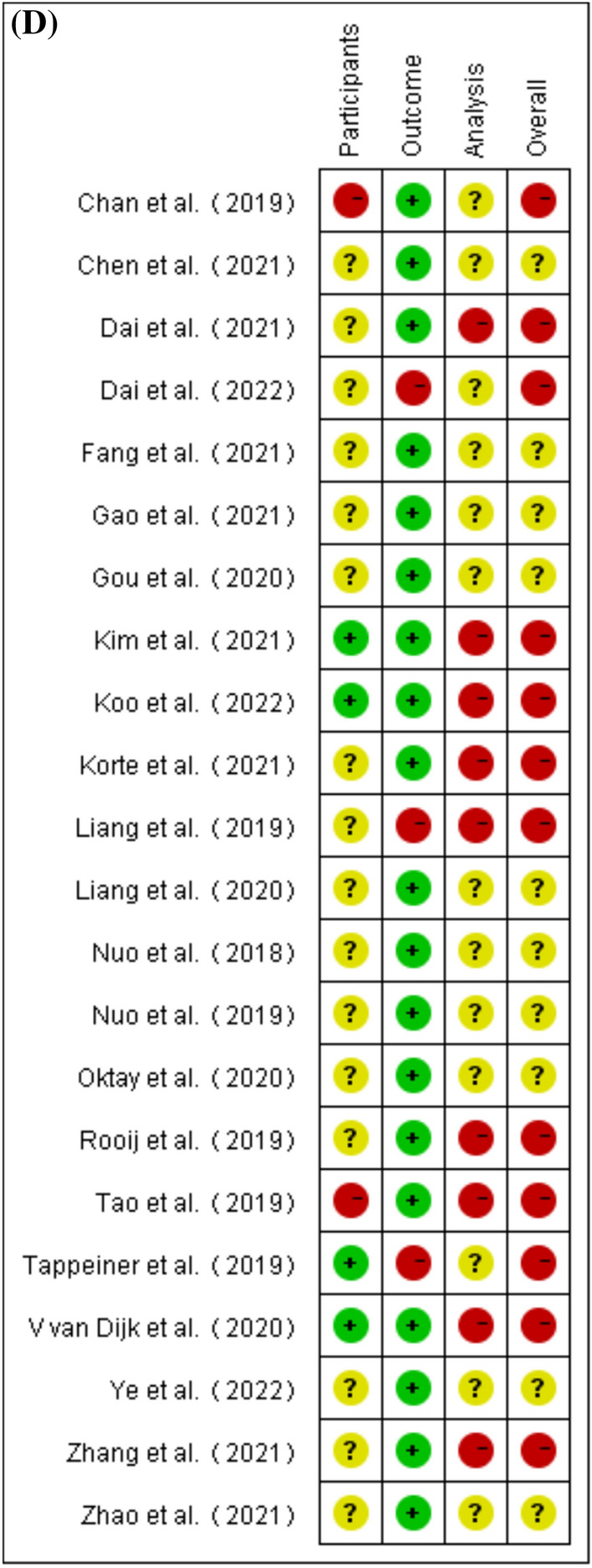
**A** Summary of CLAIM assessments of included studies. **B** Number of included studies meeting each CLAIM criterion. **C **Risk of bias graph according to PROBAST. **D **Risk of bias summary according to PROBAST

The six sections of the CLAIM criteria are presented as percentages in Fig. 5A. In the title/abstract section, 97.7% of the studies clearly and accurately described the type of artificial intelligence (AI), study design protocol, etc. 2.3% of the studies do not clearly specify these elements. In the "Introduction" section, all studies (100%) have described the disciplinary background, research objectives and research hypotheses. In the "Methods" section, 59.7% of the studies accurately provide detailed descriptions of the AI architecture, data sources, and training process, while 40.3% of the studies do not provide detailed descriptions of the data sources, pre-processing steps, or how to handle missing data. In the "Results" section, 78.2% of the studies are unclear about the inclusion/exclusion criteria for researchers, simply state the source of the CT/MRI images of the included patients, or lack an accurate assessment of the performance of the model, do not analyze cases that are incorrectly contoured. In the "Discussion" section, 84.1% of the studies comment on the limitations of this study, while 15.9% omit this element. For other information, 72.7% of the studies indicate information, such as the location, where the full study protocol could be accessed. Compliance with the CLAIM criteria for the 22 studies include in the meta-analysis ranged from 50% to 71.4%, with a mean of 61.0%. The number of studies meeting the 42 criteria in the CLAIM criteria can be found in Fig. 5B. Detailed results for the CLAIM criteria can be found in Additional file 1: Table S4.

About half (54.5%) of the studies show a high risk of bias according to the PROBAST assessment, Fig. 5C, D. The main source of high risk of bias is that the analysis section did not provide an accurate and comprehensive assessment of DL, including failure to assess metrics, such as specificity and sensitivity or failure to report model over-fitting, under-fitting and solutions. The risk of bias is unclear in less than half (45.5%) of the studies, mainly because the inclusion/exclusion criteria for the cases included in the study are not detailed. Detailed results of PROBAST can be found in Additional file 1: Table S5.

## Discussion

### DL has the ability to produce high-precision contours of head and neck OARs automatically.

**Fig. 6:**
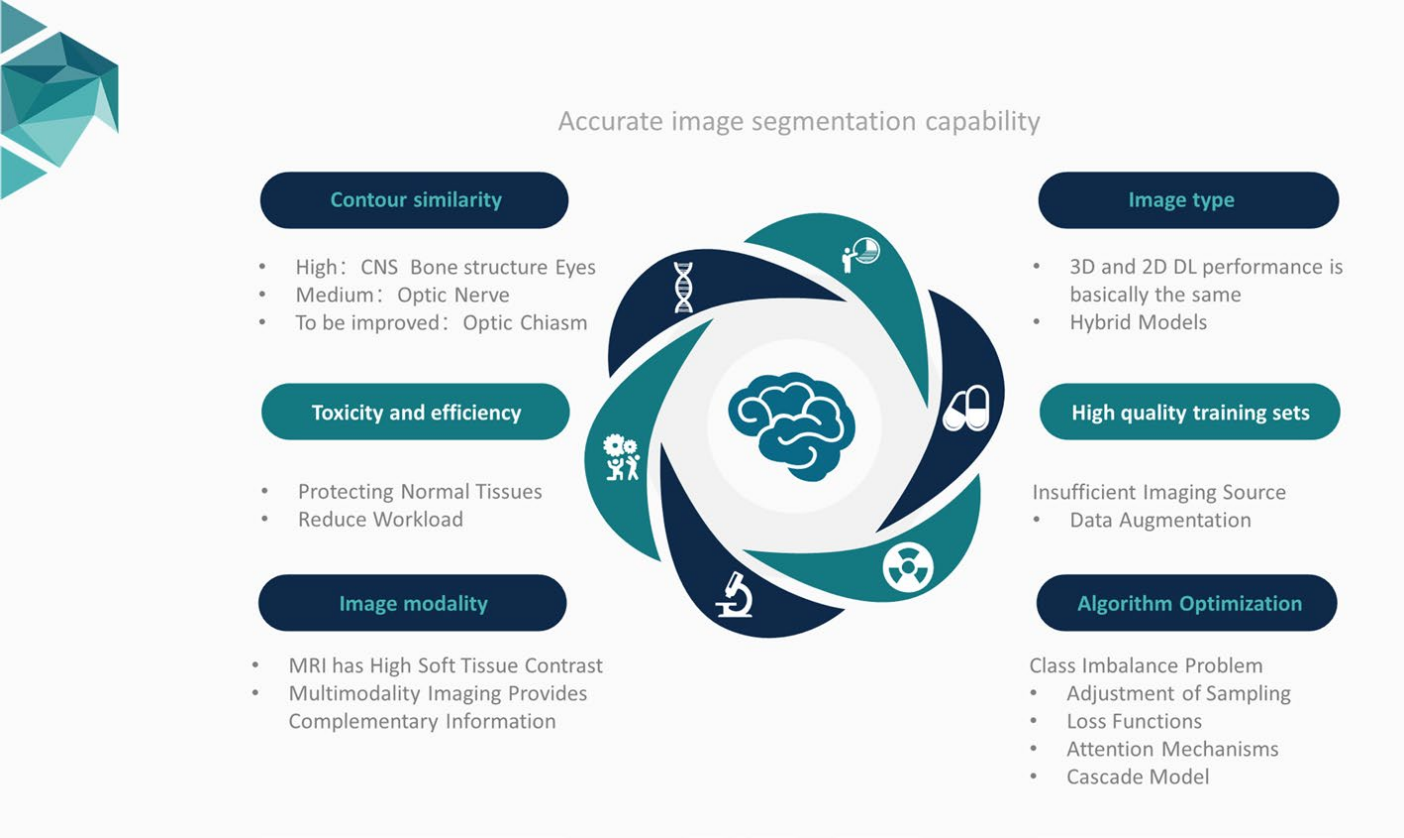
Contouring similarity and optimization directions for accurate image segmentation algorithms

In this study, it is found that DL has the capacity to generate highly precise contours in the automatic contouring of head and neck OARs. Overall, DL can attain a high level of similarity (DSC > 0.8) for CNS organs, bony structures, visual organs (eyes) and glandular structures, and a moderate level of similarity (DSC > 0.7) for the optic nerve in visual organs, while the ability to contour the optic chiasm needs to be improved (DSC < 0.7) (Fig. 6).

Radiation therapy for head and neck cancer is often associated with various radiotoxic reactions; these include optic nerve damage [27], cognitive deficits [45], and central nervous demyelinating lesions [46]. This requires the clinicians to strike a balance between maximizing the extent of tumor control and minimizing toxic effects, where even small differences in contouring may result in a difference in dose [47]. As the radiotherapy process progresses, the anatomy of head and neck region will change dramatically [48–50]. The location and shape of the tumor and surrounding OARs will change as a result of the exposure to the radiation. Moreover, the gradient of radiation dose distribution in image-guided radiotherapy changes drastically, and if no corresponding adjustments are made to the dose distribution according to the changes in the lesion and surrounding organs, damage to normal tissues will be exacerbated, while the tumor is not well-controlled [51].

### Image modality is an important factor affecting the performance of the DL algorithm, and multi-modality images can provide more accurate automatic contouring

DL's ability to segment CNS organs, visual organs and glandular structures on MRI images superior to that of similar algorithms on CT images, although this does not produce a statistical difference. This suggests that MRI is better equipped to segment soft tissue.

It is found that DL's ability to contour bony structures on MRI images is lessened in comparison with CT, which aligns with Tong et al.'s findings [40]. The bone cortex's low water content results in a low signal on MRI sequences, whereas bone tissue can strongly influence the ray beam attenuation on CT images, leading to a high-density signal. Due to the imaging limitations of single-modality images, DL faces challenges in extracting numerous imaging histological features from CT or MRI [27]. This severely restricts the accuracy of contouring head and neck OARs, which in turn, might have implicational effects on radiotherapy planning. Ibragimov et al. [52] also found that the convolutional neural network (CNN)-based DL algorithm is highly capable of identifying organs with clear boundaries on CT images, and for organs, such as optic chiasm, which are not well-defined, may require additional information to aid in contouring. Kieselmann et al. [53] are exploring the creation of sMRI image synthesized from CT by an algorithm based on generative adversarial networks. sMRI has the advantage of providing good complementary information on soft and bone tissues, and compared to image segmentation on CT alone, sMRI has a significant improvement in predicting optic chiasm, the cochlea and other organs [27, 54]. Multimodality images can provide additional imaging information for the accurate contouring of OARs.

### Image type is not a key factor in algorithm performance

The creation of 3D deep learning models necessitates numerous training parameters, leading to considerable computational overhead and potential overfitting hazards. [55, 56]. Due to hardware limitations, 3D DL model neural network depth is typically shallower than that of 2D DL models. This results in a reduced ability of 3D DL models to extract features and contour individual CT/MRI images, which explains why there is no significant difference in algorithm performance across image types. Furthermore, 2D DL models are fast, computationally efficient, and independent of layer thickness. [57, 58]. Medical images are often stored in 3D format in computers, and 3D DL models can efficiently utilize the correlation information from several contiguous images to provide more precise anatomical details and lesion features, therefore, overcoming the deficiency of information amongst body layers that is present in 2D DL models [27]. In general, 3D DL models produce more uniform, intricate, and lifelike contour of OARs. These models are capable of accurately modeling organs that have relatively stable anatomical positions [43]. 3D DL models have many advantages that are currently the focus of attention in the field of image segmentation. However, it is clear that 3D DL models do not currently demonstrate superior performance to 2D DL models.

To enhance the accuracy of segmentation for OARs with respect to image type, Fang et al. [35] applied a 2.5D U-Net model for OARs segmentation. For central slice information prediction, 2.5D images also entail the use of adjacent slices as input, even though the convolution kernel remains in 2D. 2.5D DL models enable the extraction of surrounding 3D information, while also reducing computational complexity, making them more efficient than traditional 3D CNNs [59]. Nuo et al. integrated shape representation models into 3D DL networks to predict images, along with a priori OAR shape features. [40]. In addition to single image types, there is ongoing research on hybrid 2D–3D CNN models. Various studies have implemented 2D–3D hybrid neural networks for organ segmentation [60–62], combining the semantic information of single slices extracted by 2D methods and the contextual semantic information extracted by 3D methods to achieve better segmentation results. Lee et al. [63] incorporated migration learning into organ segmentation. All of these schemes offer potential research ideas for accurately segmenting head and neck OARs. It is essential to emphasize that achieving accurate segmentation requires adequate pre-processing of medical images, irrespective of whether the algorithm segmentation performance is enhanced by the image modality or type. Operations such as removing artifacts, normalizing data, and aligning images can reduce the likelihood of inaccurate segmentation and facilitate image analysis. [64].

### Building a high-quality training set and enhancing innovation in the optimization of the algorithm are developing directions to further improve the performance of the algorithm

High-quality training data are a prerequisite for DL algorithms to achieve accurate predictions [40, 44]. A high-quality training data set is often a simpler and more effective means of enhancing of DL algorithms than a low-quality yet high-volume training data set [18]. Although DL models are robust to the noise of image data labels, Rolnick D's study showed a significant negative correlation between the amount of noise and the performance of automatic segmentation algorithms [65]. High-quality data sets are expensive to create, requiring a clinician's medical background, a significant amount of time and effort, among other factors. To overcome the challenge of limited access to such data sets, data augmentation has emerged as a potential solution. This involves generating variations of the original image by rotating, panning, cropping, and applying other techniques such as grayscale perturbation, scaling, and stretching to enhance diversity in the training data set. [34, 53]. Edward [66] performed data augmentation using limited data and evaluated the segmentation effect of a custom model (3D CNN) on a small data set, and the algorithm yielded an average surface distance of only 0.81 mm for the brainstem. Zhao et al. [67] used a principal component analysis model to randomly deform the original CT image to produce new data, and data augmentation provided small-sample-high-quality variants of the contours for DL. Asma Amjad et al. [68] used the adaptive spatial resolution method to improve the problem of low default spatial resolution (2 × 2 × 2 mm^3^) for identifying small organs, and a higher resolution of (1 × 1 × 2 mm^3^) for tissues, such as the optic nerve. Results on the test set showed that the DL algorithm contoured improved DSC values for all nine OARs, including the brainstem, inner ear and optic nerve.

The class imbalance problem is a major obstacle to computer image segmentation. It leads to a bias toward larger objects at the expense of smaller ones, resulting in higher rates of false positives and increased computational demands [41]. Currently, there are four main solutions for addressing the class imbalance problem: adjusting sampling methods, developing a new type of loss function, utilizing attention mechanisms, and implementing cascade models. Sampling method adjustments typically involve undersampling and oversampling techniques [69]. Undersampling adjusts the imbalance of categories by reducing the majority class samples, but may lead to information loss. On the other hand, oversampling methods can be used to expand imbalanced data, such as random oversampling, SMOTE oversampling [70], adaptive integrated oversampling [71], and random undersampling [72] which are popular sampling methods. The design of appropriate loss function is also one of the effective strategies to mitigate the impact of class imbalance, the advantage is that it will not destroy the original data distribution, the loss function mainly includes the loss function based on Dice, the loss function based on cross-entropy, or a combination of both. For example, researchers optimise the patch size of the segmentation architecture nnU-Net and use the class-adaptive Dice loss function to reduce the possibility of false positives brought by the image class imbalance problem [73], and Yeung et al. [74] combined dice- and cross-entropy-based loss to deal with the class imbalance, which reduces the loss to the class imbalance while converting voxel measurements to semantically labelled overlap measurements sensitivity to imbalance effects. The attention mechanism can selectively assign different weights to the input variables according to the importance differences, which can highlight useful information in image features while suppressing irrelevant information without the need for a large number of parameters and computational overheads. Ke Sheng et al. [39] designed a network architecture based on a spatial attention learning mechanism and a channel attention learning mechanism, which is able to priorities the invocation of neurons in regions potentially related to OARs, thus identifying meaningful features, which reduces the requirement of computer arithmetic and decreases the segmentation time. The fourth category of methods is cascade models, using cascade models can effectively take advantage of multiple models for image segmentation, e.g., James C. Korte et al. [29] used cascade CNNs to segment organs, such as submandibular gland and parotid gland of head and neck tumor patients, and still performed the image segmentation task using the original image resolution on a low dimensional image. In conclusion, the class imbalance problem is a key issue in DL-based segmentation of head and neck OARs, and future work on optimization at the level of data sources, algorithms, and hybrid models will help to improve global accuracy and reduce misclassification.

In conclusion, with the rapid development of computer vision and image processing technology, DL has immense potential for application in various fields, including healthcare, as well as image recognition and classification. This research paper assesses the performance of DL contouring OARs in head and neck region. Its excellent performance confirms the value of DL for clinical applications. However, there are also some urgent problems that need to be solved. For the future development of DL, it is necessary to strengthen theoretical research and innovation of algorithms while simultaneously building large medical image data sets. In addition, it is important to explore more intelligent, automated, and precise radiotherapy techniques.

This systematic review and meta-analysis analyzed the contouring performance of DL in contouring head and neck OARs for radiotherapy. There is some heterogeneity in the literature included in the study, which is an inherent limitation of single-arm meta-analysis. In addition, the low level of publication bias ensured the stability of the analysis results. The field of literature quality assessment and bias analysis of AI is highly controversial [75–79], the development of clinical prediction models necessitates comprehensive information to serve as a foundation to aid researchers in evaluating the models' performance and generalizability. The absence of adequate information to reiterate the model will heighten the risk of bias in articles, to a certain extent. In this paper, the assessment of article quality and analysis of bias did not yield very satisfactory results. This is due to the specific details reported in each research literature, resulting in a common lack of information among low quality/high bias studies. Therefore, this paper does not utilize study quality or risk of bias as a criterion for literature exclusion, but as an informative reference to aid researchers in carefully and objectively assessing high-level clinical evidence, rather than blindly utilizing it for clinical decision-making.

The limitations of this paper are as follows: 1. only the DSC metric is used to measure segmentation performance. Other parameters used in the field of computer vision to evaluate algorithm performance include mean surface distance, Hausdorff distance, Jaccard distance, and contouring time. The incorporation of additional, objective evaluation metrics would enhance the comprehensiveness of algorithm performance assessment. In addition, evaluating segmentation performance solely based on DSC does not fully indicate the effectiveness of response treatment [80], and in some studies it has been found that even large differences between DL contour and true contour do not necessarily affect the dosimetry or clinical feasibility of OARs [81]. Dose accuracy [22], normal tissue complication probability values [82] and the applicability of the target area to the clinic [36] are all subject to critical review by clinicians. 2. Diverse image sources: algorithm performance is closely tied to factors, such as imaging modality, device parameters, and characteristics of the patient population, each of which may directly affect the performance of the DL algorithm. Non-homogeneous parameter metrics may present a potential risk of bias.3. Assessing the interobserver contour variability of head and neck OARs and the impact of variability on the performance in DL algorithms has an important value in furthering the understanding and application of DL contours [44, 83], which will be one of the key elements of future research.

## Conclusion

The potential of DL is enormous, and it should be optimized and innovated in the future to coordinate with multiple institutions to create large-scale, multi-modality, high-quality medical data sets that integrate multiple information. DL is expected to become a powerful tool to promote the implementation of "precision radiotherapy" and provide individualized, standardized and refined treatment plans for patients.

### Supplementary Information

**Additional file 1: Table S1.** Search strategie**s. Table S2.** Checklist for Artificial Intelligence in Medical Imaging (CLAIM). **Table S3.** PROBAST (Prediction model Risk of Bias Assessment Tool) Review Items. Table **S4.** Result of CLAIM. **Table S5.** Result of PROBAST. **Figure S1 (A–L)** Forest plot of the pooled DSC of 12 OARs. **Figure S2 (A–L)** Funnel plots for meta-analysis of 12 OARs. **Figure S3 (A–H)** Forest plot of the DSC of segmentation of 4 OARs in CT or MRI images. **Figure S4 (A–H)** Forest plot of the DSC of segmentation of 4 OARs in 2D or 3D images.

## Data Availability

Not applicable.

## Abbreviations

OARs: Organs at risk
DL: Deep learning
DSC: Dice similarity coefficient
CLAIM: Checklist for Artificial Intelligence in Medical Imaging
PROBAST: Prediction Model Risk of Bias Assessment Tool
AI: Artificial intelligence
CNN: Convolutional neural network
