# Supplementary material for: Deep learning algorithm performance in contouring head and neck organs at risk: a systematic review and single-arm meta-analysis
Source: Biomed Eng Online. 2023 Nov 1;22:104. doi: 10.1186/s12938-023-01159-y (PMC10621161; doi:10.1186/s12938-023-01159-y)

Additional file

# Additional file 1: Table S1 Search strategies

# Additional file 1: Table S2 Checklist for Artificial Intelligence in Medical Imaging (CLAIM)

# Additional file 1: Table S3 PROBAST (Prediction model Risk of Bias Assessment Tool) Review Items

# Additional file 1: Table S4 Result of CLAIM

# Additional file 1: Table S5 Result of PROBAST

# Additional file 1: Figure S1(A-L) Forest plot of the pooled DSC of 12 OARs

# Additional file 1: Figure S2(A-L) Funnel plots for meta-analysis of 12 OARs

# Additional file 1: Figure S3(A-H) Forest plot of the DSC of segmentation of 4 OARs in CT or MRI images

# Additional file 1: Figure S4(A-H) Forest plot of the DSC of segmentation of 4 OARs in 2D or 3D images

**Additional file 1: Table S1** Search strategies

| **Search** | **PubMed Query – November 14, 2022** | **Items found** |
| --- | --- | --- |
| #7 | #3 AND #4 AND #5 AND #6 | 66 |
| #6 | (((("Organs at Risk"[Mesh]) OR (Risk, Organs at[Title/Abstract])) OR (Risks, Organs at[Title/Abstract])) OR (at Risk, Organs[Title/Abstract])) OR (at Risks, Organs[Title/Abstract]) | 28,986 |
| #5 | ((((((((((((((((((((((((((((((((("Head and Neck Neoplasms"[Mesh]) OR (Neoplasms, Head[Title/Abstract] AND Neck[Title/Abstract])) OR (Head, Neck Neoplasms[Title/Abstract])) OR (Head[Title/Abstract] AND Neck Neoplasm[Title/Abstract])) OR (Cancer of Head[Title/Abstract] AND Neck[Title/Abstract])) OR (Head[Title/Abstract] AND Neck Cancer[Title/Abstract])) OR (Cancer of the Head[Title/Abstract] AND Neck[Title/Abstract])) OR (Upper Aerodigestive Tract Neoplasms[Title/Abstract])) OR (UADT Neoplasm[Title/Abstract])) OR (Neoplasm, UADT[Title/Abstract])) OR (Neoplasms, UADT[Title/Abstract])) OR (UADT Neoplasms[Title/Abstract])) OR (Neoplasms, Upper Aerodigestive Tract[Title/Abstract])) OR (Upper Aerodigestive Tract Neoplasm[Title/Abstract])) OR (Head Neoplasms[Title/Abstract])) OR (Neoplasms, Head[Title/Abstract])) OR (Head Neoplasm[Title/Abstract])) OR (Neoplasm, Head[Title/Abstract])) OR (Neck Neoplasms[Title/Abstract])) OR (Neoplasms, Neck[Title/Abstract])) OR (Neck Neoplasm[Title/Abstract])) OR (Neoplasm, Neck[Title/Abstract])) OR (Cancer of Head[Title/Abstract])) OR (Head Cancers[Title/Abstract])) OR (Head Cancer[Title/Abstract])) OR (Cancer, Head[Title/Abstract])) OR (Cancers, Head[Title/Abstract])) OR (Cancer of the Head[Title/Abstract])) OR (Cancer of Neck[Title/Abstract])) OR (Neck Cancers[Title/Abstract])) OR (Neck Cancer[Title/Abstract])) OR (Cancer, Neck[Title/Abstract])) OR (Cancers, Neck[Title/Abstract])) OR (Cancer of the Neck[Title/Abstract]) | 353,119 |
| #4 | ((((segmentation[Title/Abstract]) OR (automated segmentation[Title/Abstract])) OR (classification[Title/Abstract])) OR (automated classification[Title/Abstract])) OR (auto delineate[Title/Abstract]) | 441,994 |
| #3 | #1 OR #2 | 614,449 |
| #2 | (((((((((((((((((((((((((("Neural Networks, Computer"[Mesh]) OR (Computer Neural Networks[Title/Abstract])) OR (Network, Computer Neural[Title/Abstract])) OR (Networks, Computer Neural[Title/Abstract])) OR (Neural Network, Computer[Title/Abstract])) OR (Perceptrons[Title/Abstract])) OR (Perceptron[Title/Abstract])) OR (Neural Network Models[Title/Abstract])) OR (Connectionist Models[Title/Abstract])) OR (Connectionist Model[Title/Abstract])) OR (Model, Connectionist[Title/Abstract])) OR (Models, Connectionist[Title/Abstract])) OR (Models, Neural Network[Title/Abstract])) OR (Model, Neural Network[Title/Abstract])) OR (Network Model, Neural[Title/Abstract])) OR (Network Models, Neural[Title/Abstract])) OR (Neural Network Model[Title/Abstract])) OR (Neural Networks (Computer[Title/Abstract]))) OR (Network, Neural (Computer[Title/Abstract]))) OR (Networks, Neural (Computer[Title/Abstract]))) OR (Neural Network (Computer[Title/Abstract]))) OR (Computational Neural Networks[Title/Abstract])) OR (Computational Neural Network[Title/Abstract])) OR (Network, Computational Neural[Title/Abstract])) OR (Networks, Computational Neural[Title/Abstract])) OR (Neural Network, Computational[Title/Abstract])) OR (Neural Networks, Computational[Title/Abstract]) | 56,411 |
| #1 | (((((("Deep Learning"[Mesh]) OR (Learning, Deep[Title/Abstract])) OR (Hierarchical Learning[Title/Abstract])) OR (Learning, Hierarchical[Title/Abstract])) OR (Deep learning[Title/Abstract])) OR (deep[Title/Abstract])) OR (learning[Title/Abstract]) | 589,760 |

| **Search** | **Cochrane library Query – November 14, 2022** | **Items found** |
| --- | --- | --- |
| #1 | MeSH descriptor: [Deep Learning] explode all trees | 71 |
| #2 | (Deep learning):ti,ab,kw OR (Hierarchical Learning):ti,ab,kw OR (Learning, Hierarchical):ti,ab,kw OR (Learning, Deep):ti,ab,kw | 1,367 |
| #3 | #1 OR #2 | 1,367 |
| #4 | MeSH descriptor: [Neural Networks, Computer] explode all trees | 207 |
| #5 | (Computer Neural Network):ti,ab,kw OR(Computer Neural Networks):ti,ab,kw OR(Network, Computer Neural):ti,ab,kw OR(Networks, Computer Neural):ti,ab,kw OR(Neural Network, Computer):ti,ab,kw OR(Perceptrons):ti,ab,kw OR(Perceptron):ti,ab,kw OR(Neural Network Models):ti,ab,kw OR(Connectionist Models):ti,ab,kw OR(Connectionist Model):ti,ab,kw OR(Model, Connectionist):ti,ab,kw OR(Models, Connectionist):ti,ab,kw OR(Models, Neural Network):ti,ab,kw OR(Model, Neural Network):ti,ab,kw OR(Network Model, Neural):ti,ab,kw OR(Network Models, Neural):ti,ab,kw OR(Neural Network Model):ti,ab,kw OR(Neural Networks (Computer) ):ti,ab,kw OR(Network, Neural (Computer) ):ti,ab,kw OR(Networks, Neural (Computer) ):ti,ab,kw OR(Neural Network (Computer) ):ti,ab,kw OR(Computational Neural Networks):ti,ab,kw OR(Computational Neural Network):ti,ab,kw OR(Network, Computational Neural):ti,ab,kw OR(Networks, Computational Neural):ti,ab,kw OR(Neural Network, Computational):ti,ab,kw OR(Neural Networks, Computational):ti,ab,kw | 1,179 |
| #6 | #4 OR #5 | 1,223 |
| #7 | #3 OR #6 | 1,223 |
| #8 | (segmentation):ti,ab,kw OR (automated segmentation):ti,ab,kw OR (classification):ti,ab,kw OR (automated classification):ti,ab,kw OR (auto delineate):ti,ab,kw | 52,378 |
| #9 | MeSH descriptor: [Head and Neck Neoplasms] explode all trees | 6,730 |
| #10 | (Neoplasms, Head and Neck):ti,ab,kw OR(Head, Neck Neoplasms):ti,ab,kw OR(Head and Neck Neoplasm):ti,ab,kw OR(Cancer of Head and Neck):ti,ab,kw OR(Head and Neck Cancer):ti,ab,kw OR(Cancer of the Head and Neck):ti,ab,kw OR(Upper Aerodigestive Tract Neoplasms):ti,ab,kw OR(UADT Neoplasm):ti,ab,kw OR(Neoplasm, UADT):ti,ab,kw OR(Neoplasms, UADT):ti,ab,kw OR(UADT Neoplasms):ti,ab,kw OR(Neoplasms, Upper Aerodigestive Tract):ti,ab,kw OR(Upper Aerodigestive Tract Neoplasm):ti,ab,kw OR(Head Neoplasms):ti,ab,kw OR(Neoplasms, Head):ti,ab,kw OR(Head Neoplasm):ti,ab,kw OR(Neoplasm, Head):ti,ab,kw OR(Neck Neoplasms):ti,ab,kw OR(Neoplasms, Neck):ti,ab,kw OR(Neck Neoplasm):ti,ab,kw OR(Neoplasm, Neck):ti,ab,kw OR(Cancer of Head):ti,ab,kw OR(Head Cancers):ti,ab,kw OR(Head Cancer):ti,ab,kw OR(Cancer, Head):ti,ab,kw OR(Cancers, Head):ti,ab,kw OR(Cancer of the Head):ti,ab,kw OR(Cancer of Neck):ti,ab,kw OR(Neck Cancers):ti,ab,kw OR(Neck Cancer):ti,ab,kw OR(Cancer, Neck):ti,ab,kw OR(Cancers, Neck):ti,ab,kw OR(Cancer of the Neck):ti,ab,kw | 10,448 |
| #11 | #9 OR #10 | 14,152 |
| #12 | MeSH descriptor: [Organs at Risk] explode all trees | 90 |
| #13 | (Organs at Risks):ti,ab,kw OR(Risk, Organs at):ti,ab,kw OR(Risks, Organs at):ti,ab,kw OR (at Risk, Organs):ti,ab,kw OR(at Risks, Organs):ti,ab,kw | 1,680 |
| #14 | #12 OR #13 | 1,680 |
| #15 | #7 AND #8 AND #11 AND #14 | 6 |

| **Search** | **Embase Query – October 19, 2022** | **Items found** |
| --- | --- | --- |
| #15 | #7 AND #8 AND #11 AND #14 | 77 |
| #14 | #12 OR #13 | 10,777 |
| #13 | 'organs at risks':ab,kw,ti OR 'risk, organs at':ab,kw,ti OR 'risks, organs at':ab,kw,ti OR 'at risk, organs':ab,kw,ti OR 'at risks, organs':ab,kw,ti | 361 |
| #12 | 'organs at risk'/exp | 10,638 |
| #11 | #9 OR #10 | 409,971 |
| #10 | 'neoplasms, head and neck':ab,kw,ti OR 'head, neck neoplasms':ab,kw,ti OR 'head and neck neoplasm':ab,kw,ti OR 'cancer of head and neck':ab,kw,ti OR 'head and neck cancer':ab,kw,ti OR 'cancer of the head and neck':ab,kw,ti OR 'upper aerodigestive tract neoplasms':ab,kw,ti OR 'uadt neoplasm':ab,kw,ti OR 'neoplasm, uadt':ab,kw,ti OR 'neoplasms, uadt':ab,kw,ti OR 'uadt neoplasms':ab,kw,ti OR 'neoplasms, upper aerodigestive tract':ab,kw,ti OR 'upper aerodigestive tract neoplasm':ab,kw,ti OR 'head neoplasms':ab,kw,ti OR 'neoplasms, head':ab,kw,ti OR 'head neoplasm':ab,kw,ti OR 'neoplasm, head':ab,kw,ti OR 'neck neoplasms':ab,kw,ti OR 'neoplasms, neck':ab,kw,ti OR 'neck neoplasm':ab,kw,ti OR 'neoplasm, neck':ab,kw,ti OR 'cancer of head':ab,kw,ti OR 'head cancers':ab,kw,ti OR 'head cancer':ab,kw,ti OR 'cancer, head':ab,kw,ti OR 'cancers, head':ab,kw,ti OR 'cancer of the head':ab,kw,ti OR 'cancer of neck':ab,kw,ti OR 'neck cancers':ab,kw,ti OR 'neck cancer':ab,kw,ti OR 'cancer, neck':ab,kw,ti OR 'cancers, neck':ab,kw,ti OR 'cancer of the neck':ab,kw,ti | 55,795 |
| #9 | 'head and neck tumor'/exp | 404,497 |
| #8 | 'segmentation':ab,kw,ti OR 'automated segmentation':ab,kw,ti OR 'classification':ab,kw,ti OR 'automated classification':ab,kw,ti OR 'auto delineate':ab,kw,ti | 610,518 |
| #7 | #3 OR #6 | 946,214 |
| #6 | #4 OR #5 | 81,854 |
| #5 | 'neural networkscomputer':ab,kw,ti OR 'computer neural network':ab,kw,ti OR 'network, computer neural':ab,kw,ti OR 'networks, computer neural':ab,kw,ti OR 'neural network, computer':ab,kw,ti OR 'perceptrons':ab,kw,ti OR 'perceptron':ab,kw,ti OR 'neural network models':ab,kw,ti OR 'connectionist models':ab,kw,ti OR 'connectionist model':ab,kw,ti OR 'model, connectionist':ab,kw,ti OR 'models, connectionist':ab,kw,ti OR 'models, neural network':ab,kw,ti OR 'model, neural network':ab,kw,ti OR 'network model, neural':ab,kw,ti OR 'network models, neural':ab,kw,ti OR 'neural network model':ab,kw,ti OR 'neural networks (computer)':ab,kw,ti OR 'network, neural (computer)':ab,kw,ti OR 'networks, neural (computer)':ab,kw,ti OR 'neural network (computer)':ab,kw,ti OR 'computational neural networks':ab,kw,ti OR 'computational neural network':ab,kw,ti OR 'network, computational neural':ab,kw,ti OR 'networks, computational neural':ab,kw,ti OR 'neural network, computational':ab,kw,ti OR 'neural networks, computationa':ab,kw,ti | 12,190 |
| #4 | 'artificial neural network'/exp | 77,136 |
| #3 | #1 OR #2 | 905,206 |
| #2 | 'learning, deep':ab,kw,ti OR 'hierarchical learning':ab,kw,ti OR 'learning, hierarchical':ab,kw,ti OR 'deep':ab,kw,ti OR 'learning':ab,kw,ti | 903,357 |
| #1 | 'deep learning'/exp | 310,28 |

**Additional file 1: Table S2** Checklist for Artificial Intelligence in Medical Imaging (CLAIM)

| Section / Topic | No. | Item |
| --- | --- | --- |
| TITLE / ABSTRACT |  |  |
|  | 1 | Identification as a study of AI methodology, specifying the category of technology used (e.g., deep learning) |
|  | 2 | Structured summary of study design, methods, results, and conclusions |
| INTRODUCTION |  |  |
|  | 3 | Scientific and clinical background, including the intended use and clinical role of the AI approach |
|  | 4 | Study objectives and hypotheses |
| METHODS |  |  |
| *Study Design* | 5 | Prospective or retrospective study |
|  | 6 | Study goal, such as model creation, exploratory study, feasibility study, non-inferiority trial |
| *Data* | 7 | Data sources |
|  | 8 | Eligibility criteria: how, where, and when potentially eligible participants or studies were identified (e.g., symptoms, results from previous tests, inclusion in registry, patient-care setting, location, dates) |
|  | 9 | Data pre-processing steps |
|  | 10 | Selection of data subsets, if applicable |
|  | 11 | Definitions of data elements, with references to Common Data Elements |
|  | 12 | De-identification methods |
|  | 13 | How missing data were handled |
| *Ground Truth* | 14 | Definition of ground truth reference standard, in sufficient detail to allow replication |
|  | 15 | Rationale for choosing the reference standard (if alternatives exist) |
|  | 16 | Source of ground-truth annotations; qualifications and preparation of annotators |
|  | 17 | Annotation tools |
|  | 18 | Measurement of inter- and intra-rater variability; methods to mitigate variability and/or resolve discrepancies |
| *Data Partitions* | 19 | Intended sample size and how it was determined |
|  | 20 | How data were assigned to partitions; specify proportions |
|  | 21 | Level at which partitions are disjoint (e.g., image, study, patient, institution) |
| *Model* | 22 | Detailed description of model, including inputs, outputs, all intermediate layers and connections |
|  | 23 | Software libraries, frameworks, and packages |
|  | 24 | Initialization of model parameters (e.g., randomization, transfer learning) |
| *Training* | 25 | Details of training approach, including data augmentation, hyperparameters, number of models trained |
|  | 26 | Method of selecting the final model |
|  | 27 | Ensembling techniques, if applicable |
| *Evaluation* | 28 | Metrics of model performance |
|  | 29 | Statistical measures of significance and uncertainty (e.g., confidence intervals) |
|  | 30 | Robustness or sensitivity analysis |
|  | 31 | Methods for explainability or interpretability (e.g., saliency maps), and how they were validated |
|  | 32 | Validation or testing on external data |
| RESULTS |  |  |
| *Data* | 33 | Flow of participants or cases, using a diagram to indicate inclusion and exclusion |
|  | 34 | Demographic and clinical characteristics of cases in each partition |
| *Model performance* | 35 | Performance metrics for optimal model(s) on all data partitions |
|  | 36 | Estimates of diagnostic accuracy and their precision (such as 95% confidence intervals) |
|  | 37 | Failure analysis of incorrectly classified cases |
| DISCUSSION |  |  |
|  | 38 | Study limitations, including potential bias, statistical uncertainty, and generalizability |
|  | 39 | Implications for practice, including the intended use and/or clinical role |
| OTHER INFORMATION |  |  |
|  | 40 | Registration number and name of registry |
|  | 41 | Where the full study protocol can be accessed |
|  | 42 | Sources of funding and other support; role of funders |

24. Mongan J, Moy L, Kahn CE, Jr. Checklist for Artificial Intelligence in Medical Imaging (Claim): A Guide for Authors and Reviewers. *Radiol Artif Intell* (2020) 2(2): e200029. Epub 20200325. doi: 10.1148/ryai.2020200029.

**Additional file 1: Table S3** PROBAST (Prediction model Risk of Bias Assessment Tool) Review Items

| **Domain** | **Signaling Question** |
| --- | --- |
| 1.Participants | 1.1 Were appropriate data sources used, e.g., cohort, or nested case-control study data? |
|  | 1.2 Were all inclusions and exclusions of participants appropriate? |
| 2. Predictors | 2.1 Were predictors defined and assessed in a similar way for all participants? |
|  | 2.2 Were predictor assessments made without knowledge of outcome data? |
|  | 2.3 Are all predictors available at the time the model is intended to be used? |
| 3. Outcome | 3.1 Was the outcome determined appropriately? |
|  | 3.2 Was a prespecified or standard outcome definition used? |
|  | 3.3 Were predictors excluded from the outcome definition? |
|  | 3.4 Was the outcome defined and determined in a similar way for all participants? |
|  | 3.5 Was the outcome determined without knowledge of predictor information? |
|  | 3.6 Was the time interval between predictor assessment and outcome determination appropriate? |
| 4. Analysis | 4.1 Were there a reasonable number of participants with the outcome? |
|  | 4.2 Were continuous and categorical predictors handled appropriately? |
|  | 4.3 Were all enrolled participants included in the analysis? |
|  | 4.4 Were participants with missing data handled appropriately? |
|  | 4.5 Was selection of predictors based on univariable analysis avoided? |
|  | 4.6 Were complexities in the data (e.g., censoring, competing risks, sampling of control participants accounted for appropriately? |
|  | 4.7 Were relevant model performance measures evaluated appropriately? |
|  | 4.8 Were model overfitting and optimism in model performance accounted for? |
|  | 4.9 Do predictors and their assigned weights in the final model correspond to the results from the reported multivariable analysis? |
| Applicability | Included participants or setting does not match the review question. |
|  | Definition, assessment, or timing of predictors does not match the review questions. |
|  | Its definition, timing, or determination does not match the review question. |

A list of the 20 signaling questions from the PROBAST criteria. Questions that apply to the research field under review have been adopted in the paper, as detailed below.

*PROBAST bias assessment criteria modifications for this review:*

**Domain 1** **– Participants**: All signalling questions included in the bias assessment.

**Domain 2** **– Predictors**: Predictors have limited use for deep learning of CT or MRI data and thus could not be assessed.

**Domain 3 – Outcomes**: Signalling questions 3.3, 3.5, and 3.6 regarding predictors were not assessed due to their limited applicability for deep learning.

**Domain 4** **– Analysis**: Signalling questions 4.2, 4.5, 4.6 and 4.9 regarding predictors were not assessed due to their limited applicability for deep learning.

**Applicability**: There were no specific therapeutic questions posed on applicability in the studies under review, and thus could not be assessed.

**Additional file 1: Table S4** Result of CLAIM

| Study ID | 1 | 2 | 3 | 4 | 5 | 6 | 7 | 8 | 9 | 10 | 11 | 12 | 13 | 14 | 15 | 16 | 17 | 18 | 19 | 20 | 21 | 22 | 23 | 24 | 25 | 26 | 27 | 28 | 29 | 30 | 31 | 32 | 33 | 34 | 35 | 36 | 37 | 38 | 39 | 40 | 41 | 42 |
| --- | --- | --- | --- | --- | --- | --- | --- | --- | --- | --- | --- | --- | --- | --- | --- | --- | --- | --- | --- | --- | --- | --- | --- | --- | --- | --- | --- | --- | --- | --- | --- | --- | --- | --- | --- | --- | --- | --- | --- | --- | --- | --- |
| Dai et al.（2021）（27） | 1 | 1 | 1 | 1 | 1 | 1 | 1 | 0 | 0 | 0 | 0 | 0 | 0 | 1 | 1 | 0 | 1 | 0 | 0 | 0 | 0 | 1 | 1 | 0 | 1 | 1 | 1 | 1 | 1 | 0 | 1 | 1 | 0 | 0 | 0 | 0 | 0 | 1 | 1 | 1 | 1 | 1 |
| Tao et al.（2019）（28） | 1 | 1 | 1 | 1 | 1 | 1 | 1 | 0 | 0 | 1 | 1 | 0 | 0 | 1 | 1 | 1 | 0 | 0 | 0 | 0 | 0 | 1 | 0 | 1 | 1 | 1 | 1 | 1 | 1 | 1 | 1 | 0 | 0 | 0 | 0 | 0 | 1 | 1 | 1 | 1 | 1 | 1 |
| Korte et al.（2021）（29） | 1 | 1 | 1 | 1 | 1 | 1 | 1 | 0 | 1 | 1 | 1 | 0 | 0 | 1 | 1 | 1 | 0 | 0 | 0 | 0 | 0 | 0 | 0 | 0 | 1 | 1 | 1 | 1 | 1 | 1 | 0 | 0 | 0 | 0 | 0 | 0 | 0 | 1 | 1 | 1 | 1 | 1 |
| Oktay et al.（2020）（17） | 0 | 1 | 1 | 1 | 1 | 1 | 1 | 1 | 0 | 1 | 0 | 0 | 0 | 1 | 1 | 1 | 0 | 1 | 0 | 1 | 1 | 1 | 0 | 1 | 1 | 1 | 1 | 1 | 1 | 0 | 1 | 1 | 1 | 0 | 1 | 0 | 0 | 1 | 1 | 0 | 0 | 0 |
| Ye et al.（2022）（18） | 1 | 1 | 1 | 1 | 1 | 1 | 1 | 0 | 0 | 1 | 1 | 0 | 0 | 1 | 1 | 1 | 0 | 1 | 0 | 0 | 0 | 1 | 1 | 1 | 1 | 1 | 1 | 1 | 1 | 0 | 1 | 1 | 0 | 1 | 1 | 0 | 0 | 1 | 1 | 1 | 1 | 1 |
| Chan et al.（2019）（30） | 1 | 1 | 1 | 1 | 1 | 1 | 1 | 0 | 1 | 1 | 1 | 0 | 0 | 1 | 1 | 0 | 0 | 0 | 0 | 0 | 0 | 1 | 1 | 1 | 1 | 1 | 1 | 1 | 1 | 0 | 1 | 0 | 0 | 0 | 0 | 0 | 0 | 1 | 1 | 0 | 1 | 1 |
| Chen et al.（2021）（23） | 1 | 1 | 1 | 1 | 1 | 1 | 1 | 0 | 0 | 1 | 1 | 0 | 1 | 1 | 1 | 1 | 0 | 0 | 0 | 0 | 0 | 1 | 1 | 1 | 1 | 1 | 1 | 1 | 1 | 0 | 1 | 1 | 0 | 0 | 0 | 0 | 1 | 1 | 1 | 1 | 1 | 1 |
| Liang et al.（2019）（31） | 1 | 1 | 1 | 1 | 1 | 1 | 1 | 0 | 1 | 1 | 0 | 0 | 0 | 1 | 1 | 1 | 1 | 0 | 0 | 0 | 0 | 0 | 0 | 1 | 1 | 1 | 1 | 1 | 1 | 1 | 1 | 0 | 0 | 0 | 0 | 1 | 0 | 1 | 1 | 1 | 1 | 1 |
| Kim et al.（2021）（32） | 1 | 1 | 1 | 1 | 1 | 1 | 1 | 1 | 1 | 1 | 1 | 0 | 0 | 1 | 1 | 1 | 1 | 1 | 0 | 0 | 0 | 1 | 0 | 0 | 0 | 1 | 1 | 1 | 1 | 0 | 1 | 0 | 0 | 0 | 0 | 0 | 0 | 1 | 1 | 1 | 1 | 1 |
| Gao et al.（2021）（33） | 1 | 1 | 1 | 1 | 1 | 1 | 1 | 0 | 0 | 1 | 1 | 0 | 0 | 1 | 1 | 0 | 0 | 0 | 0 | 0 | 0 | 1 | 0 | 1 | 1 | 1 | 1 | 1 | 1 | 0 | 1 | 1 | 0 | 0 | 1 | 0 | 1 | 1 | 1 | 1 | 1 | 1 |
| Nuo et al.（2018）（34） | 1 | 1 | 1 | 1 | 1 | 1 | 1 | 0 | 1 | 1 | 1 | 0 | 0 | 1 | 1 | 0 | 0 | 0 | 0 | 0 | 0 | 1 | 0 | 1 | 1 | 1 | 1 | 1 | 1 | 1 | 0 | 0 | 0 | 0 | 1 | 1 | 1 | 1 | 1 | 1 | 1 | 1 |
| Fang et al.（2021）（35） | 1 | 1 | 1 | 1 | 1 | 1 | 1 | 0 | 0 | 1 | 1 | 0 | 0 | 1 | 1 | 0 | 0 | 0 | 1 | 1 | 0 | 1 | 0 | 0 | 1 | 1 | 1 | 1 | 1 | 0 | 1 | 0 | 0 | 0 | 1 | 0 | 0 | 1 | 1 | 1 | 1 | 1 |
| V van Dijk et al.（2020）（36） | 1 | 1 | 1 | 1 | 1 | 1 | 1 | 1 | 0 | 1 | 1 | 0 | 0 | 1 | 1 | 1 | 0 | 1 | 0 | 0 | 0 | 0 | 0 | 0 | 0 | 0 | 0 | 1 | 1 | 1 | 1 | 0 | 0 | 1 | 1 | 0 | 0 | 0 | 1 | 0 | 1 | 0 |
| Dai et al.（2022）（37） | 1 | 1 | 1 | 1 | 1 | 1 | 1 | 1 | 0 | 1 | 0 | 0 | 0 | 1 | 1 | 0 | 1 | 0 | 0 | 0 | 0 | 1 | 0 | 1 | 1 | 0 | 1 | 1 | 1 | 0 | 1 | 0 | 0 | 0 | 0 | 0 | 0 | 0 | 1 | 0 | 1 | 0 |
| Tappeiner et al.（2019）（38） | 1 | 1 | 1 | 1 | 1 | 1 | 1 | 0 | 0 | 1 | 0 | 0 | 0 | 1 | 1 | 1 | 0 | 0 | 0 | 0 | 0 | 1 | 1 | 0 | 1 | 1 | 1 | 1 | 1 | 0 | 1 | 0 | 0 | 0 | 1 | 0 | 1 | 0 | 1 | 0 | 1 | 0 |
| Gou et al.（2020）（39） | 1 | 1 | 1 | 1 | 1 | 1 | 0 | 0 | 0 | 0 | 1 | 0 | 1 | 1 | 1 | 1 | 0 | 0 | 0 | 0 | 0 | 1 | 0 | 0 | 1 | 1 | 1 | 1 | 1 | 1 | 1 | 0 | 0 | 0 | 1 | 1 | 0 | 1 | 1 | 1 | 1 | 0 |
| Nuo et al.（2019）（40） | 1 | 1 | 1 | 1 | 1 | 1 | 1 | 0 | 1 | 1 | 1 | 0 | 0 | 1 | 1 | 0 | 0 | 0 | 0 | 1 | 0 | 1 | 1 | 1 | 1 | 1 | 1 | 1 | 1 | 0 | 1 | 1 | 0 | 0 | 1 | 0 | 1 | 0 | 0 | 1 | 1 | 1 |
| Zhang et al.（2021）（41） | 1 | 1 | 1 | 1 | 1 | 1 | 1 | 0 | 1 | 1 | 1 | 0 | 0 | 1 | 1 | 0 | 0 | 0 | 0 | 1 | 0 | 1 | 0 | 0 | 0 | 1 | 1 | 1 | 1 | 0 | 1 | 0 | 0 | 0 | 0 | 0 | 0 | 0 | 1 | 1 | 1 | 1 |
| Zhao et al.（2021）（42） | 1 | 1 | 1 | 1 | 1 | 1 | 1 | 0 | 0 | 1 | 1 | 0 | 0 | 1 | 1 | 1 | 0 | 0 | 0 | 1 | 0 | 1 | 0 | 0 | 1 | 1 | 1 | 1 | 1 | 0 | 1 | 1 | 0 | 0 | 1 | 0 | 0 | 1 | 1 | 0 | 1 | 0 |
| Liang et al.（2020）（43） | 1 | 1 | 1 | 1 | 1 | 1 | 1 | 0 | 0 | 1 | 1 | 0 | 0 | 1 | 1 | 0 | 0 | 0 | 0 | 0 | 0 | 1 | 0 | 1 | 1 | 1 | 1 | 1 | 1 | 0 | 1 | 1 | 0 | 0 | 0 | 0 | 0 | 1 | 1 | 1 | 0 | 1 |
| Koo et al.（2022）（44） | 1 | 1 | 1 | 1 | 1 | 1 | 1 | 1 | 1 | 1 | 1 | 0 | 0 | 1 | 1 | 1 | 1 | 0 | 0 | 1 | 0 | 1 | 0 | 0 | 1 | 1 | 1 | 1 | 1 | 0 | 1 | 0 | 0 | 0 | 0 | 0 | 1 | 0 | 1 | 0 | 1 | 0 |
| Rooij et al.（2019）（20） | 1 | 1 | 1 | 1 | 1 | 1 | 1 | 0 | 1 | 1 | 1 | 0 | 1 | 1 | 1 | 0 | 0 | 0 | 0 | 0 | 0 | 1 | 1 | 0 | 1 | 1 | 1 | 1 | 1 | 1 | 1 | 0 | 0 | 0 | 0 | 0 | 1 | 1 | 1 | 0 | 1 | 0 |

**Additional file 1: Table S5** Result of PROBAST

| Study ID | Participants  (1) | Participants (2) | Outcome (1) | Outcome (2) | Outcome (4) | Analysis (1) | Analysis (3) | Analysis (4) | Analysis (7) | Analysis (8) | Participants  （Summary） | Outcome  （Summary） | Analysis  （Summary） | Overall |
| --- | --- | --- | --- | --- | --- | --- | --- | --- | --- | --- | --- | --- | --- | --- |
| Dai et al.（2021）（27） | L | U | L | L | L | L | L | H | L | L | U | L | H | H |
| Tao et al.（2019）（28） | L | H | L | L | L | U | L | H | L | H | H | L | H | H |
| Korte et al.（2021）（29） | L | U | L | L | L | H | L | H | L | L | U | L | H | H |
| Oktay et al.（2020）（17） | L | U | L | L | L | L | L | L | L | U | U | L | U | U |
| Ye et al.（2022）（18） | L | U | L | L | L | L | L | U | L | L | U | L | U | U |
| Chan et al.（2019）（30） | L | H | L | L | L | U | L | U | L | L | H | L | U | H |
| Chen et al.（2021）（23） | L | U | L | L | L | L | L | U | L | L | U | L | U | U |
| Liang et al.（2019）（31） | L | U | L | H | L | U | L | U | L | H | U | H | H | H |
| Kim et al.（2021）（32） | L | L | L | L | L | H | L | U | L | L | L | L | H | H |
| Gao et al.（2021）（33） | L | U | L | L | L | U | L | U | L | L | U | L | U | U |
| Nuo et al.（2018）（34） | L | U | L | L | L | U | L | L | L | L | U | L | U | U |
| Fang et al.（2021）（35） | L | U | L | L | L | U | U | U | L | L | U | L | U | U |
| V van Dijk et al.（2020）（36） | L | L | L | L | L | H | L | L | L | L | L | L | H | H |
| Dai et al.（2022）（37） | L | U | L | H | L | L | L | U | L | U | U | H | U | U |
| Tappeiner et al.（2019）（38） | L | L | L | H | L | U | L | U | L | L | L | H | U | H |
| Gou et al.（2020）（39） | L | U | L | L | L | U | L | L | L | L | U | L | U | U |
| Nuo et al.（2019）（40） | L | U | L | L | L | U | L | U | U | L | U | L | U | U |
| Zhang et al.（2021）（41） | L | U | L | L | L | U | U | U | H | H | U | L | H | H |
| Zhao et al.（2021）（42） | L | U | L | L | L | L | L | U | L | L | U | L | U | U |
| Liang et al.（2020）（43） | L | U | L | L | L | L | U | U | L | L | U | L | U | U |
| Koo et al.（2022）（44） | L | L | L | L | L | H | U | U | L | L | L | L | H | H |
| Rooij et al.（2019）（20） | L | U | L | L | L | H | H | H | L | U | U | L | H | H |

Table Additional file 1: Table S5 Result of PROBAST (Prediction model Risk of Bias Assessment Tool) Review Items. “L”represents for “Low” risk of bias， “U” represents for “Unclear” risk of bias， “H” represents for “High” risk of bias

**Additional file 1: Figure S1(A-H)** Forest plot of the pooled DSC of 12 OARs

**Figure 1A** Forest plot of the pooled DSC of brain stem in DL models. **
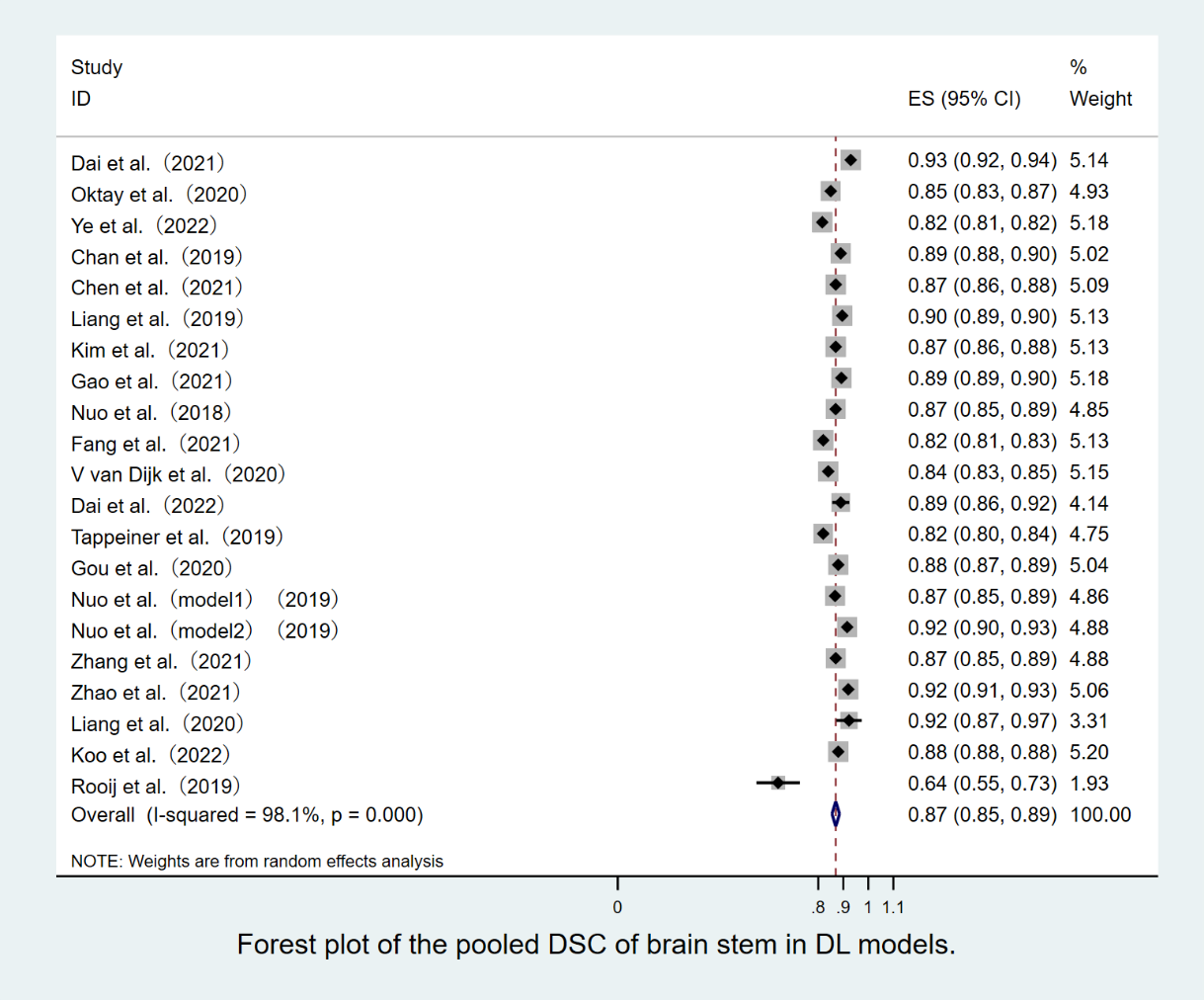
**

**Figure 1B** Forest plot of the pooled DSC of spinal cord in DL models.


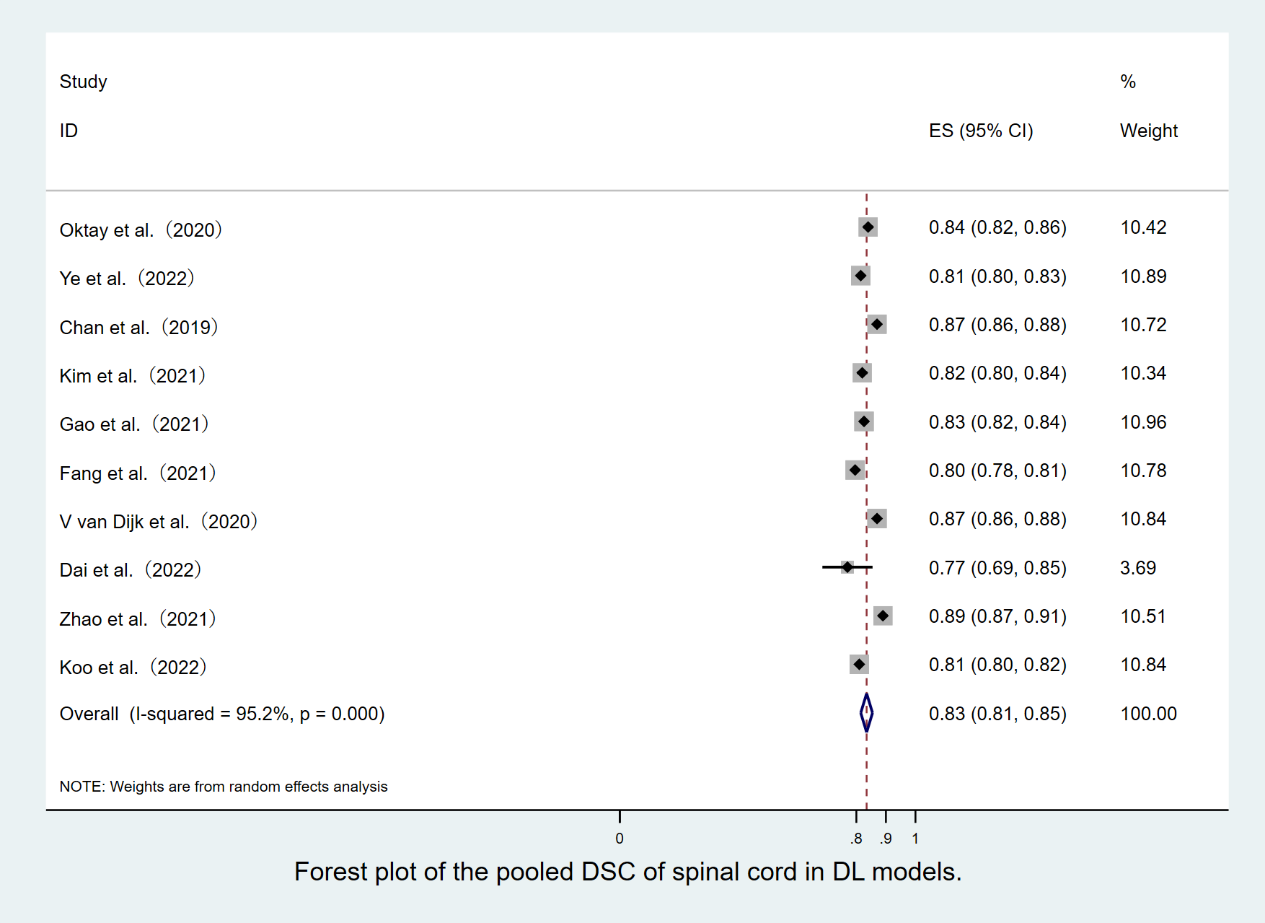


**Figure 1C** Forest plot of the pooled DSC of mandible in DL models.


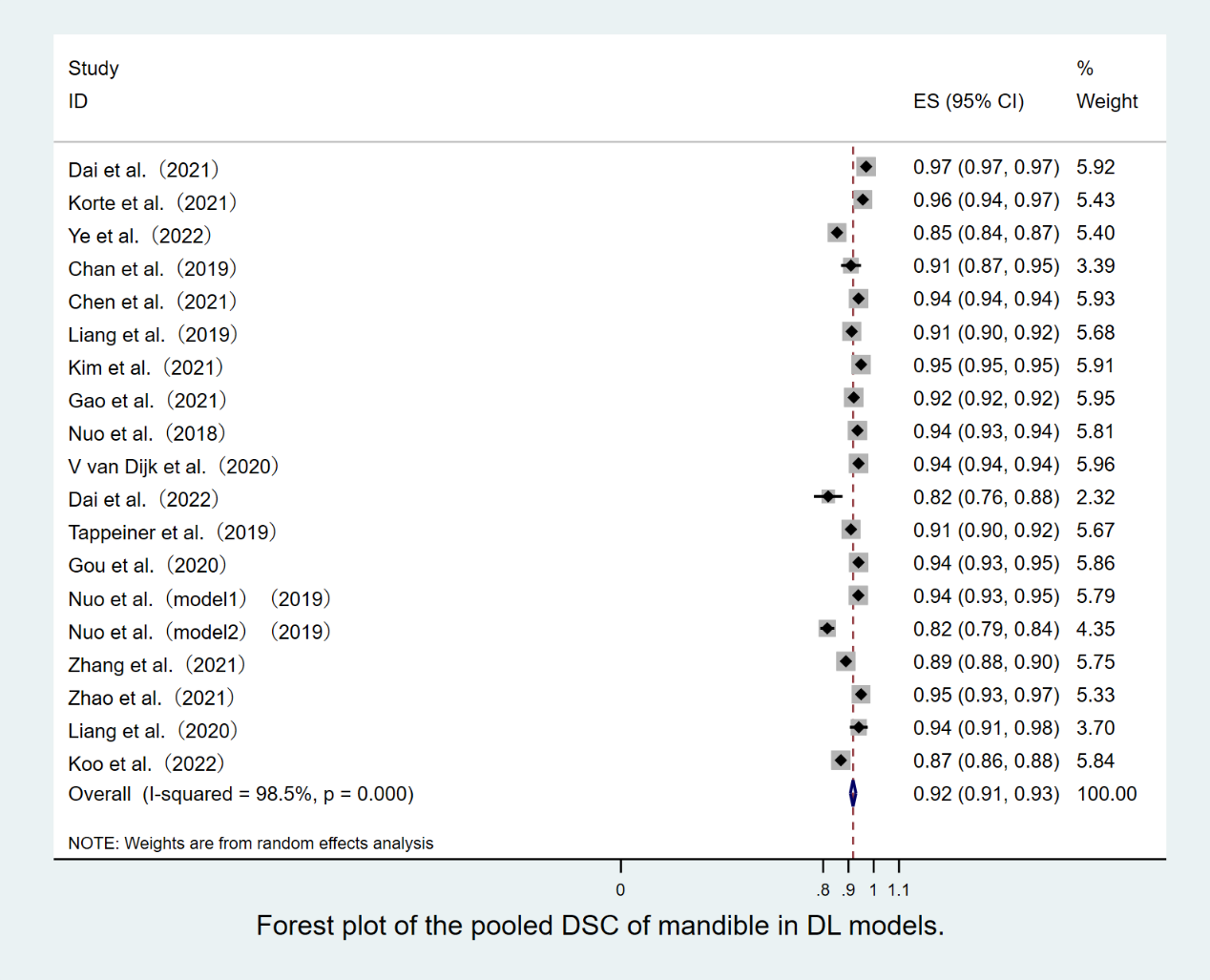


**Figure 1D** Forest plot of the pooled DSC of left eye in DL models.


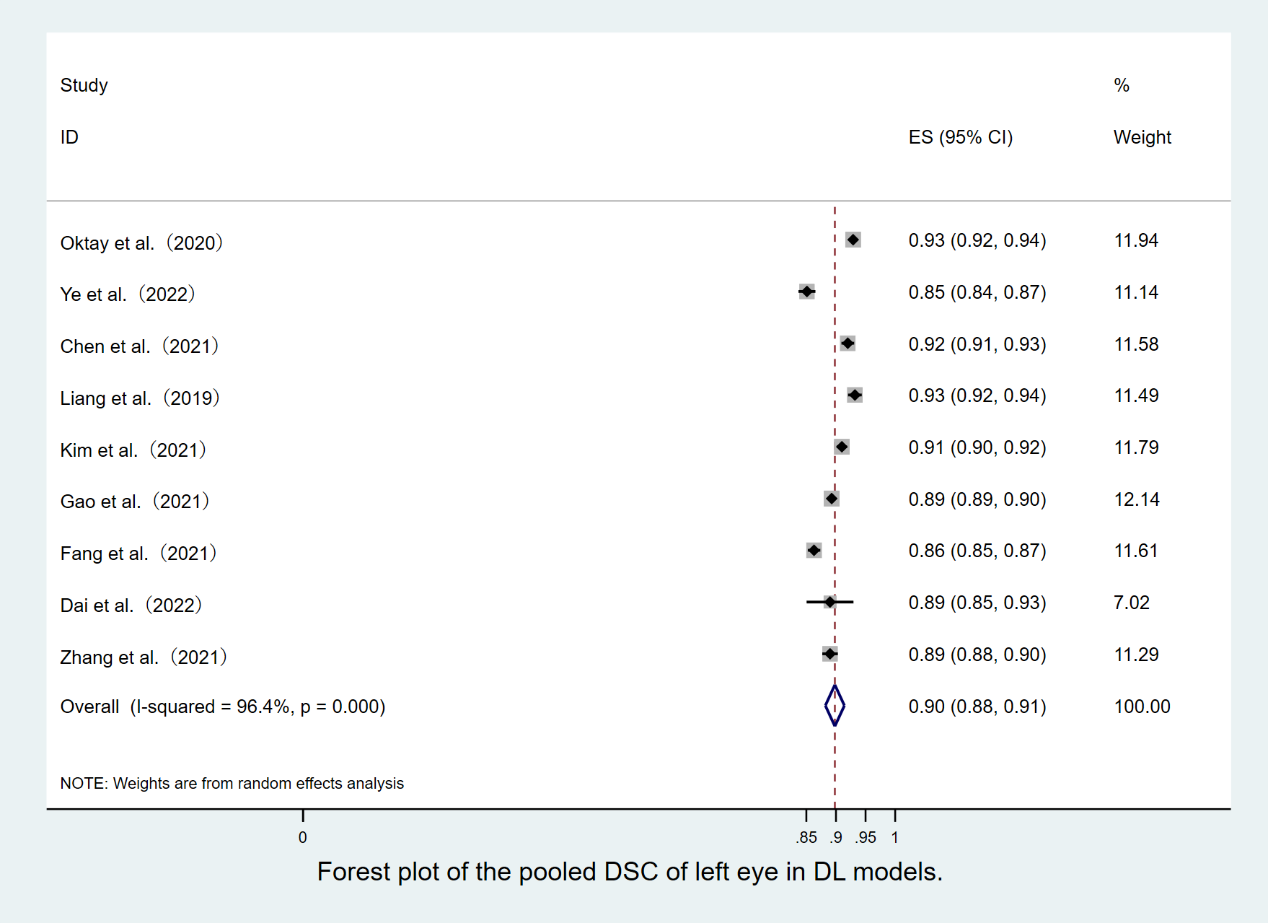


**Figure 1E** Forest plot of the pooled DSC of right eye in DL models.


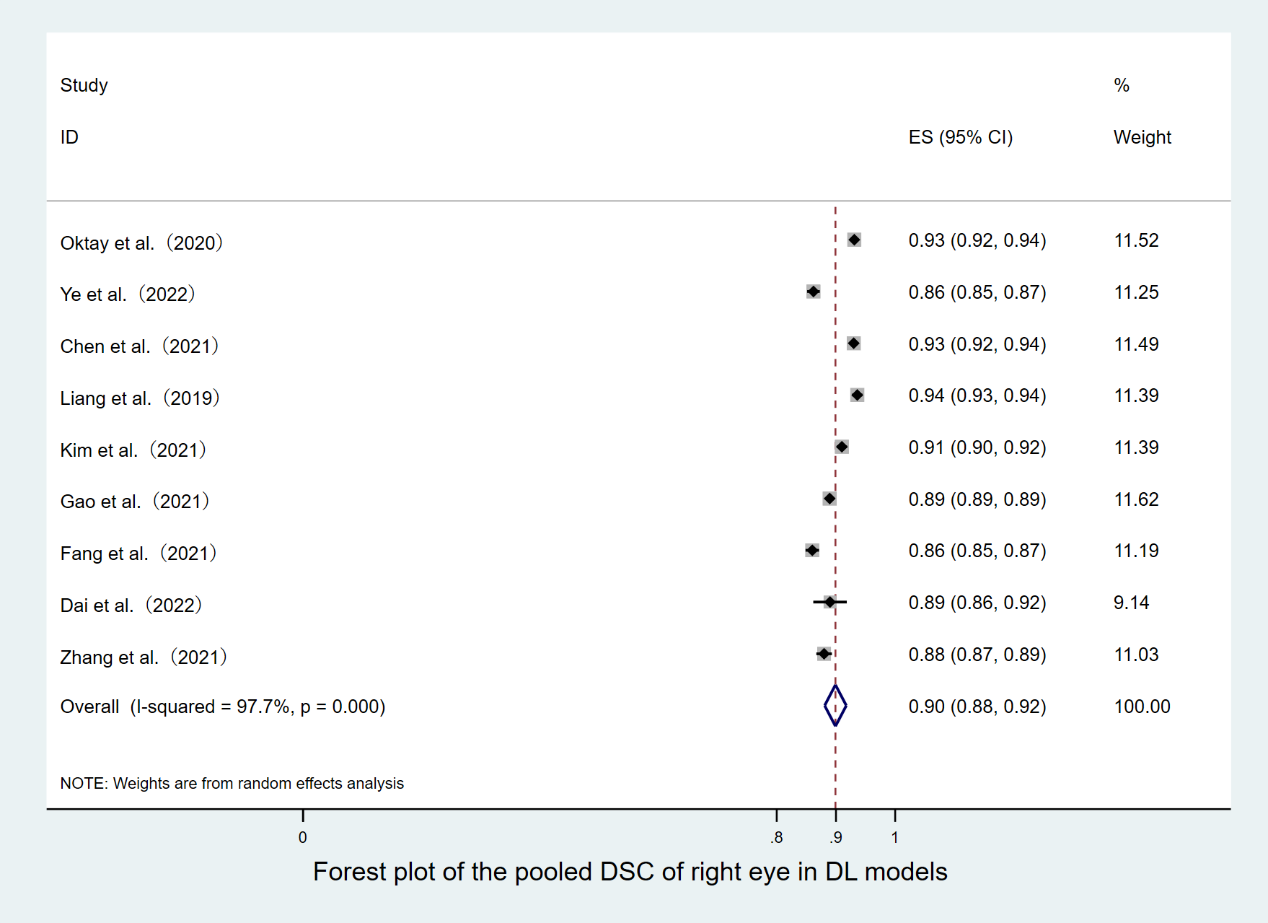


**Figure 1F** Forest plot of the pooled DSC of left optic nerve in DL models.


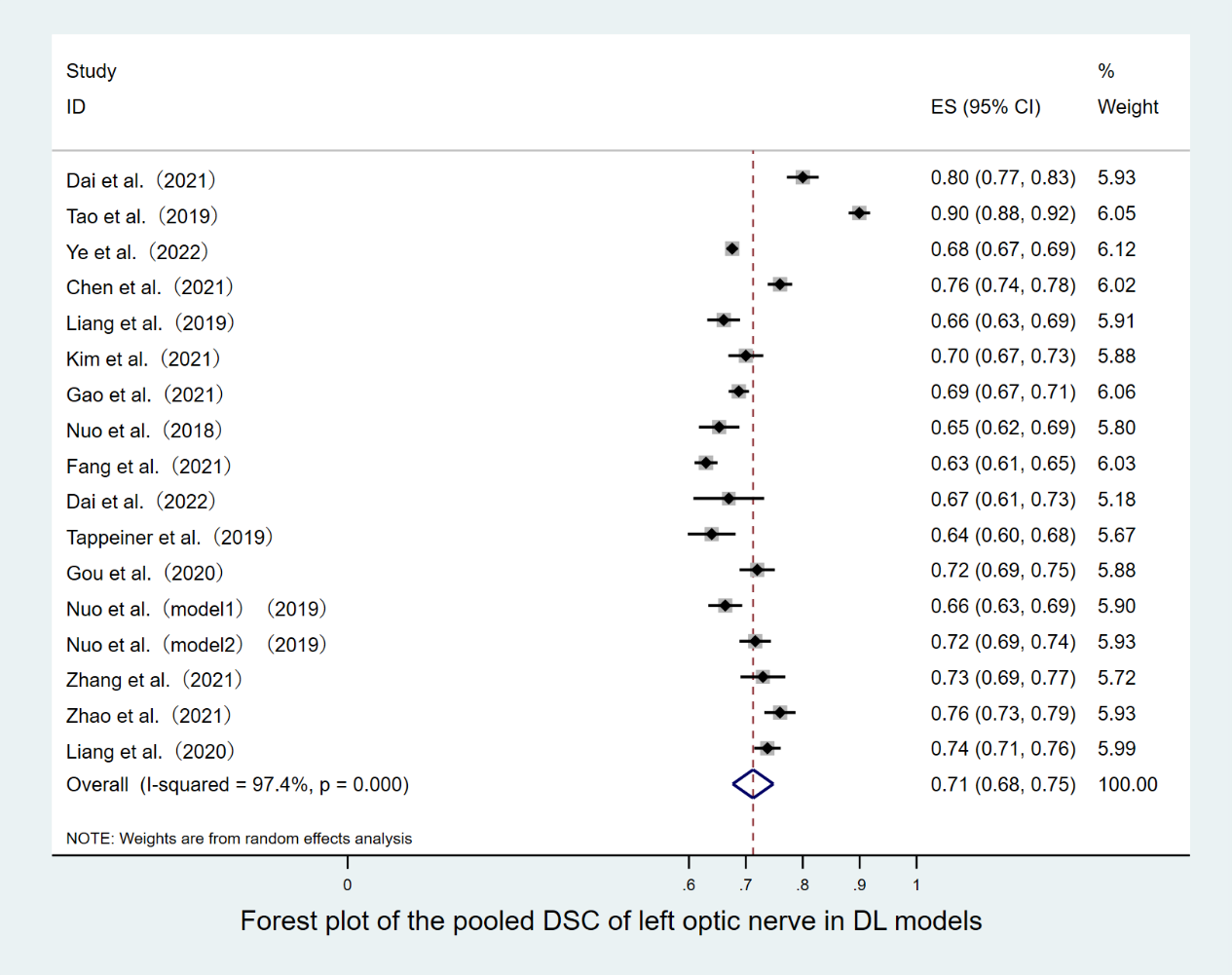


**Figure 1G** Forest plot of the pooled DSC of right optic nerve in DL models.


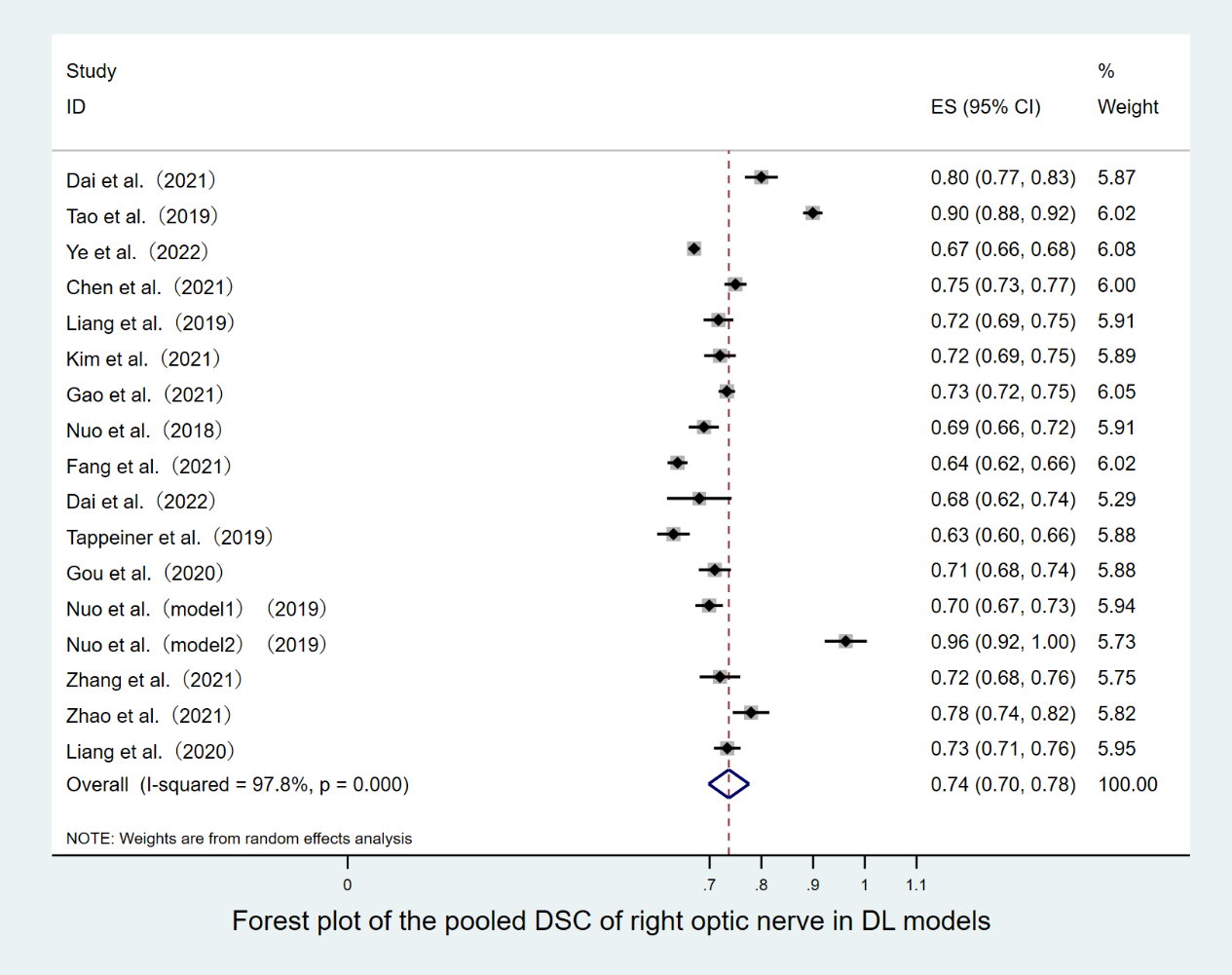


**Figure 1H** Forest plot of the pooled DSC of optic chiasm in DL models.


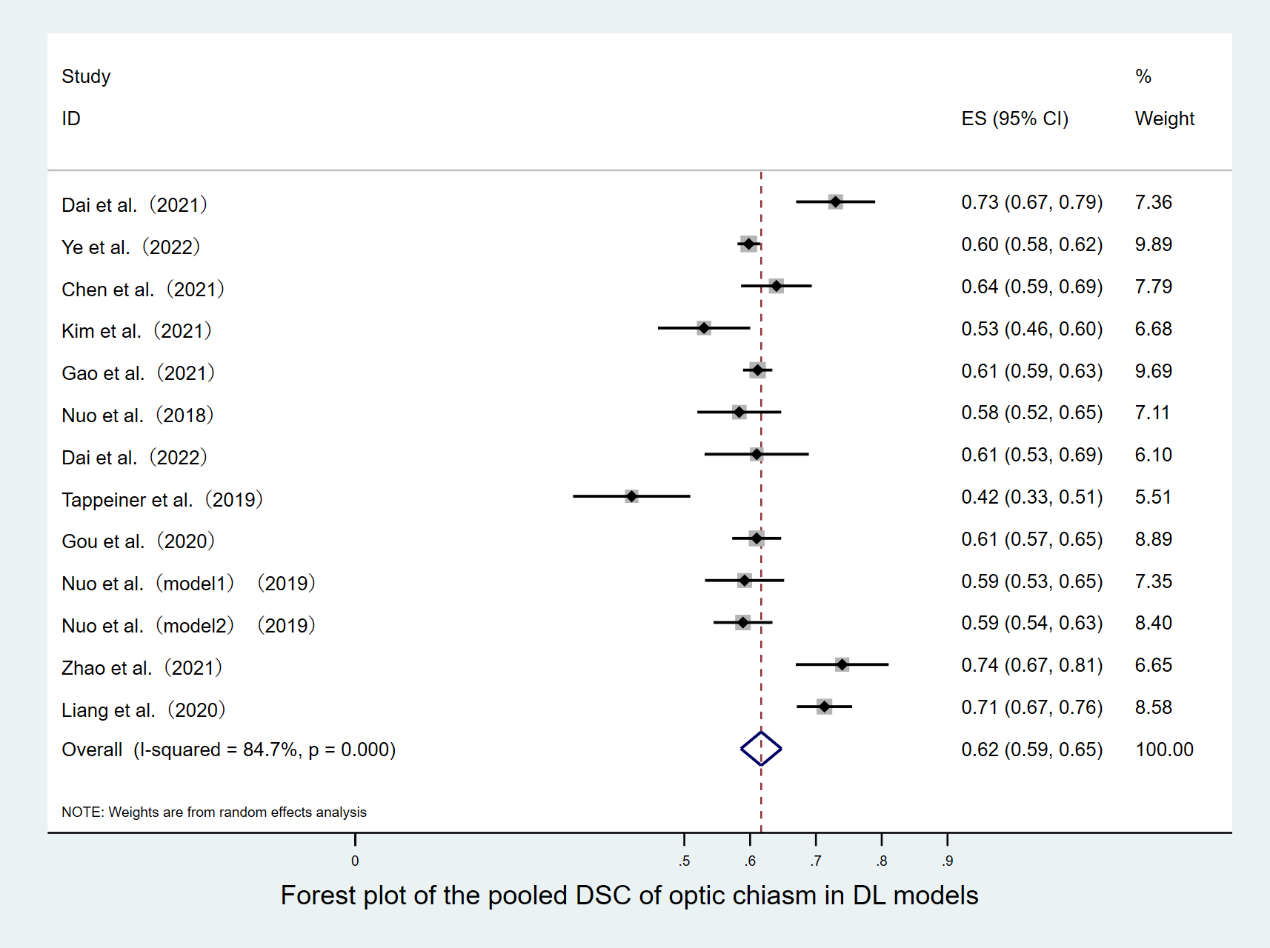


**Figure 1I** Forest plot of the pooled DSC of left parotid gland in DL models.


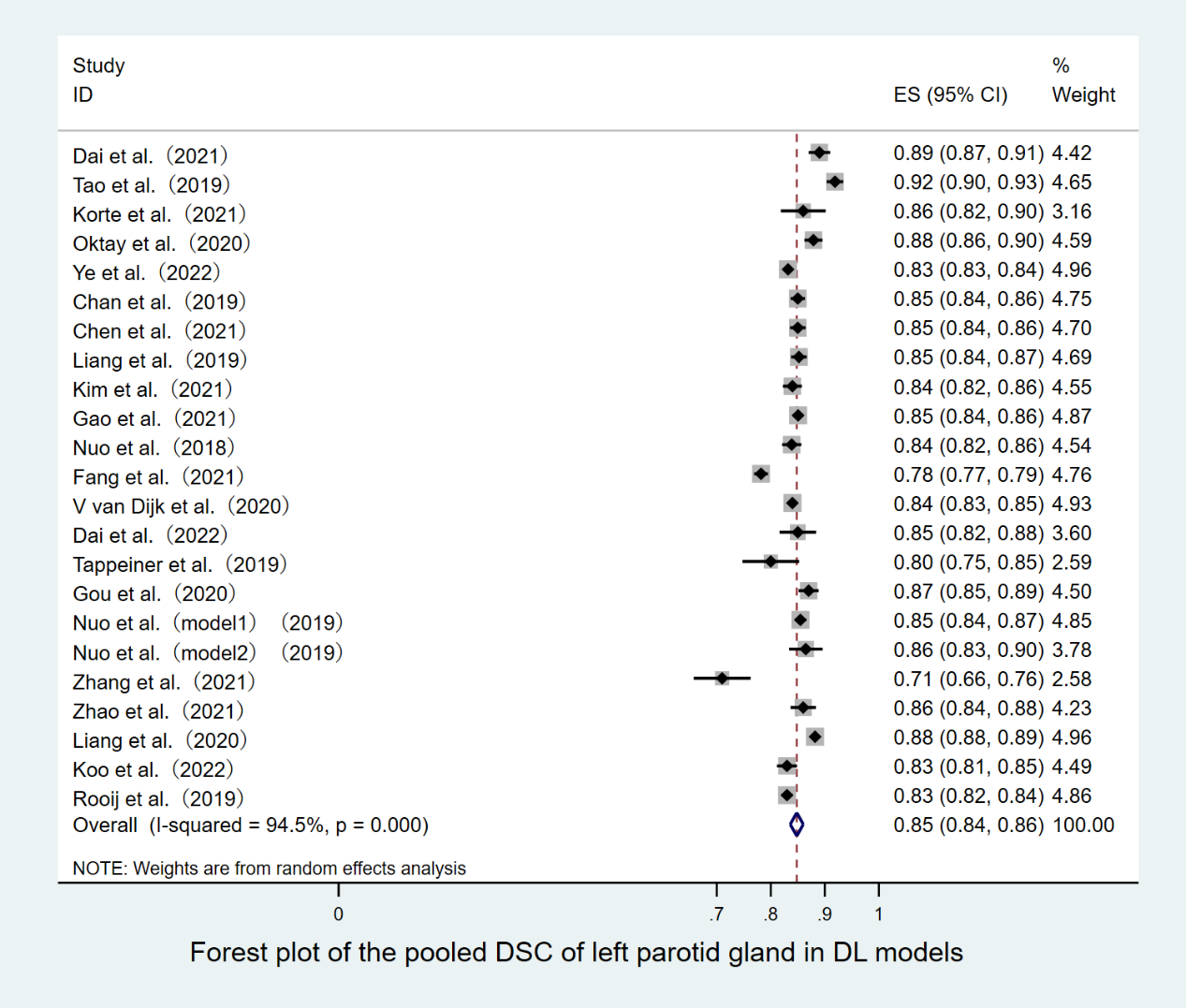


**Figure 1J** Forest plot of the pooled DSC of right parotid gland in DL models.


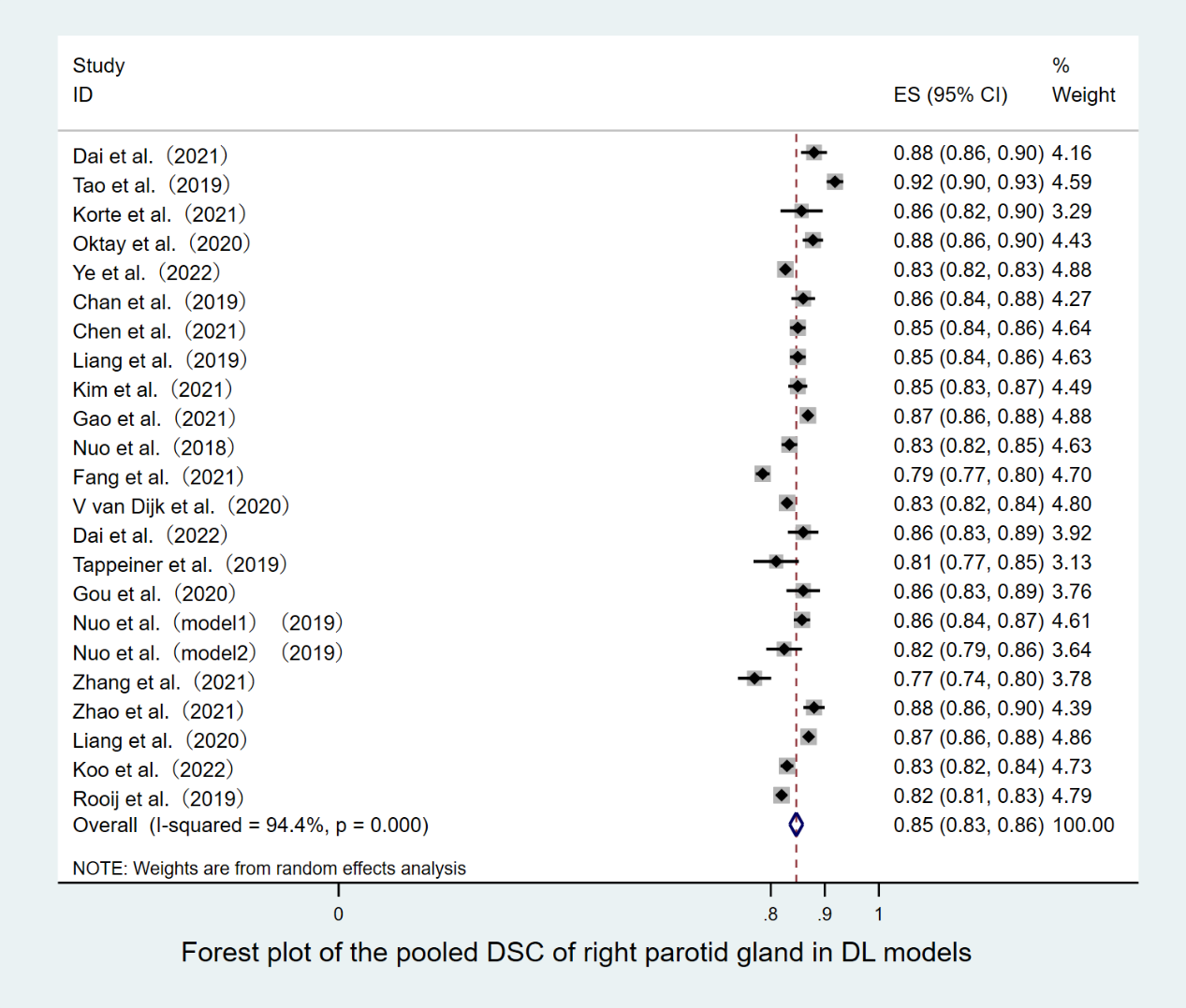


**Figure 1K** Forest plot of the pooled DSC of left submandibular gland in DL models.


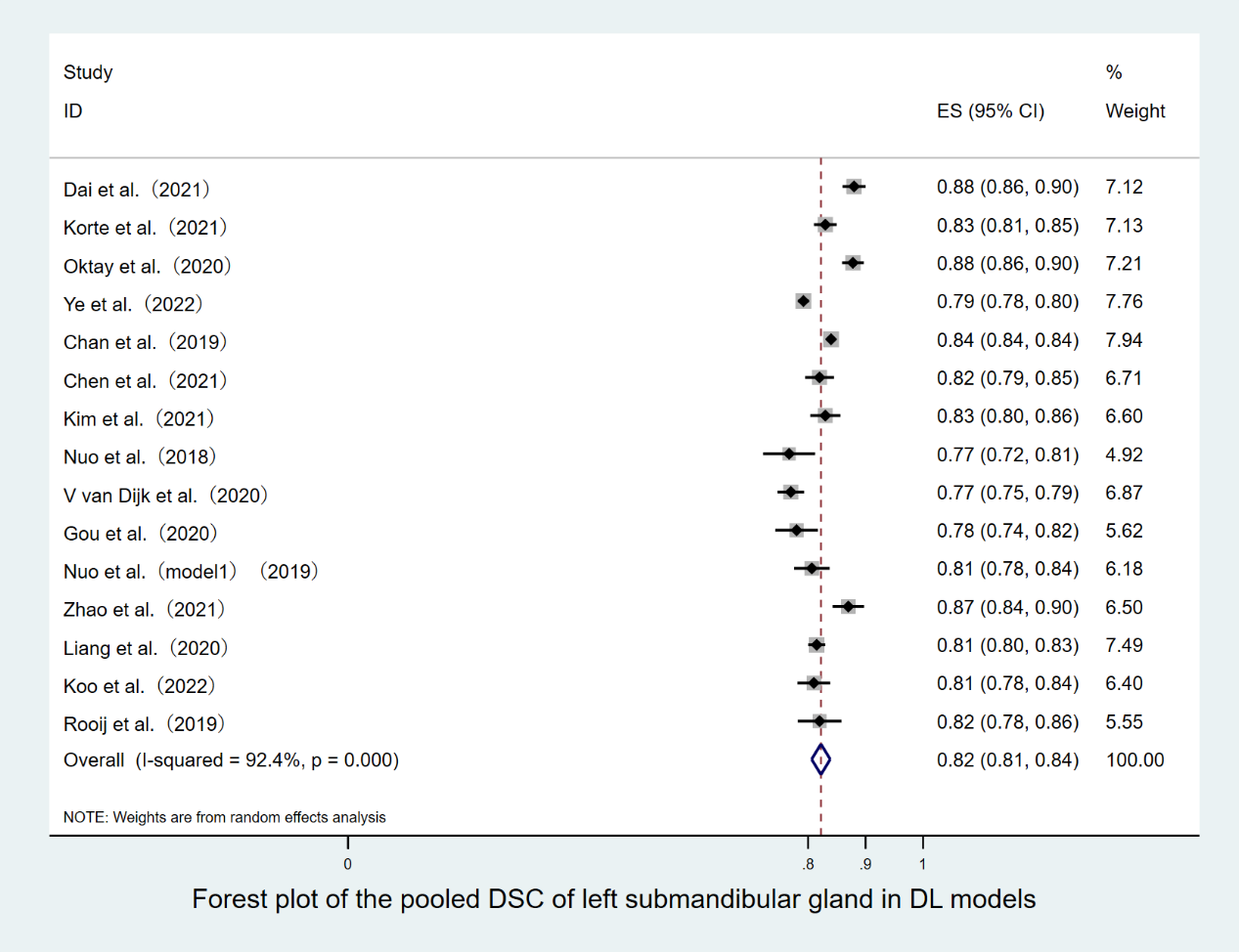


**Figure 1L** Forest plot of the pooled DSC of right submandibular gland in DL models.


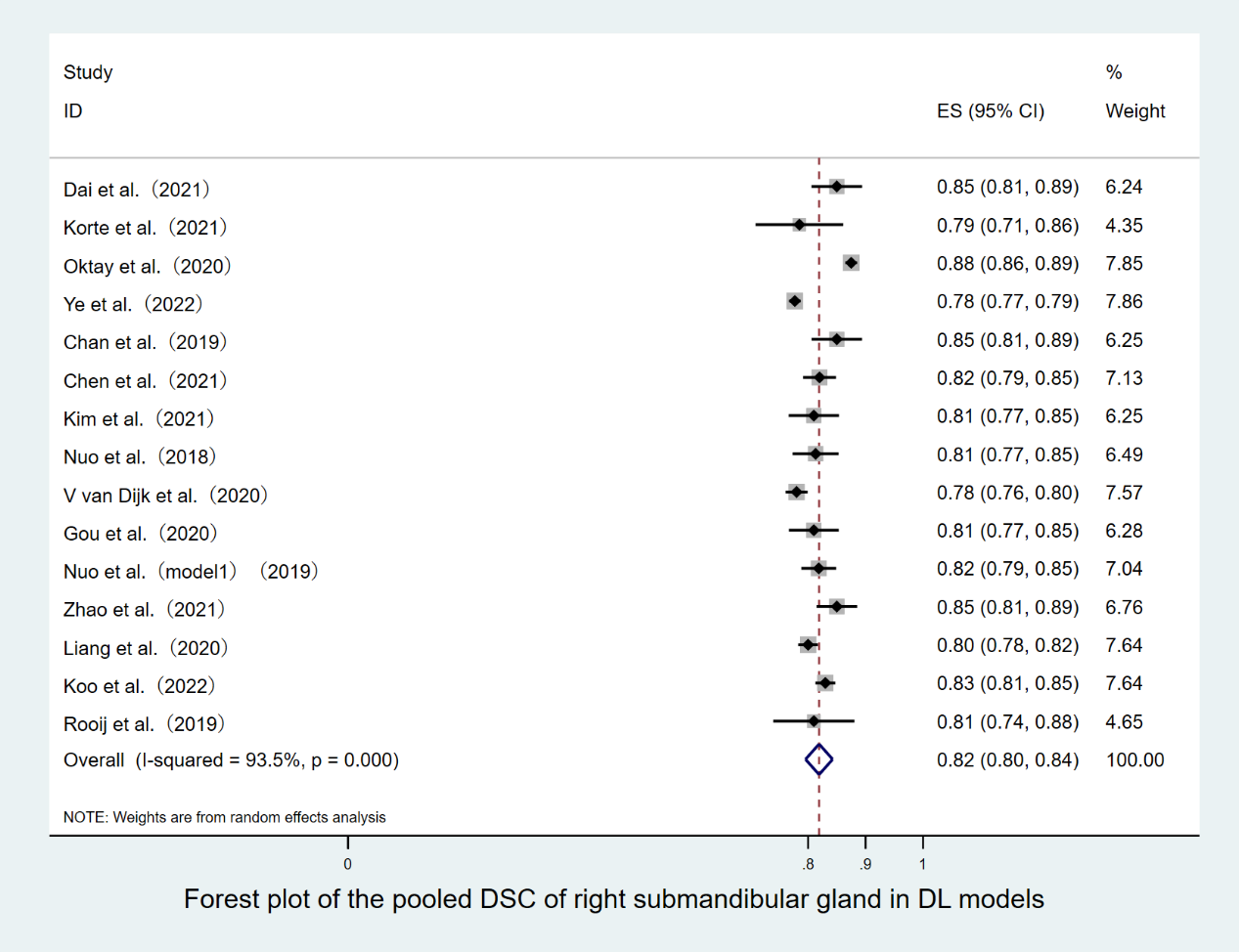


**Additional file 1: Figure S2(A-L)** Funnel plots for meta-analysis of 12 OARs

**Figure 2A** Funnel plots for meta-analysis of brain stem


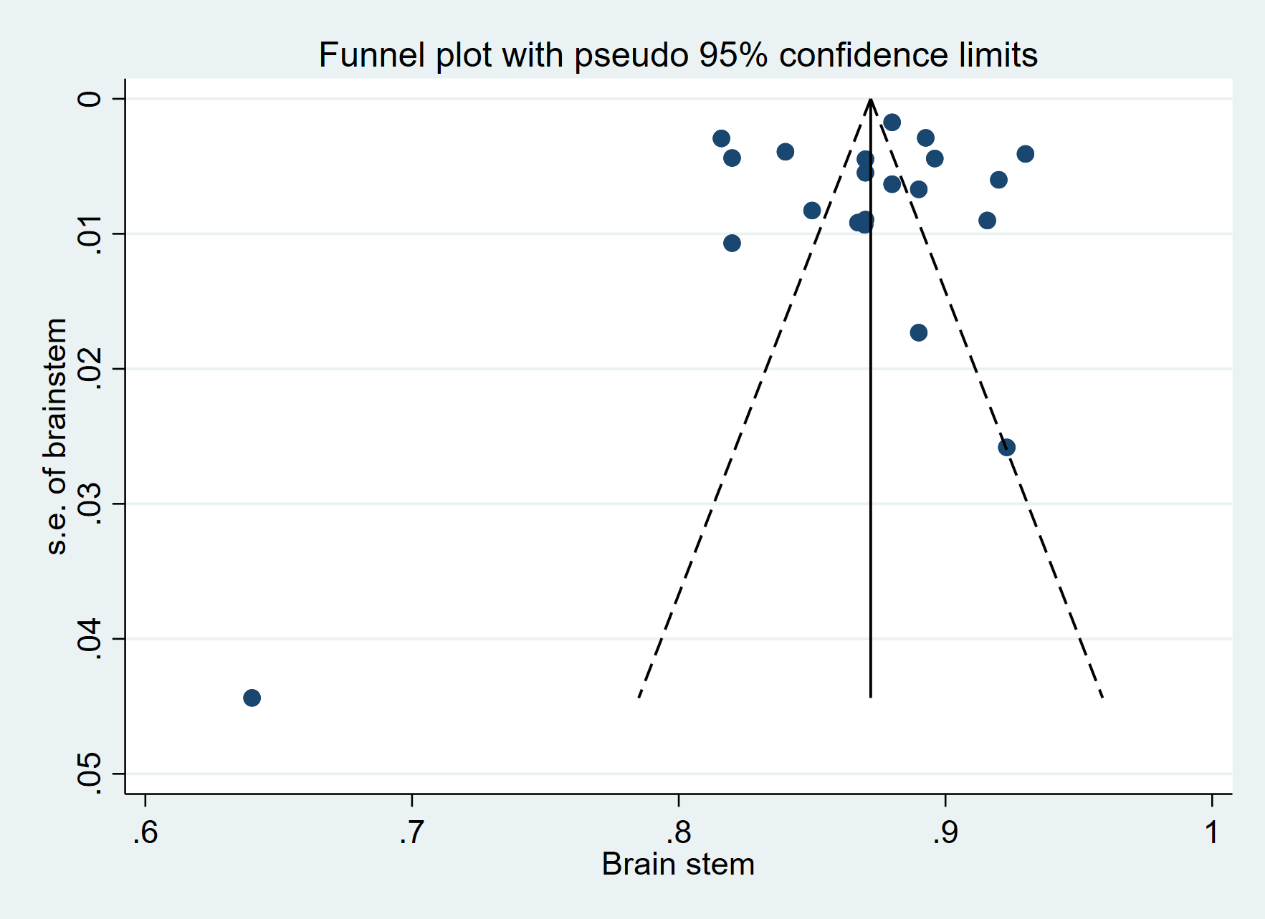


**Figure 2B** Funnel plots for meta-analysis of spinal cord


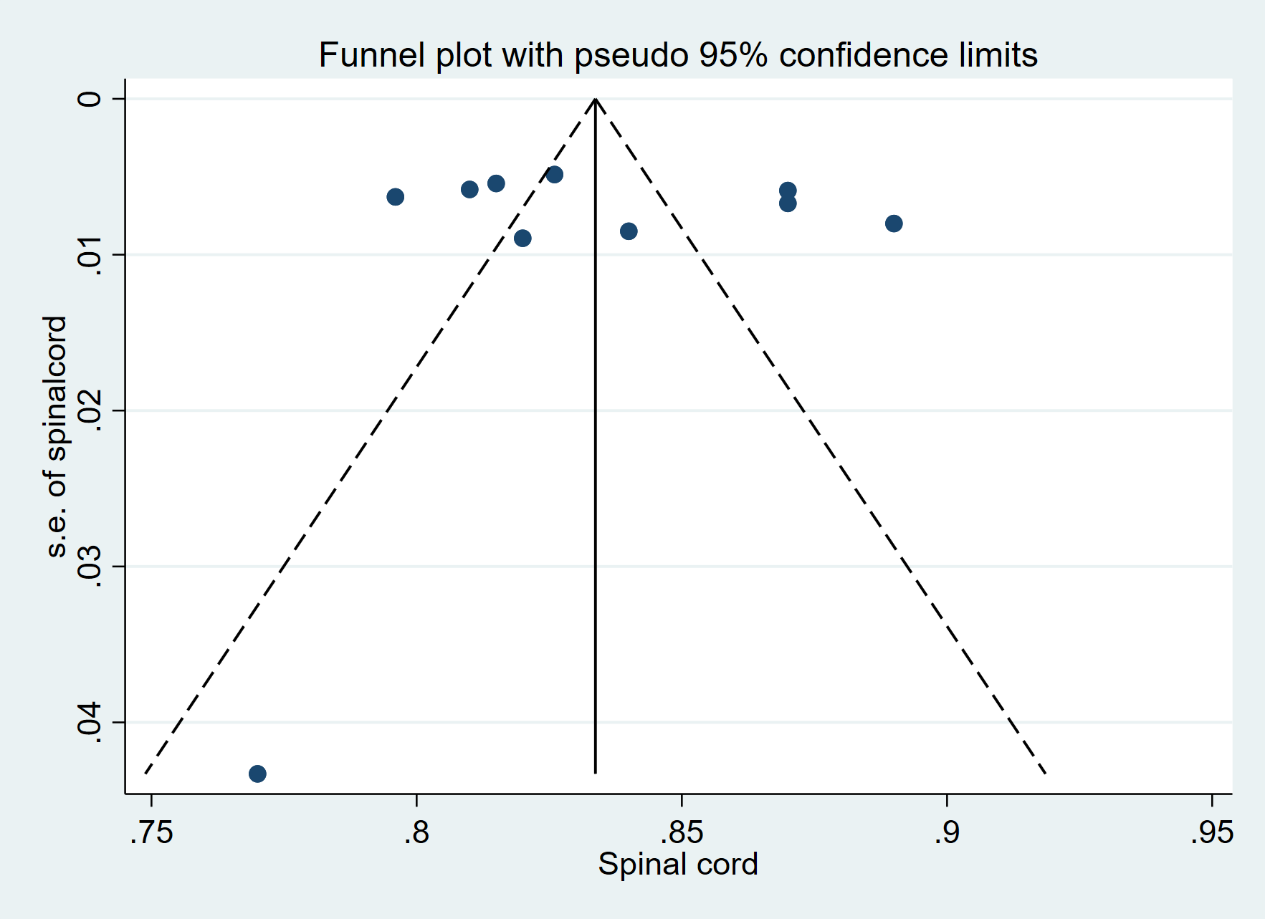


**Figure 2C** Funnel plots for meta-analysis of mandible


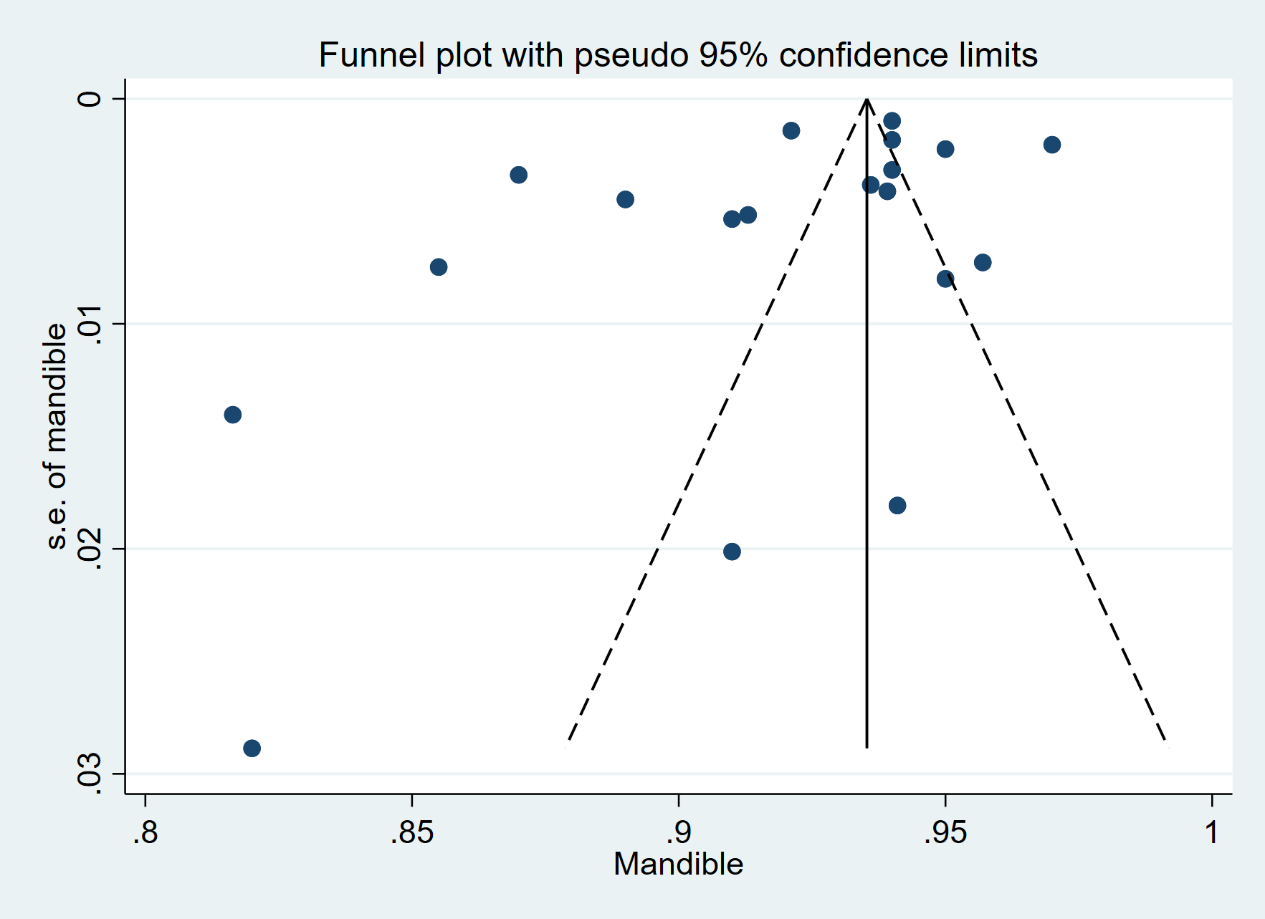


**Figure 2D** Funnel plots for meta-analysis of left eye


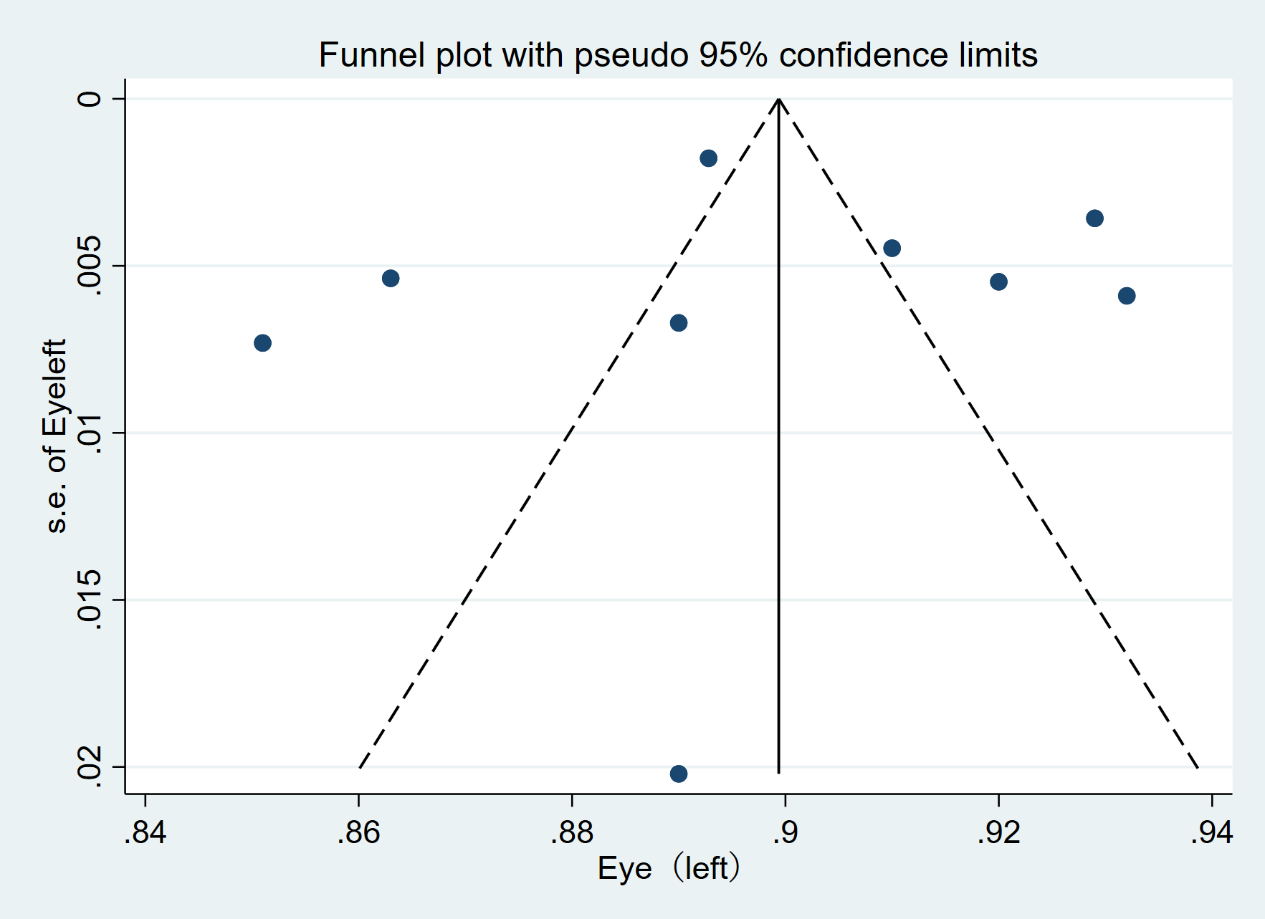


**Figure 2E** Funnel plots for meta-analysis of right eye


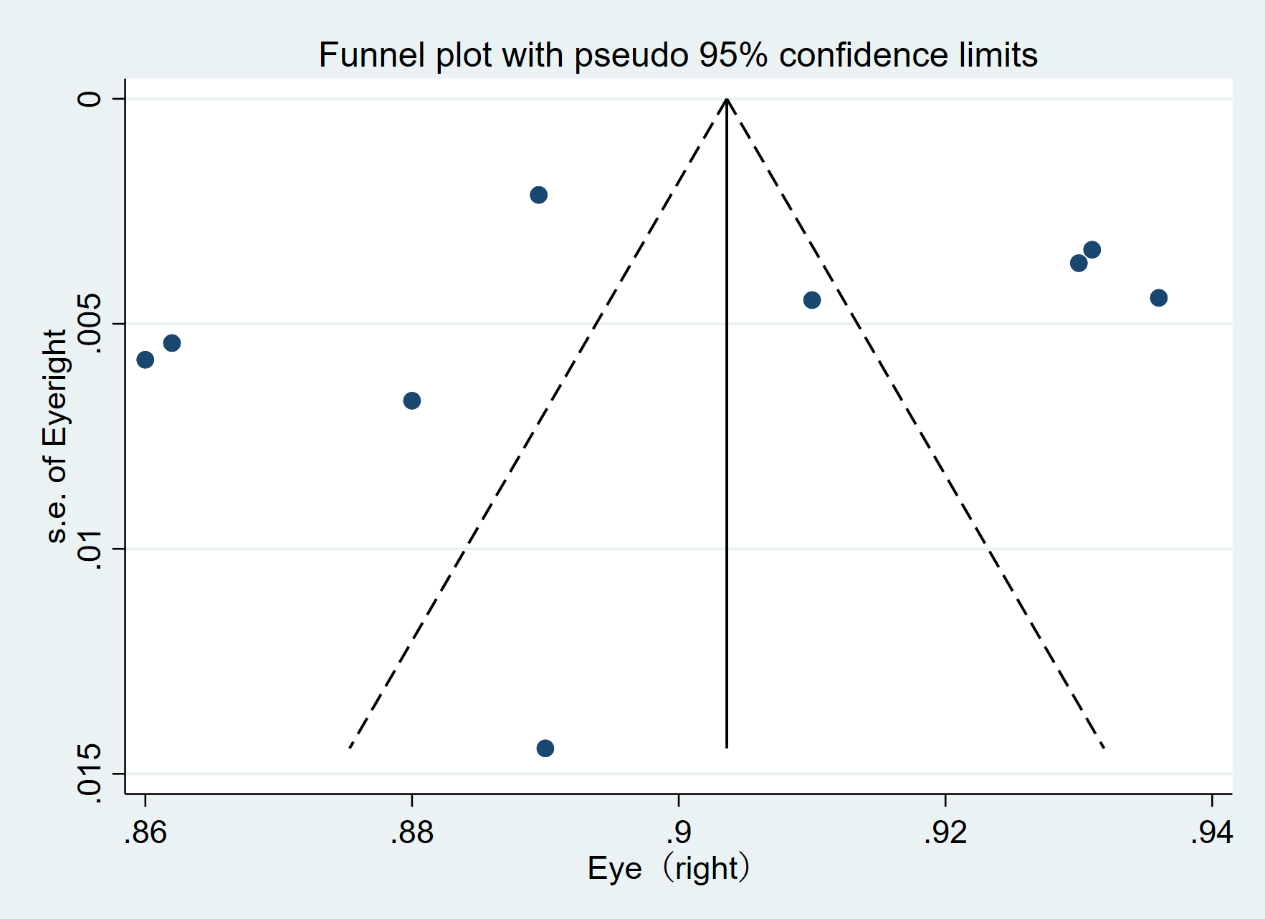


**Figure 2F** Funnel plots for meta-analysis of left optic nerve


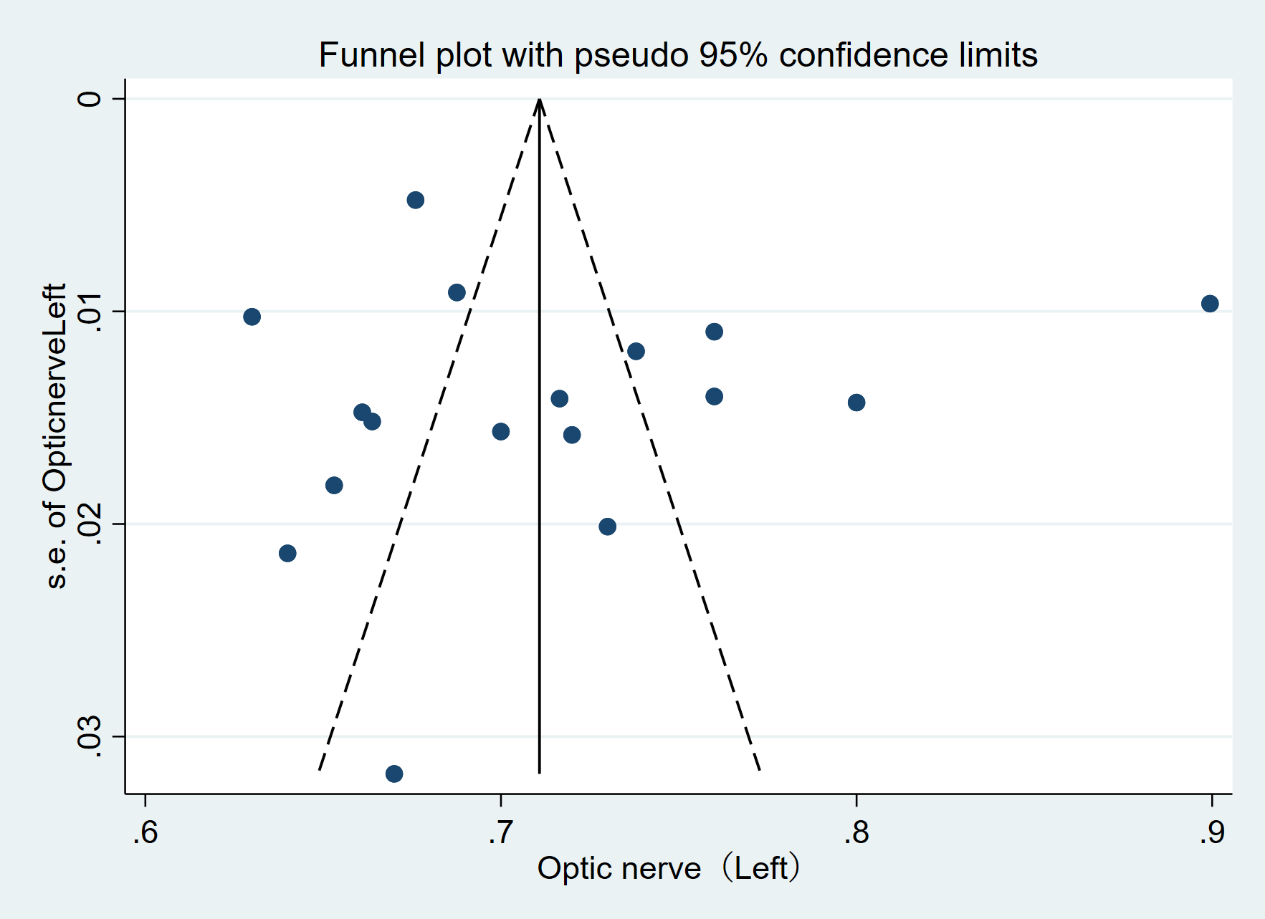


**Figure 2G** Funnel plots for meta-analysis of right optic nerve


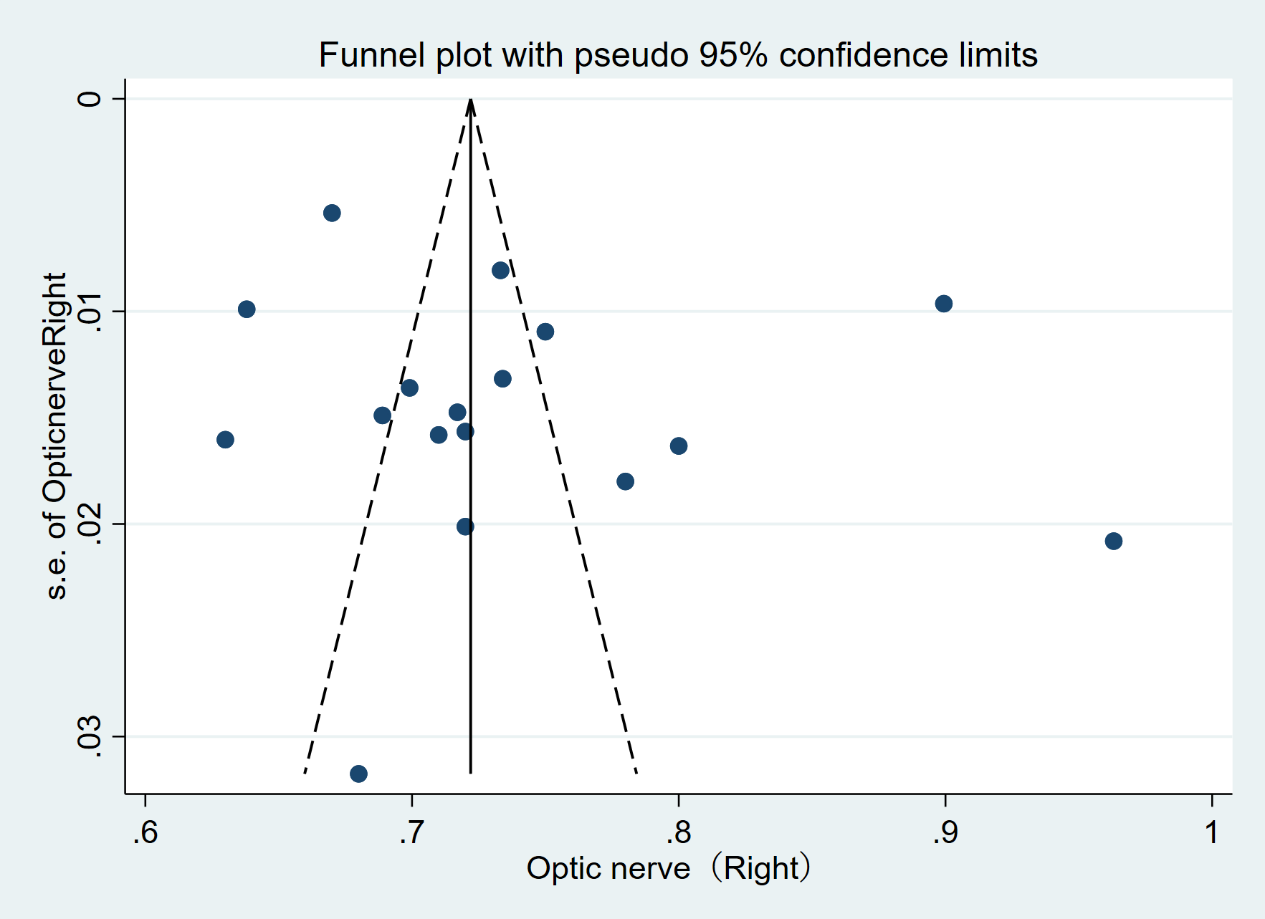


**Figure 2H** Funnel plots for meta-analysis of optic chiasm


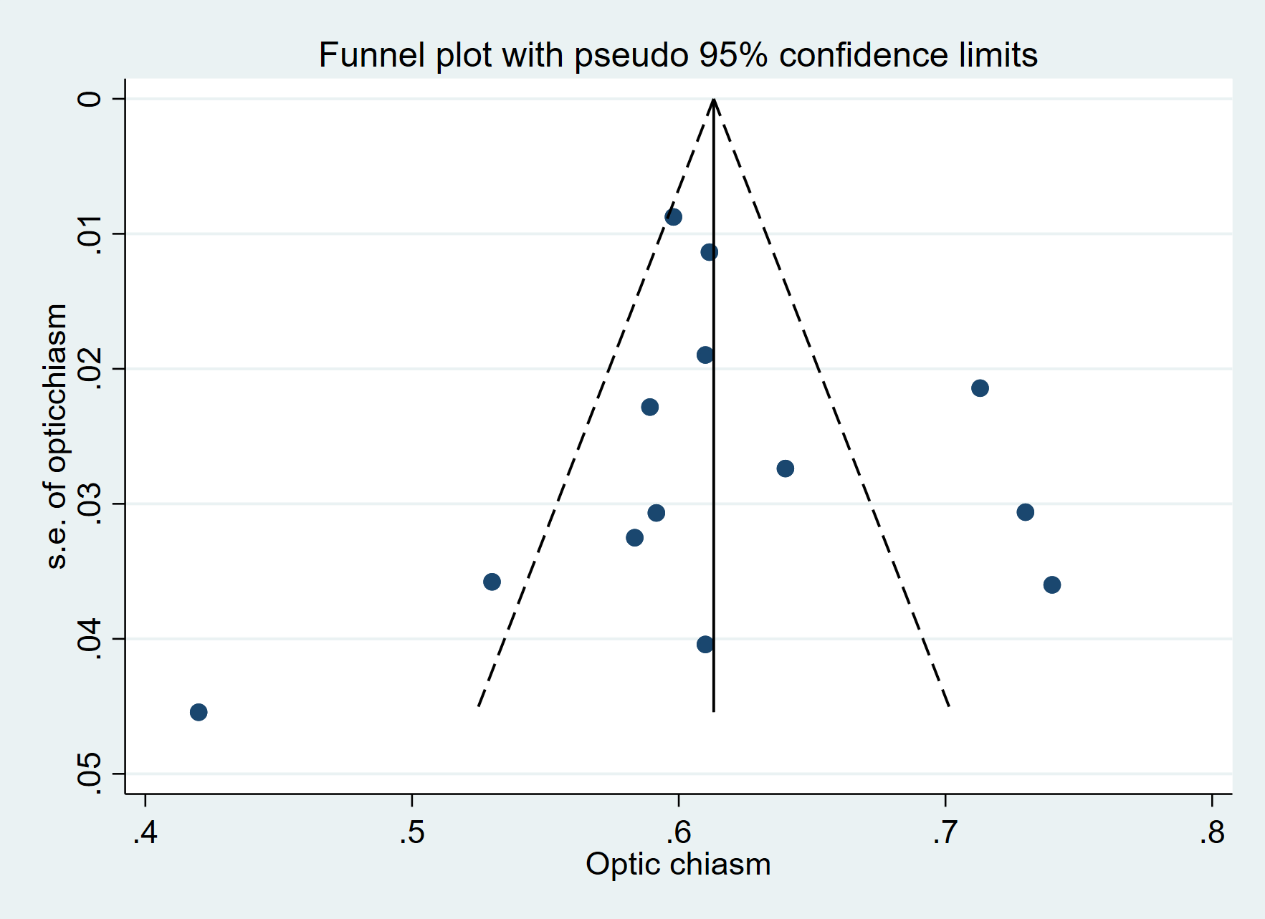


**Figure 2I** Funnel plots for meta-analysis of left parotid gland


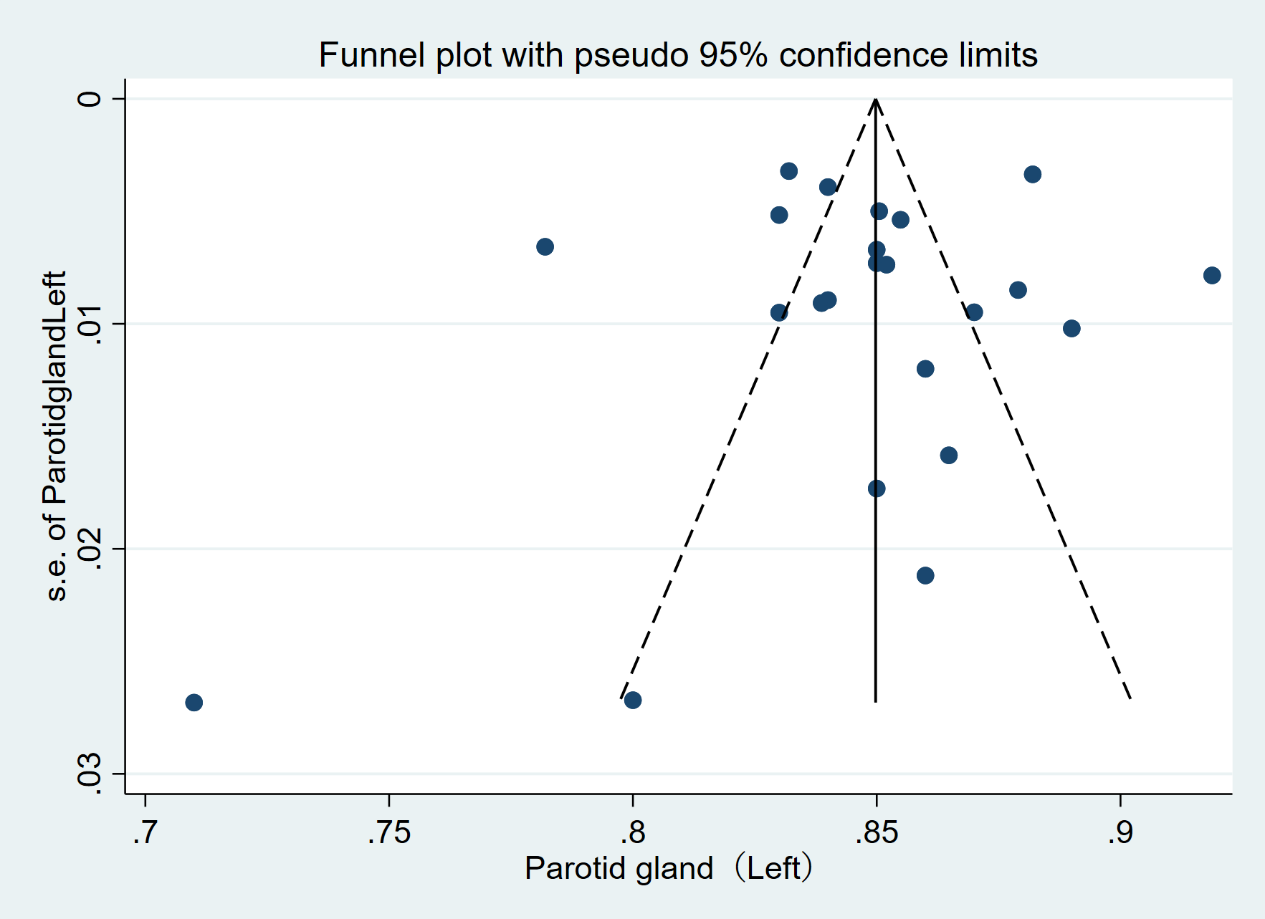


**Figure 2J** Funnel plots for meta-analysis of right parotid gland


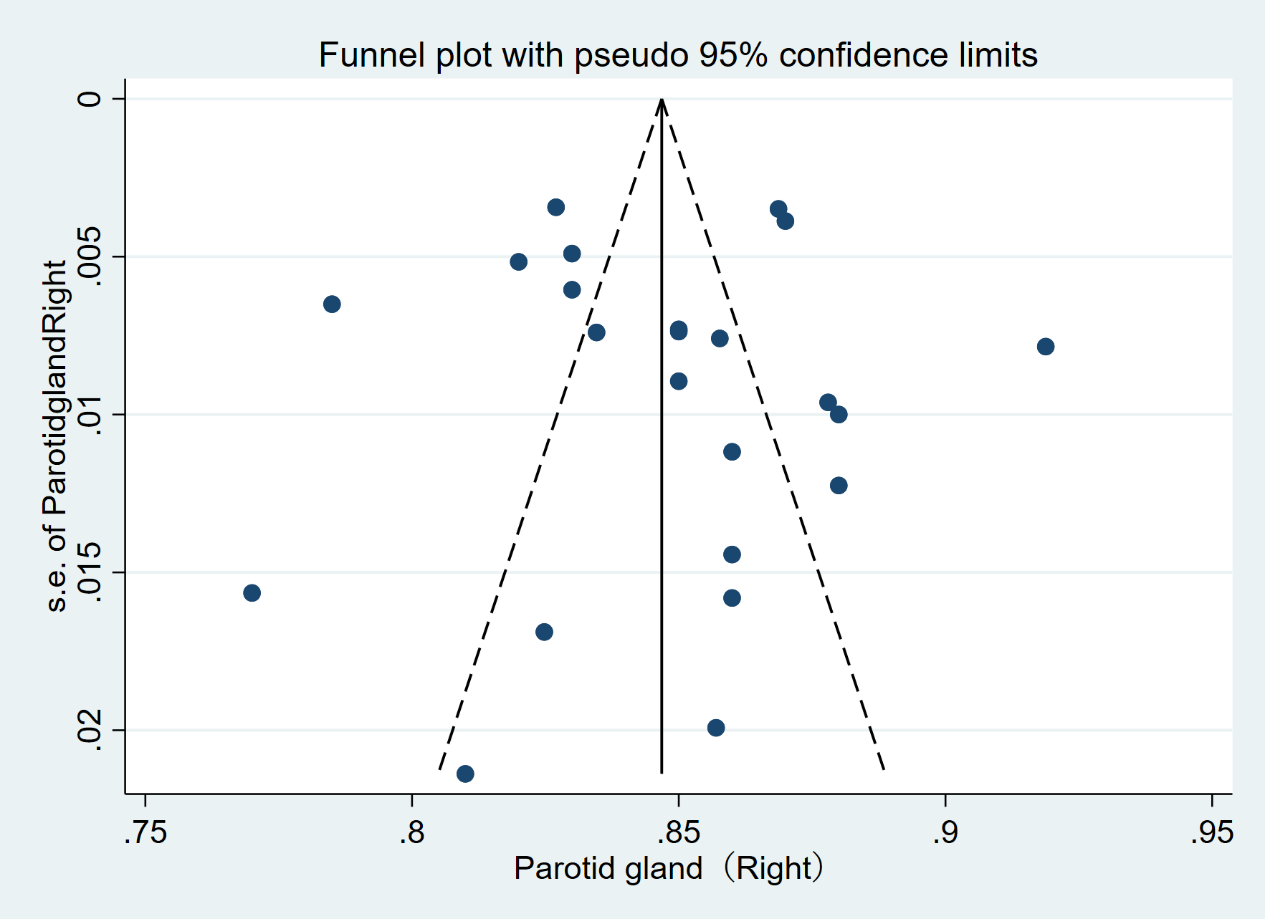


**Figure 2K** Funnel plots for meta-analysis of left submandibular gland


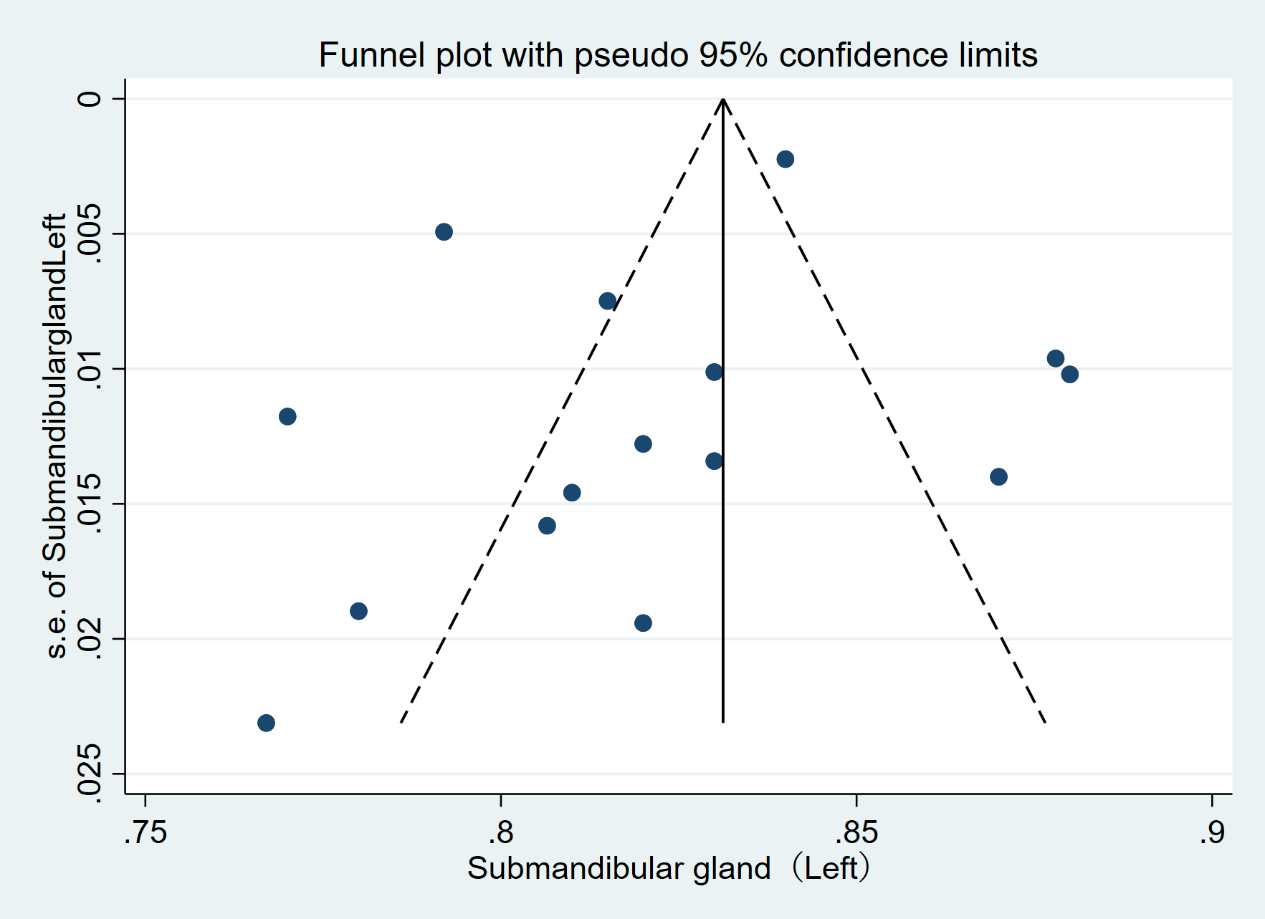


**Figure 2L** Funnel plots for meta-analysis of right submandibular gland


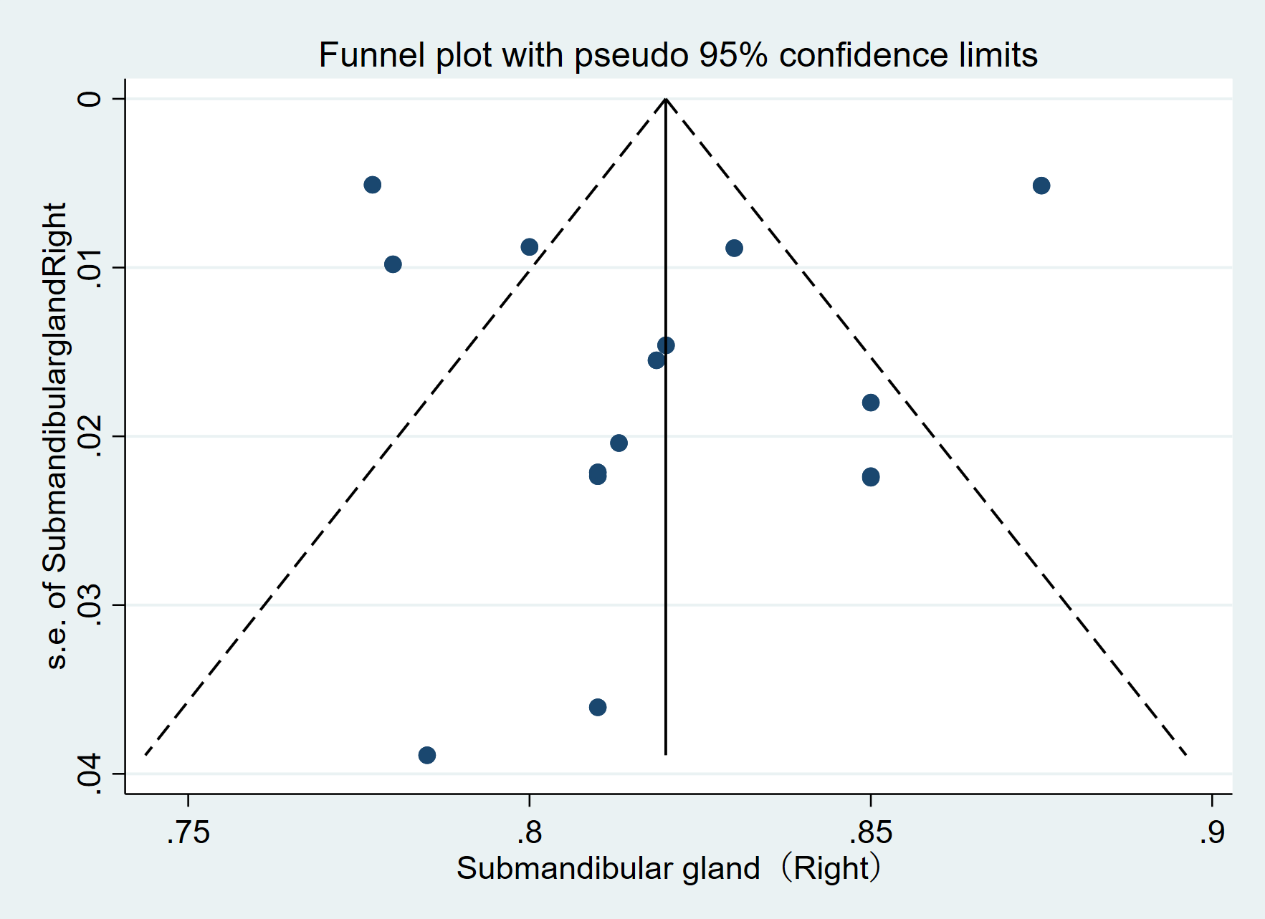


**Additional file 1: Figure S3(A-H)**Forest plot of the DSC of segmentation of 4 OARs in CT or MRI images

**Figure 3S** Forest plot of the pooled DSC of brain stem in CT images.


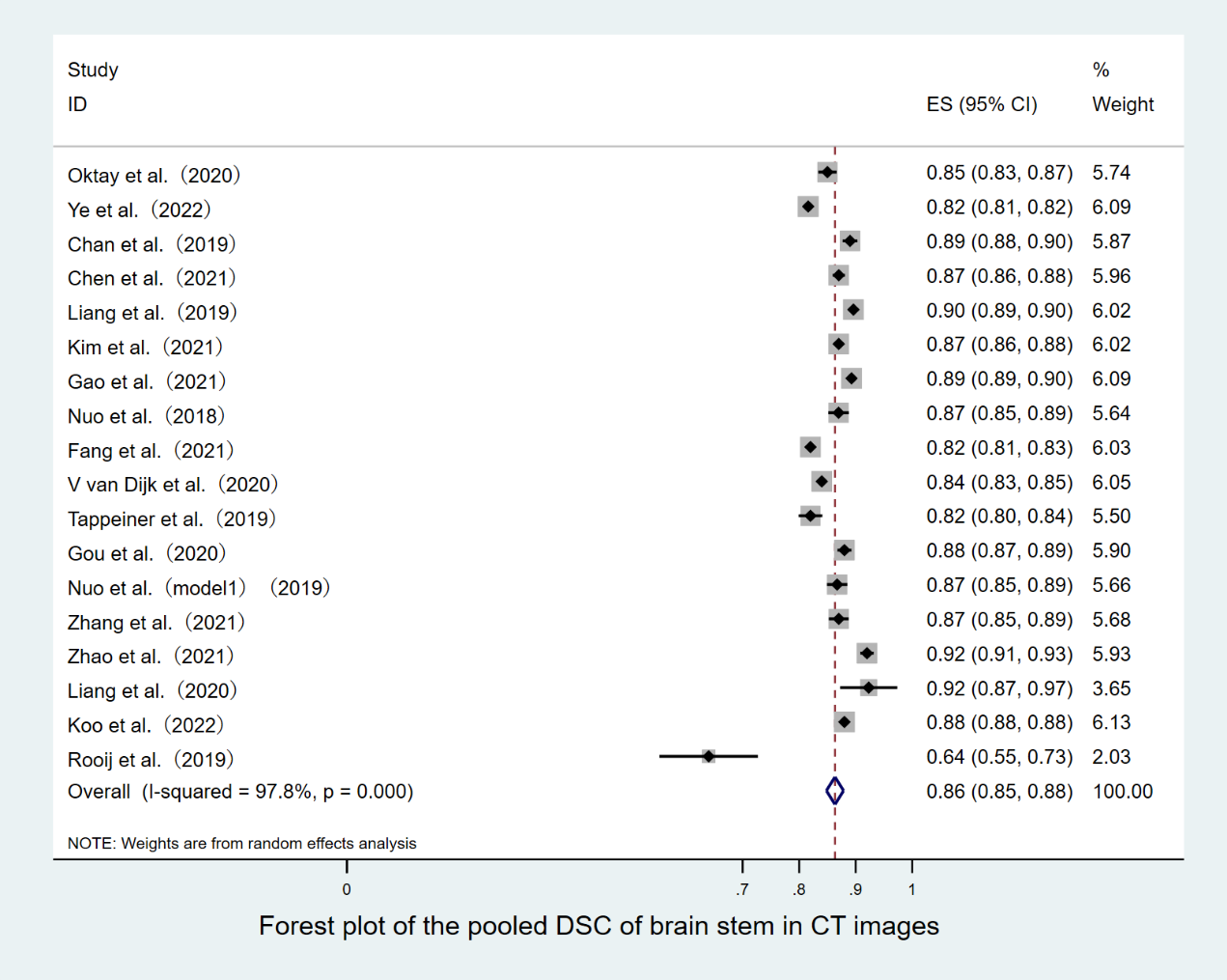


**Figure 3B** Forest plot of the pooled DSC of mandible in CT images.


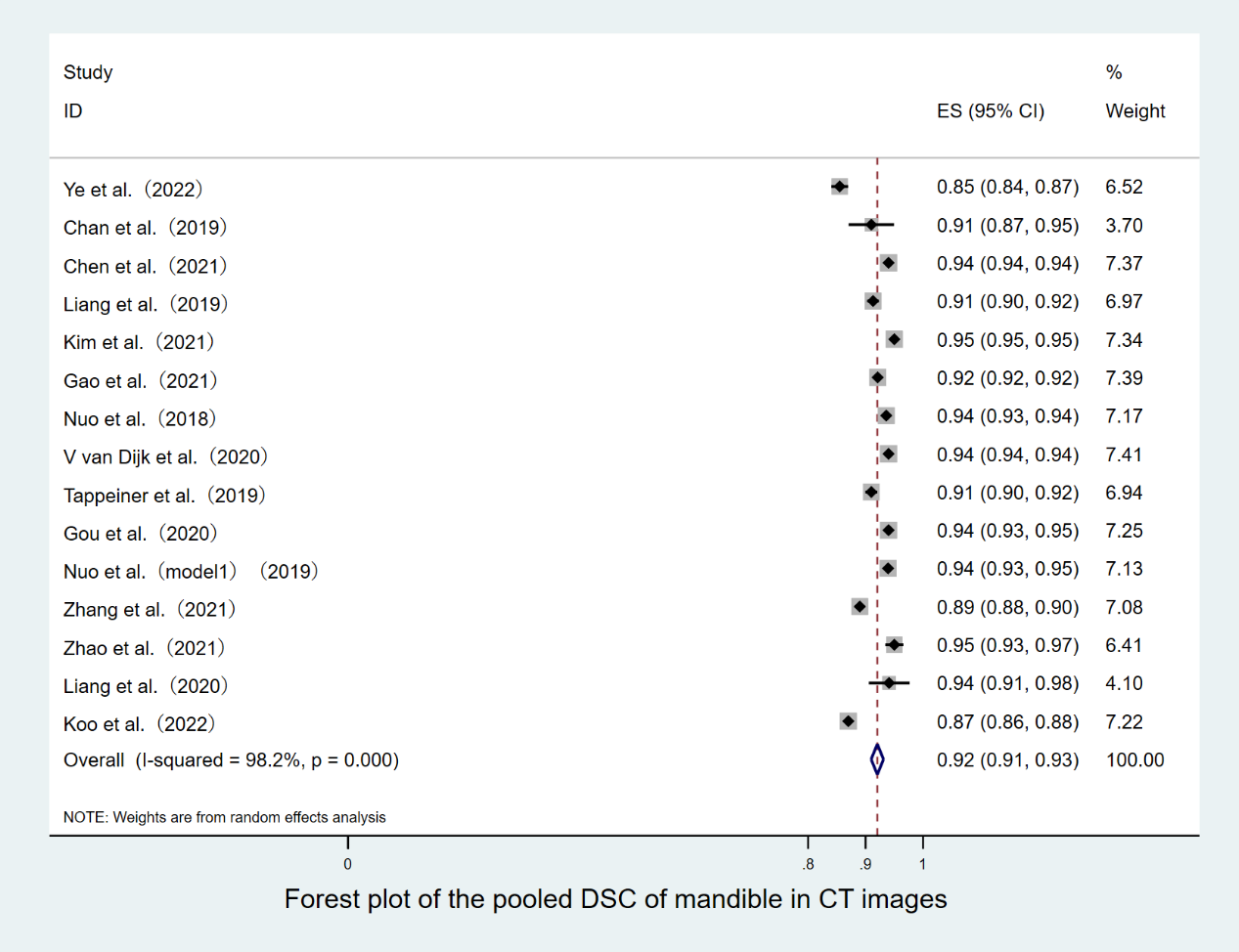


**Figure 3C** Forest plot of the pooled DSC of left optic nerve in CT images.


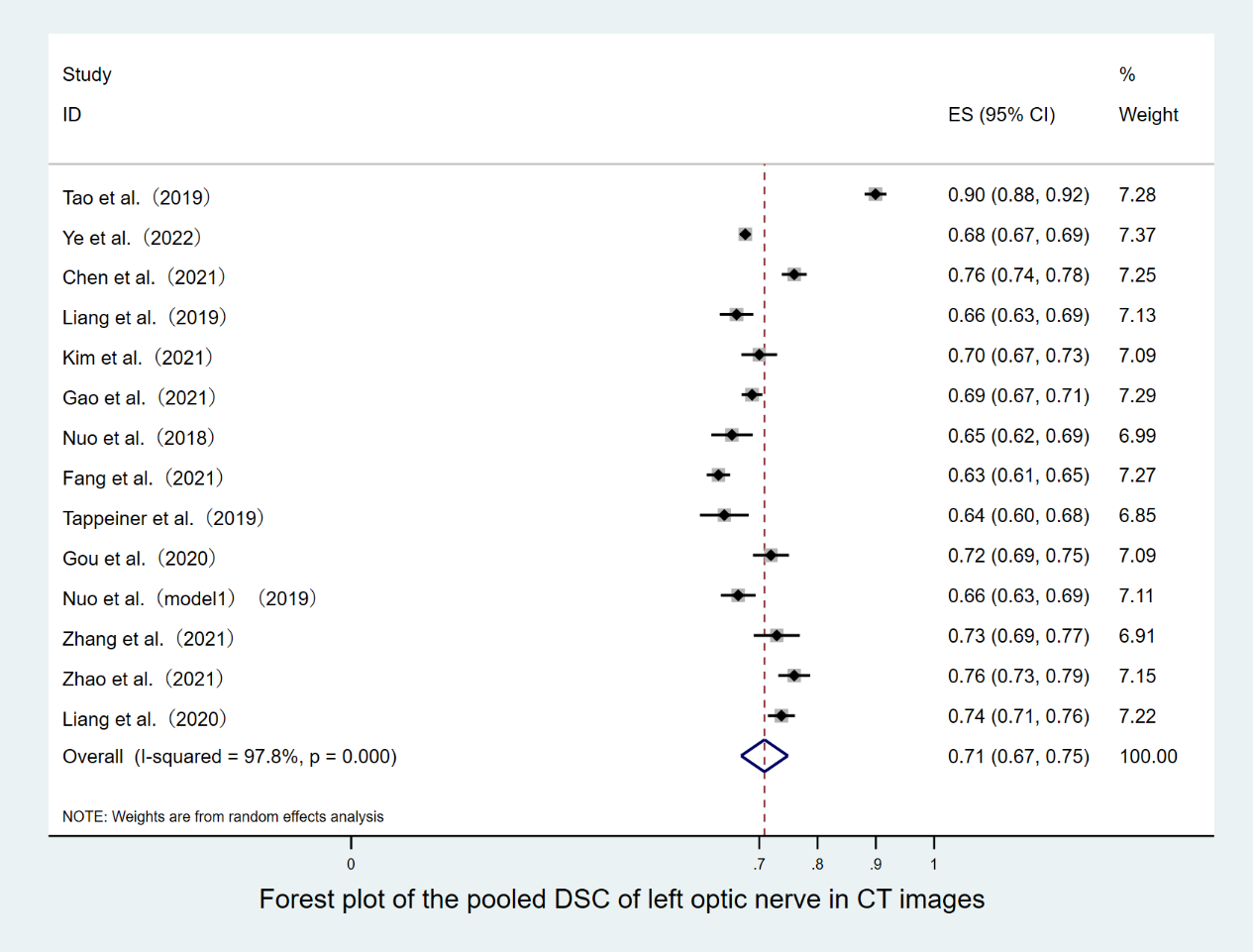


**Figure 3D** Forest plot of the pooled DSC of left parotid gland in CT images.


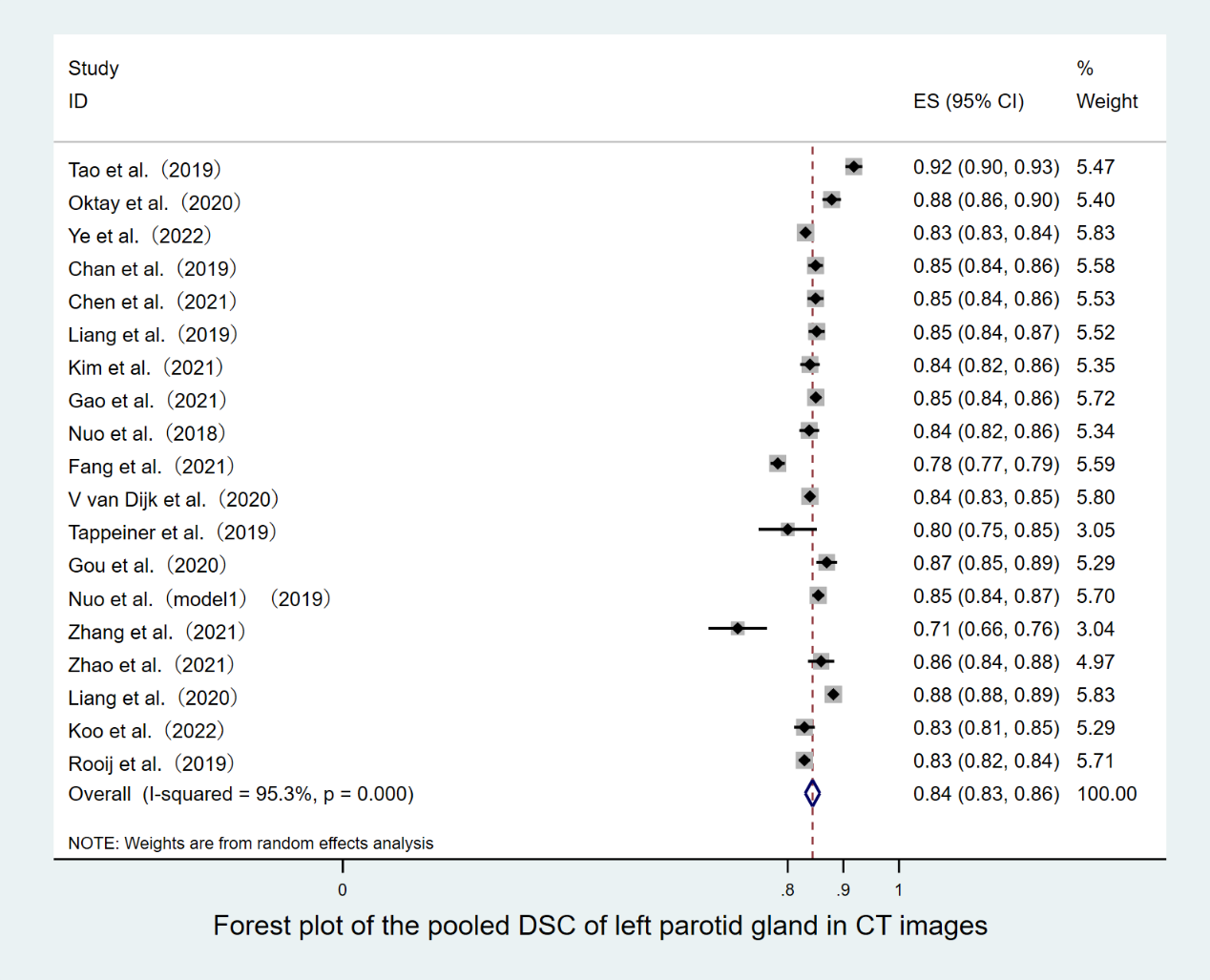


**Figure 3E** Forest plot of the pooled DSC of brain stem in MRI images.


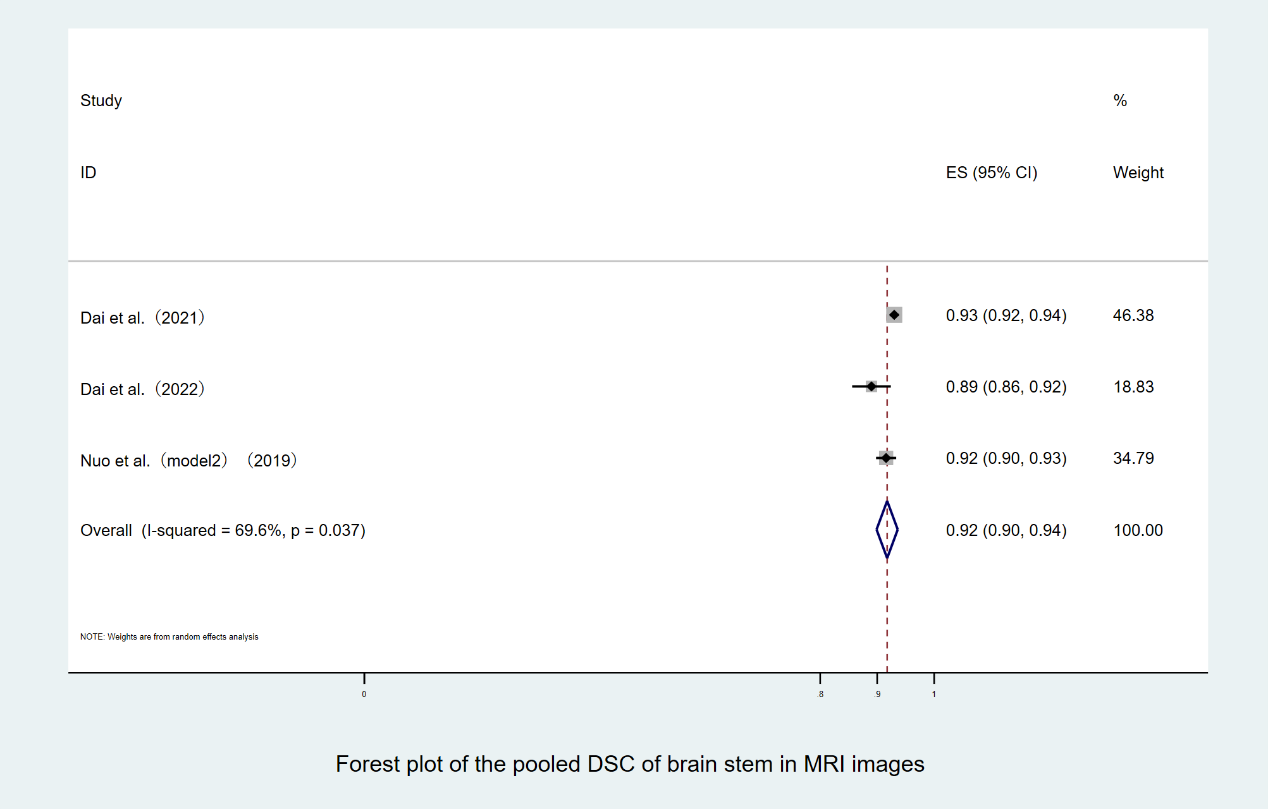


**Figure 3F** Forest plot of the pooled DSC of mandible in MRI images.


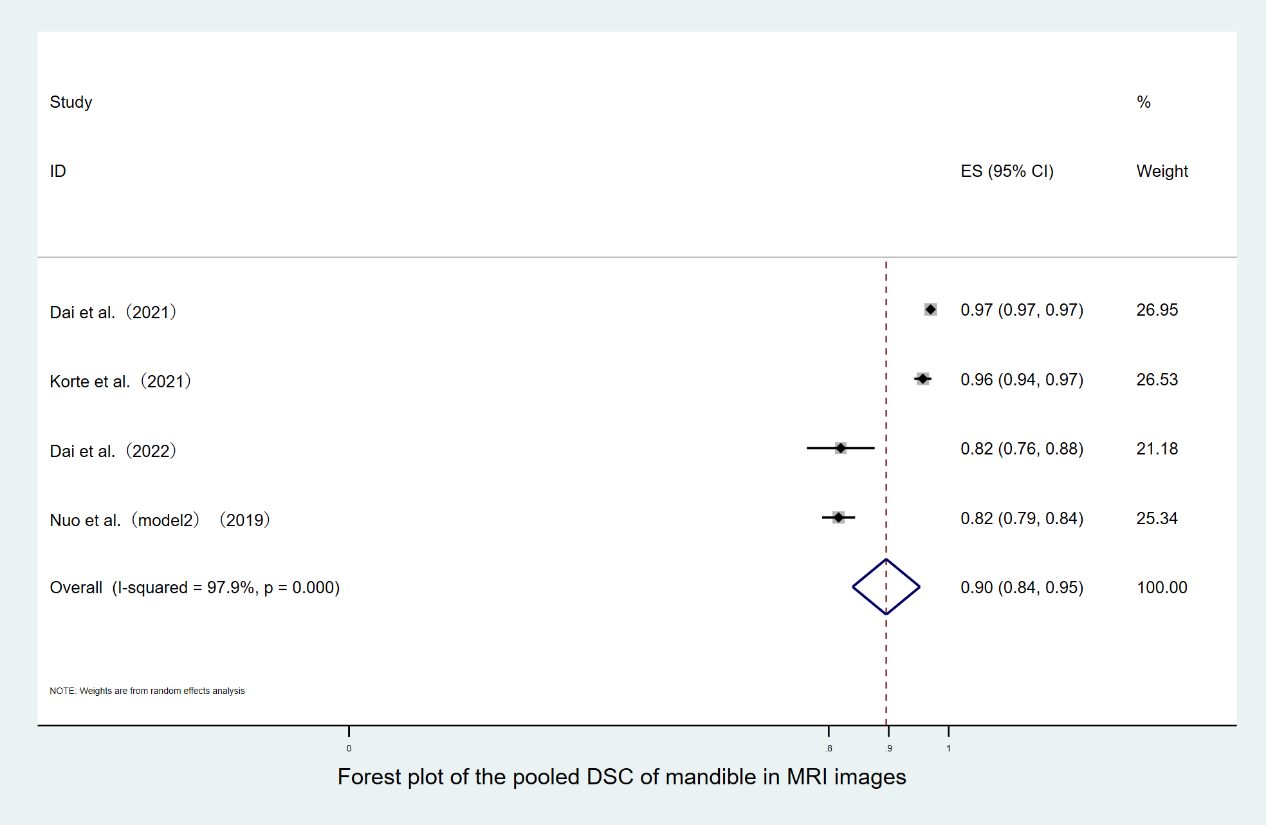


**Figure 3G** Forest plot of the pooled DSC of left optic nerve in MRI images.


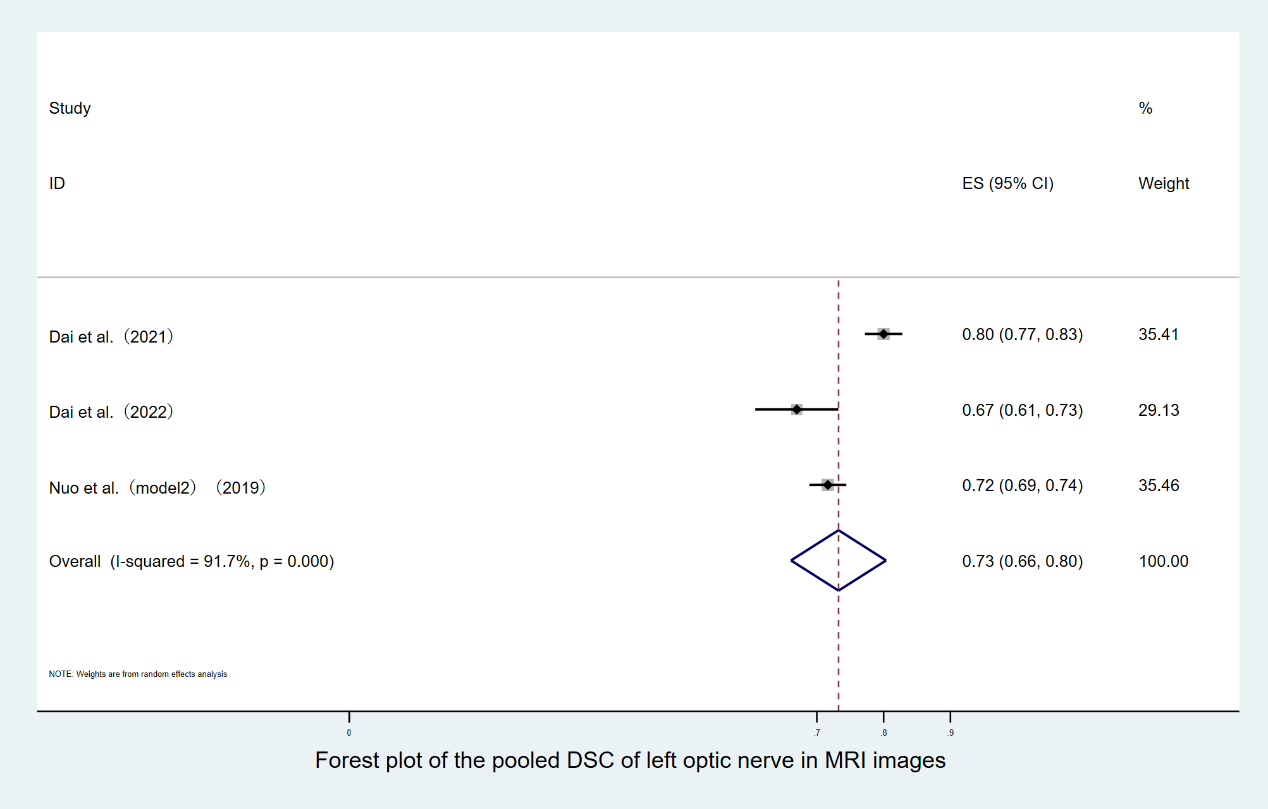


**Figure 3H** Forest plot of the pooled DSC of left parotid gland in MRI images.


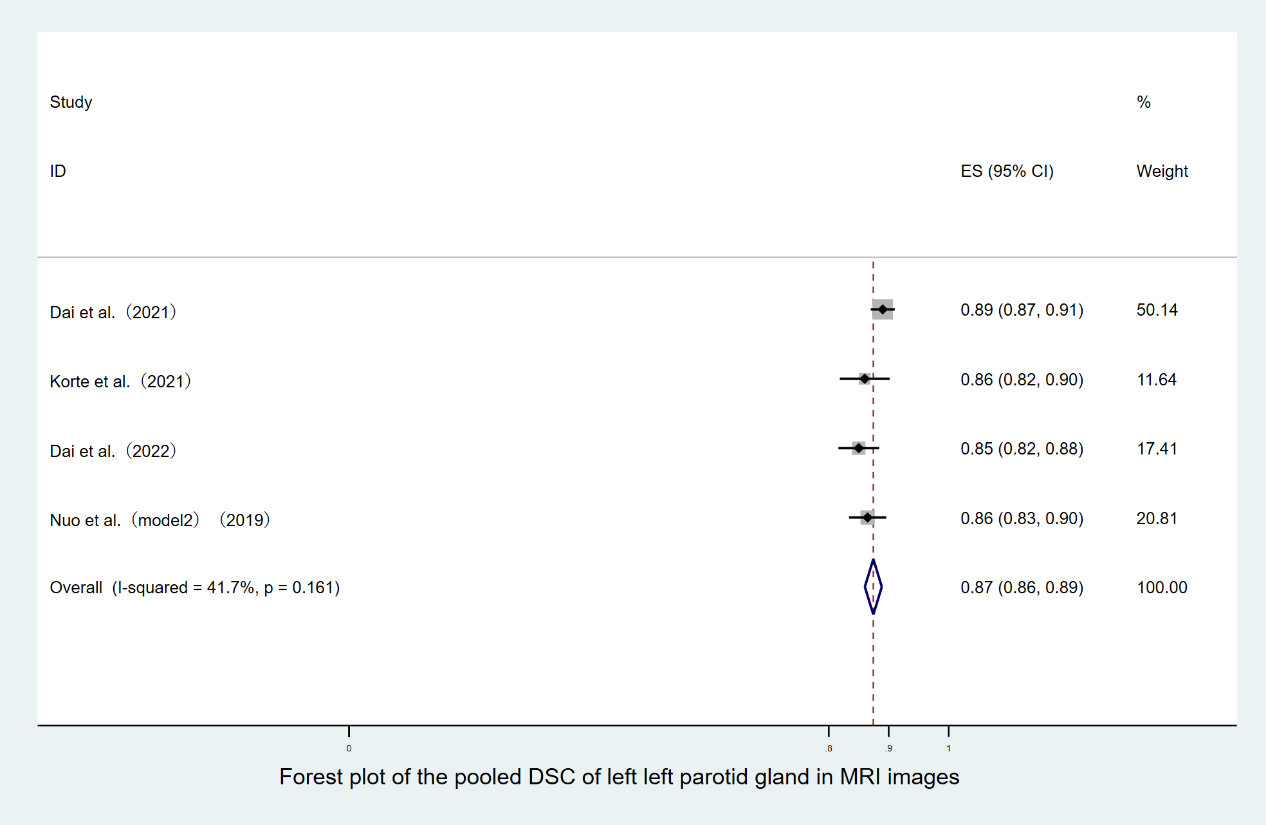


**Additional file 1: Figure S4** **(A-H)** Forest plot of the DSC of segmentation of 4 OARs in 2D or 3D images

**Figure 4A** Forest plot of the pooled DSC of brain stem in 2D images.


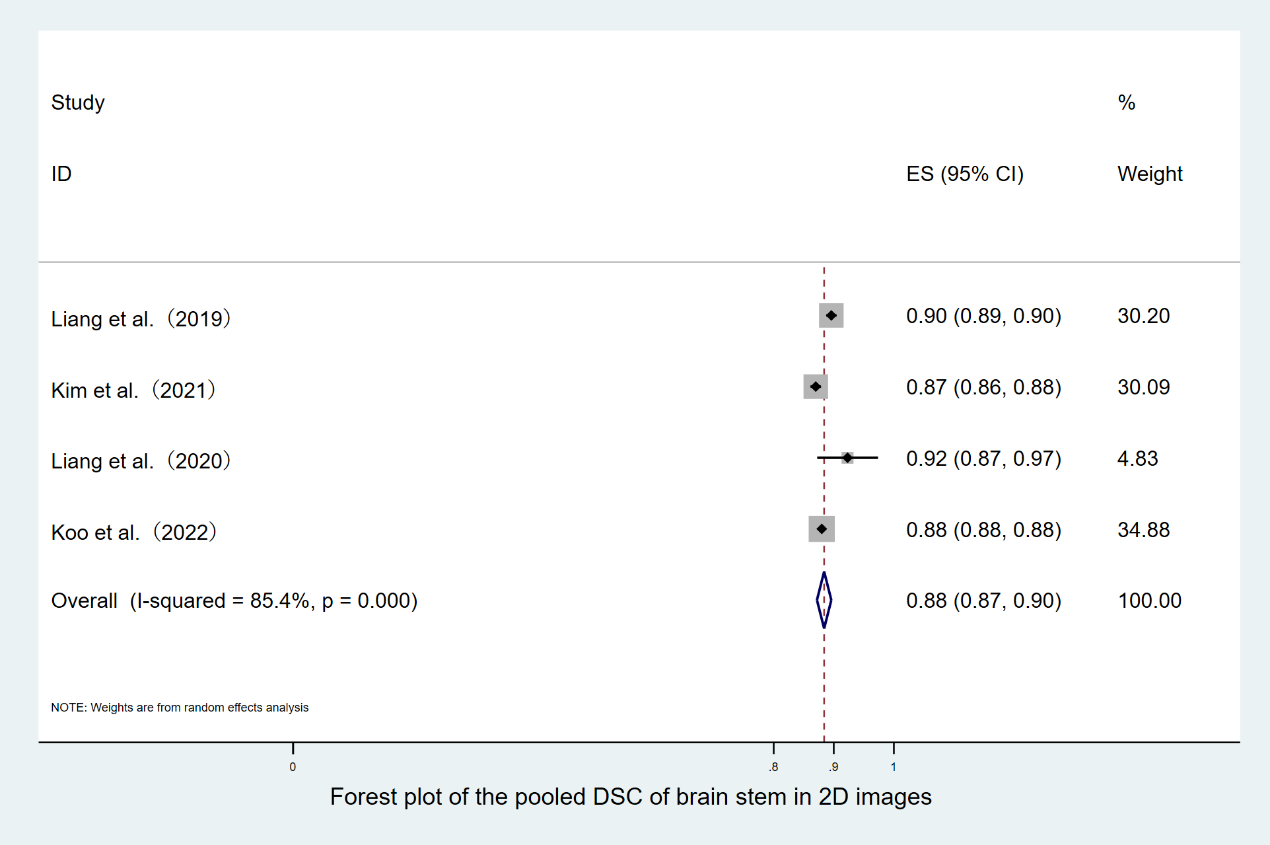


**Figure 4B** Forest plot of the pooled DSC of mandible in 2D images.


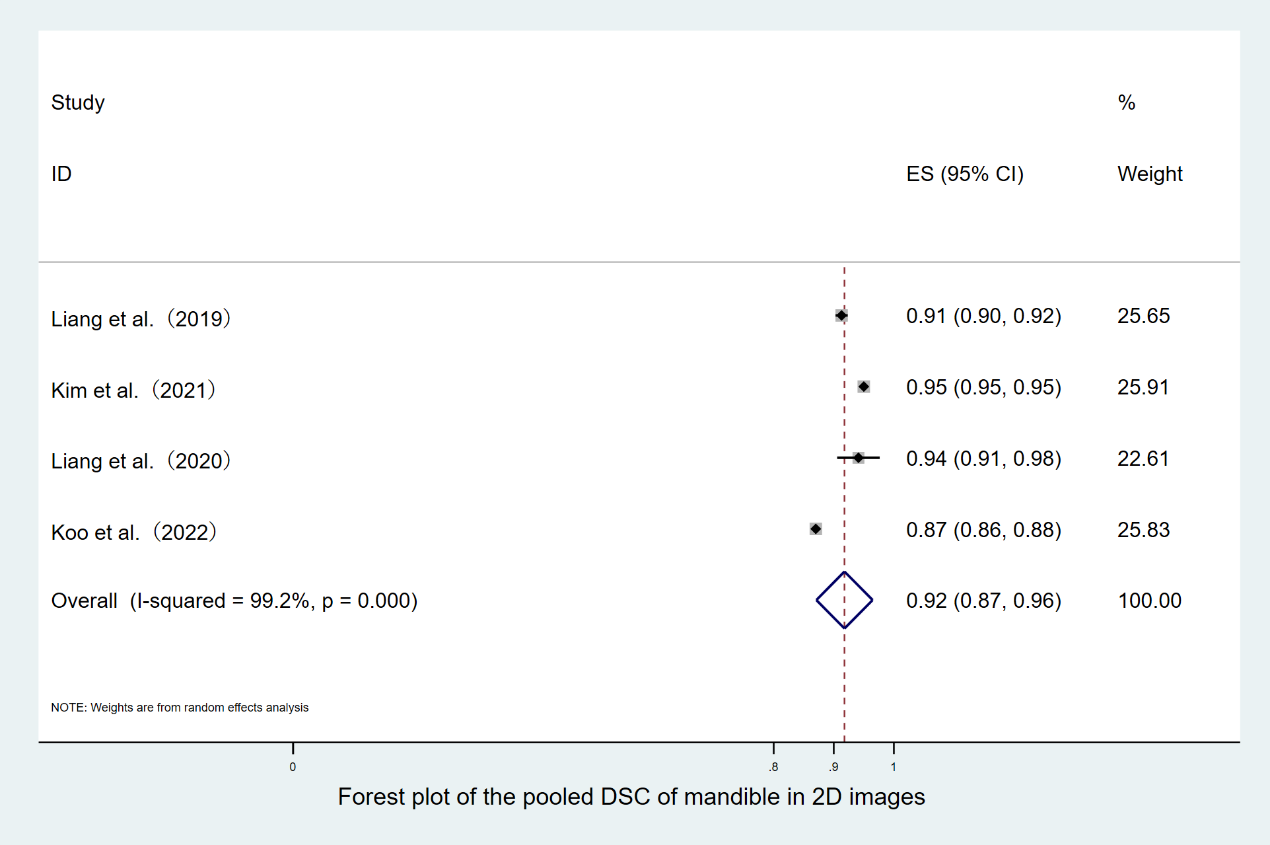


**Figure 4C** Forest plot of the pooled DSC of left optic nerve in 2D images.


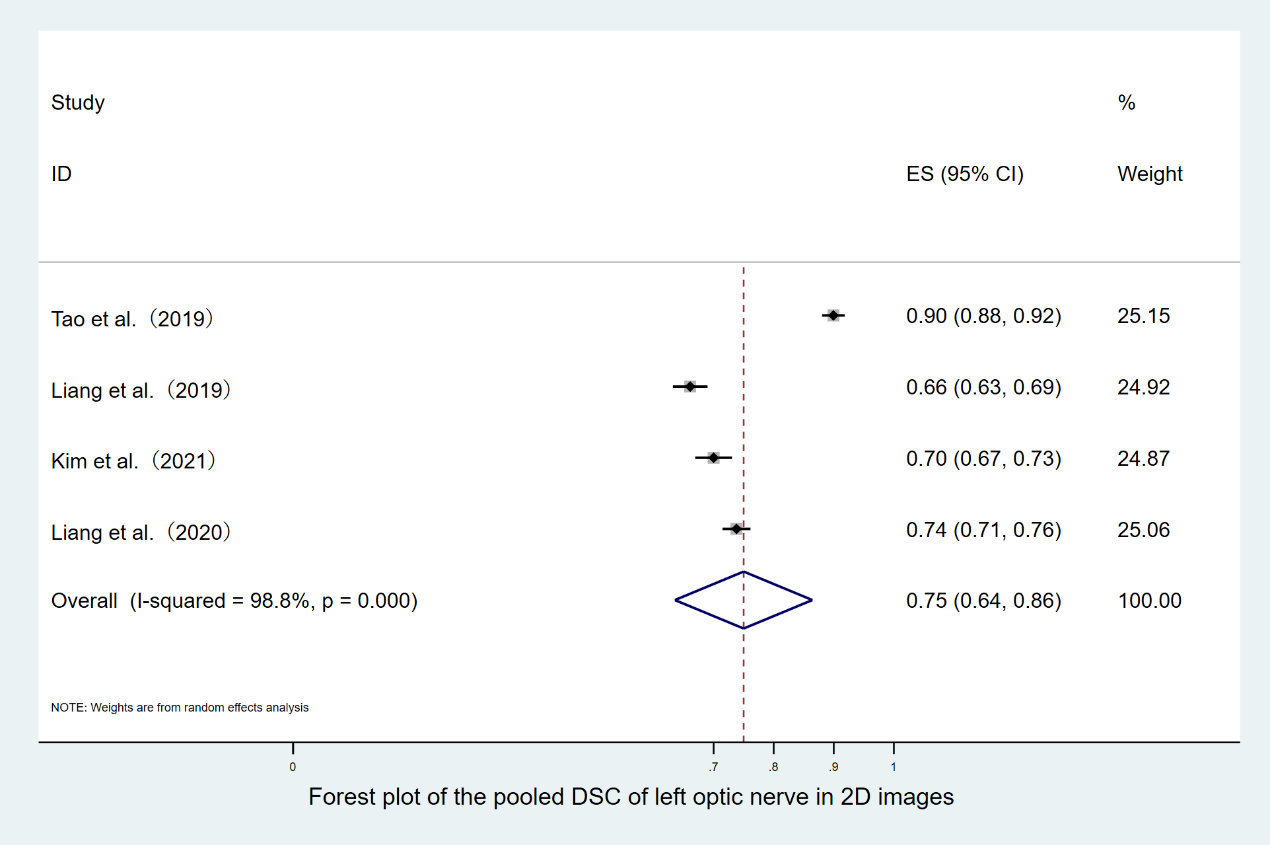


**Figure 4D** Forest plot of the pooled DSC of left parotid gland in 2D images.


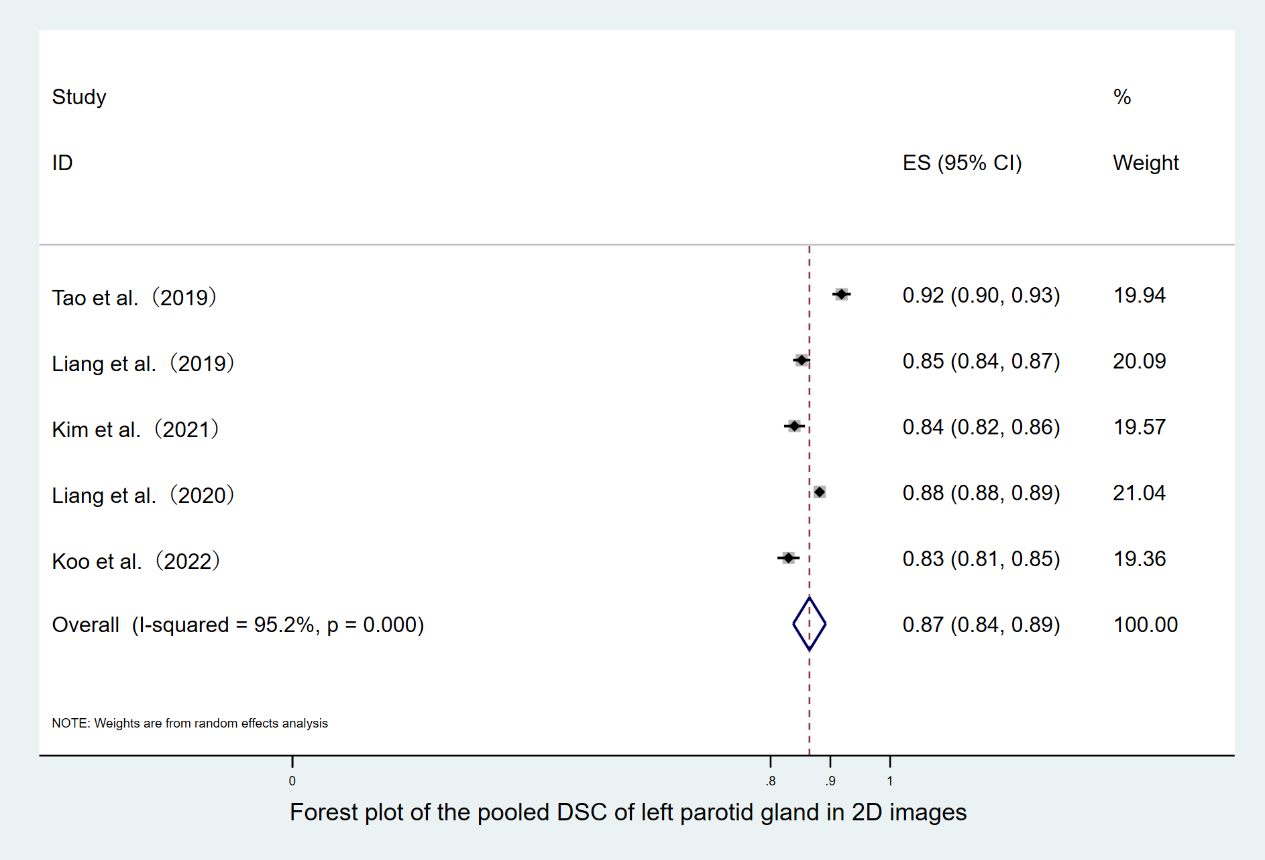


**Figure 4E** Forest plot of the pooled DSC of brain stem in 3D images.


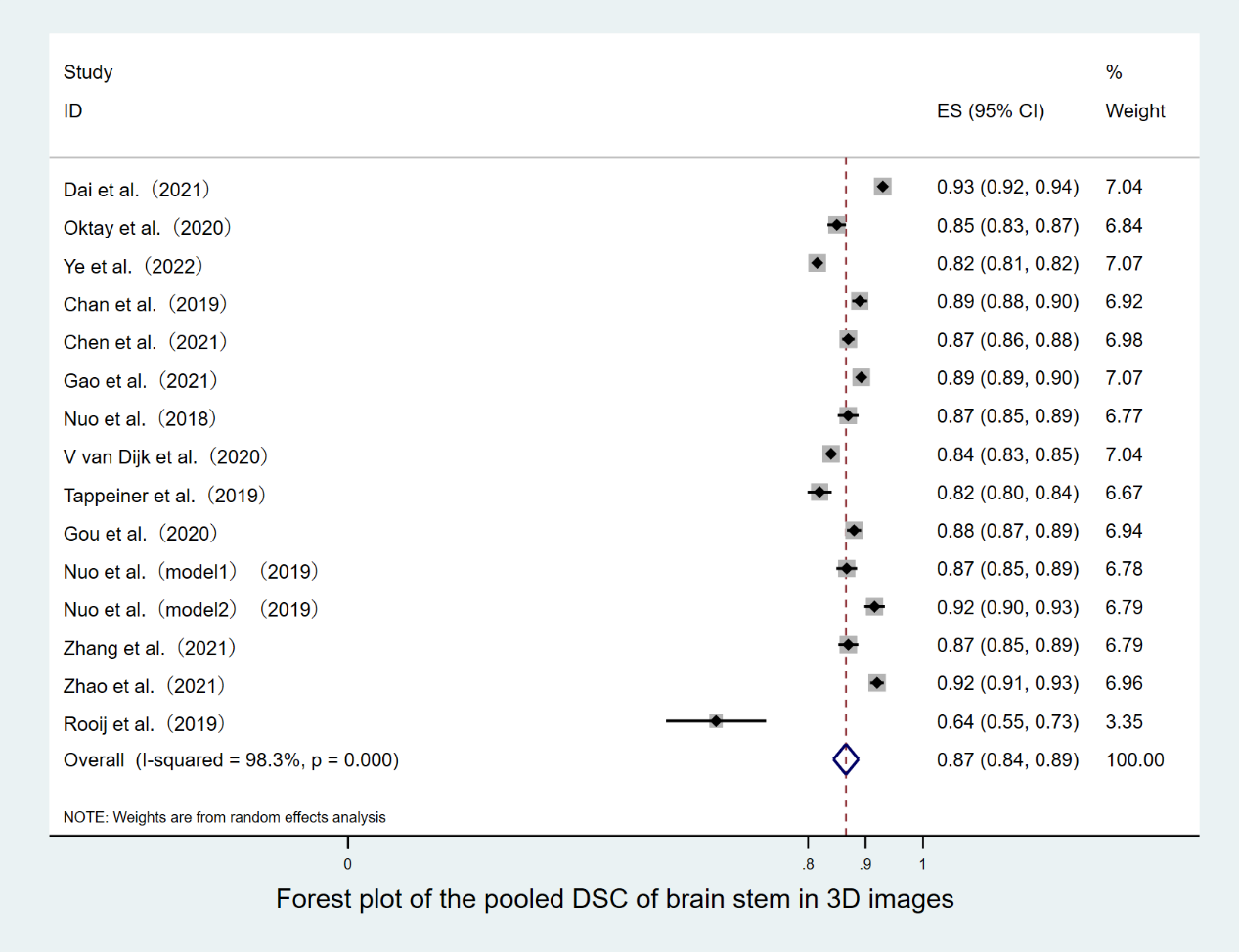


**Figure 4F** Forest plot of the pooled DSC of mandible in 3D images.


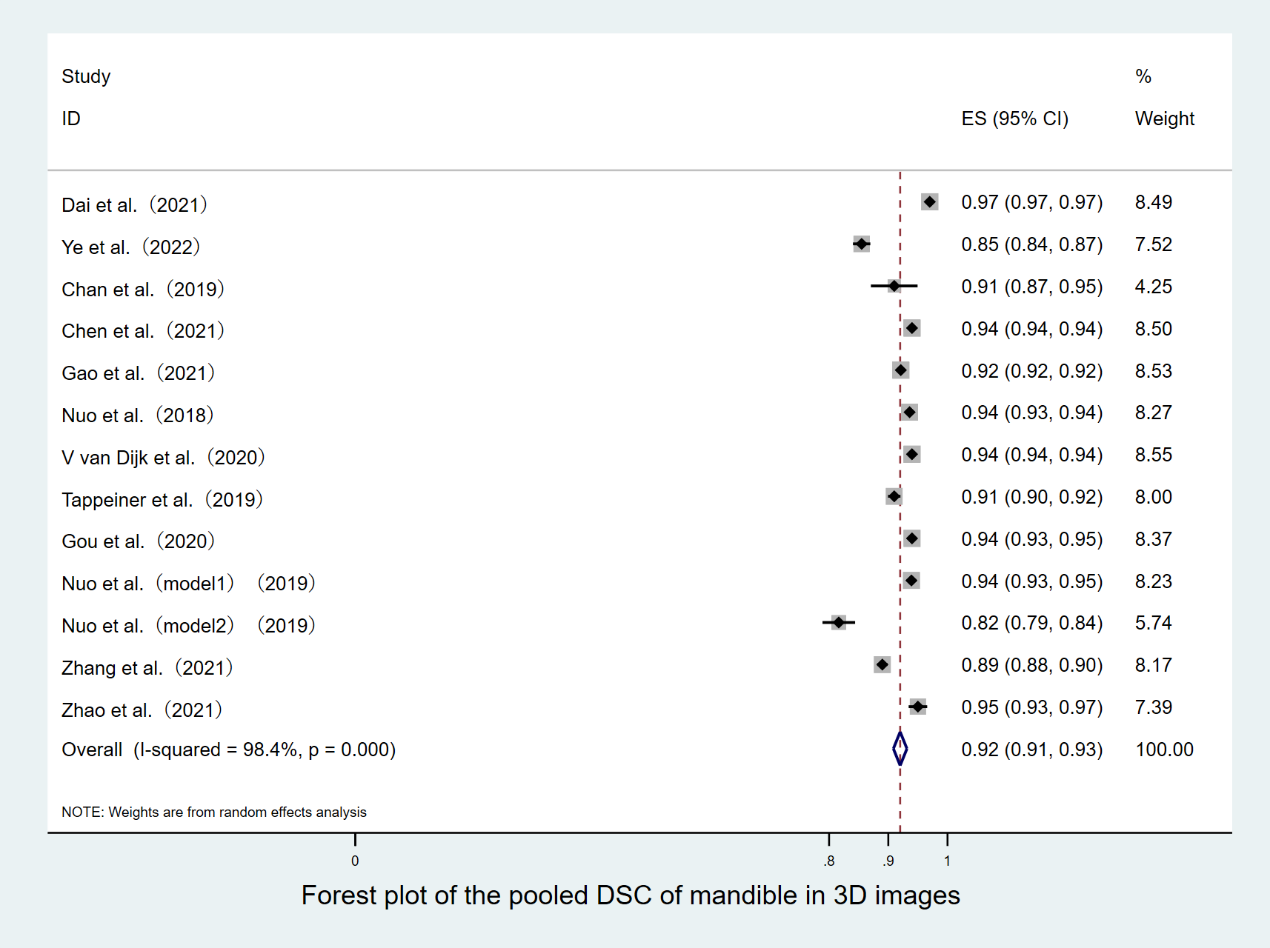


**Figure 4G** Forest plot of the pooled DSC of left optic nerve in 3D images.


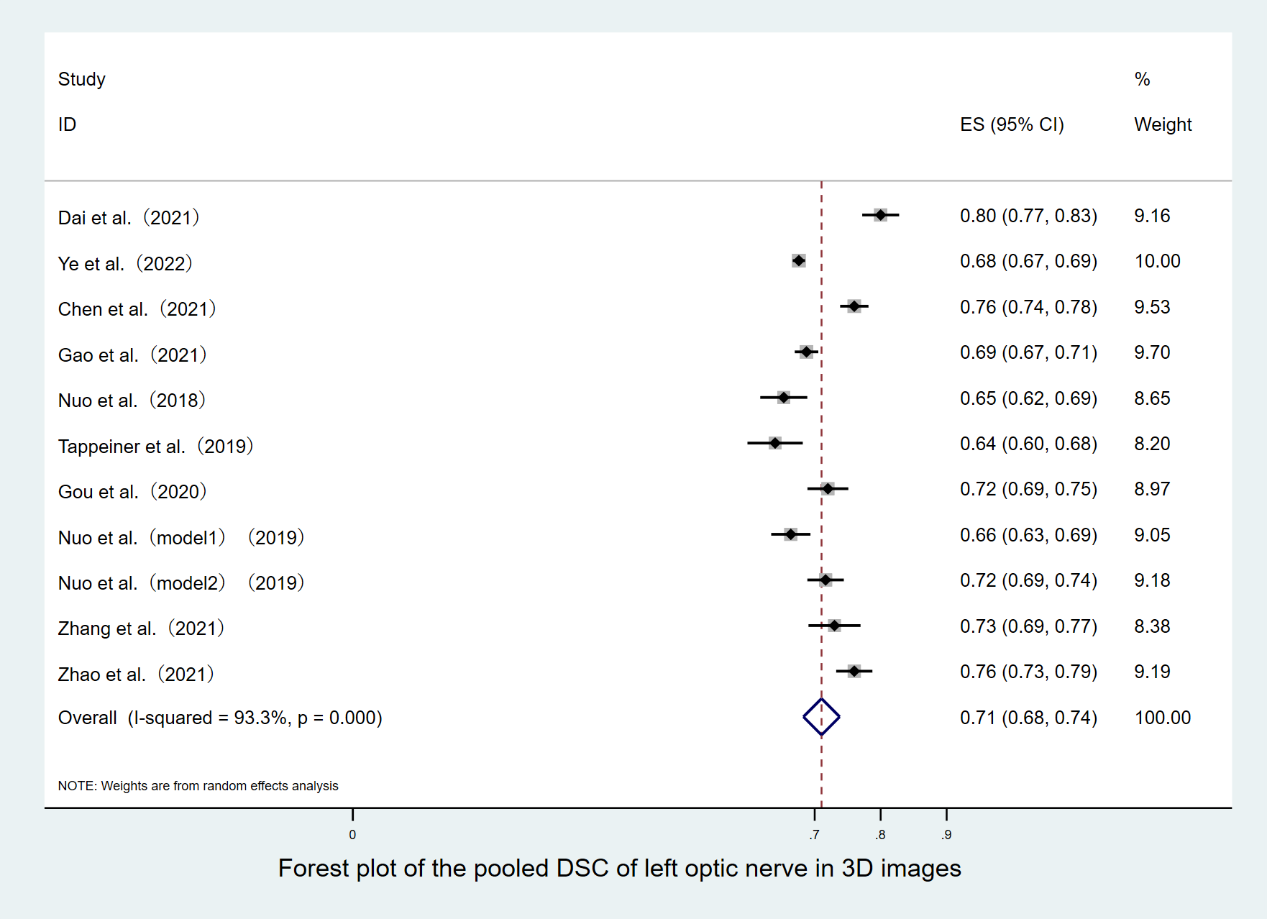


**Figure 4H** Forest plot of the pooled DSC of left parotid gland in 3D images.


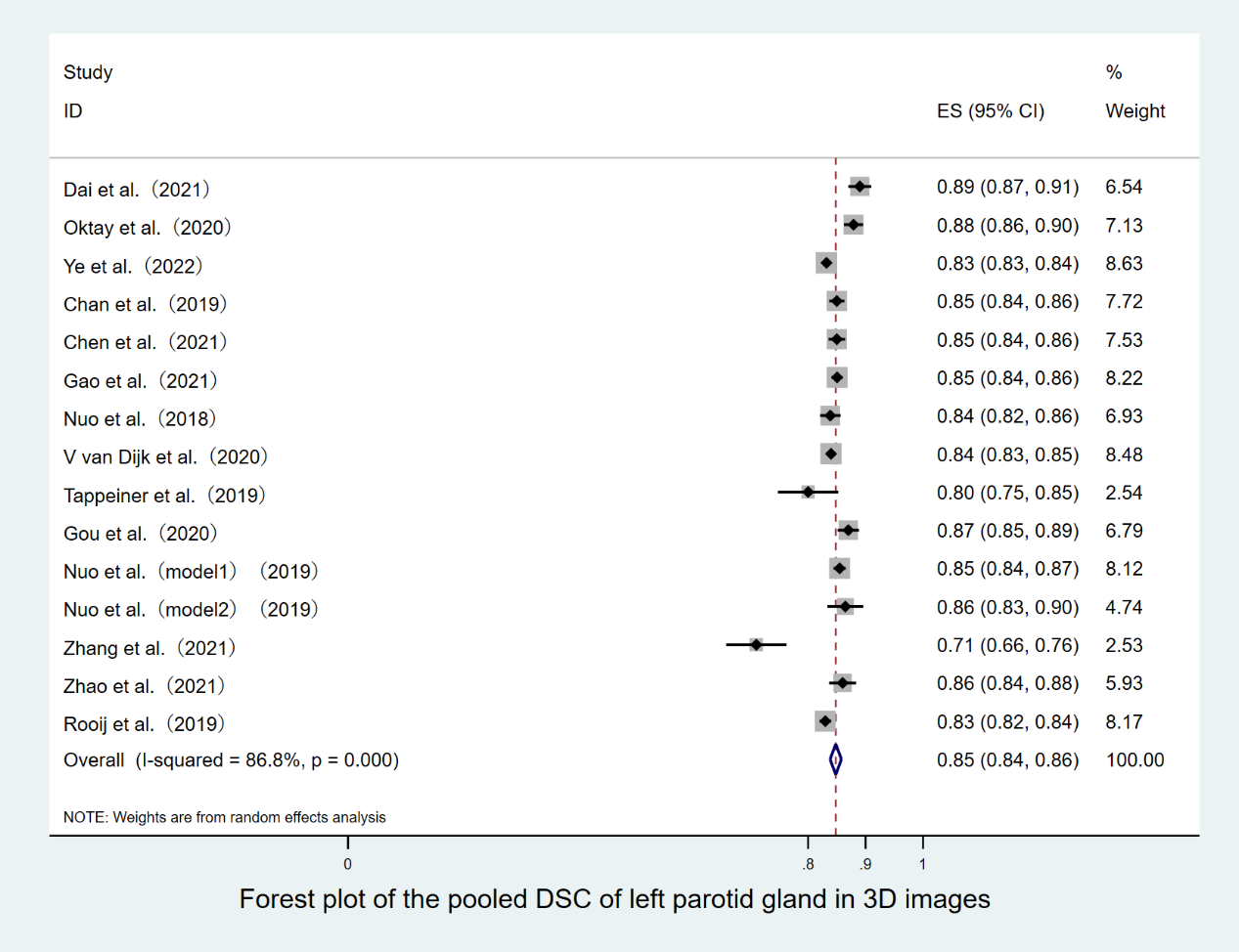

Supplement: Supplementary file 1 — Additional file 1: Table S1. Search strategies. Table S2. Checklist for Artificial Intelligence in Medical Imaging (CLAIM). Table S3. PROBAST (Prediction model Risk of Bias Assessment Tool) Review Items. Table S4. Result of CLAIM. Table S5. Result of PROBAST. Figure S1 (A–L) Forest plot of the pooled DSC of 12 OARs. Figure S2 (A–L) Funnel plots for meta-analysis of 12 OARs. Figure S3 (A–H) Forest plot of the DSC of segmentation of 4 OARs in CT or MRI images. Figure S4 (A–H) Forest plot of the DSC of segmentation of 4 OARs in 2D or 3D images. [file 12938_2023_1159_MOESM1_ESM.docx]
